# Oxidative coupling of methane—comparisons of MnTiO_3_–Na_2_WO_4_ and MnO_x_–TiO_2_–Na_2_WO_4_ catalysts on different silica supports

**DOI:** 10.1038/s41598-022-06598-6

**Published:** 2022-02-16

**Authors:** Worapinit Tiyatha, Thanaphat Chukeaw, Sarannuch Sringam, Thongthai Witoon, Metta Chareonpanich, Günther Rupprechter, Anusorn Seubsai

**Affiliations:** 1 Department of Chemical Engineering, Faculty of Engineering, Kasetsart University, Bangkok, 10900 Thailand; 2 Center of Excellence on Petrochemical and Materials Technology, Kasetsart University, Bangkok, 10900 Thailand; 3 Research Network of NANOTEC–KU on NanoCatalysts and NanoMaterials for Sustainable Energy and Environment, Kasetsart University, Bangkok, 10900 Thailand; 4 Institute of Materials Chemistry, TU Wien, 1060 Vienna, Austria

**Keywords:** Chemical engineering, Chemical engineering

## Abstract

The oxidative coupling of methane (OCM) converts CH_4_ to value-added chemicals (C_2+_), such as olefins and paraffin. For a series of MnTiO_3_-Na_2_WO_4_ (MnTiO_3_-NW) and MnO_x_-TiO_2_-Na_2_WO_4_ (Mn-Ti-NW), the effect of loading of MnTiO_3_ or MnO_x_-TiO_2_, respectively, on two different supports (sol–gel SiO_2_ (SG) and commercial fumed SiO_2_ (CS)) was examined. The catalyst with the highest C_2+_ yield (21.6% with 60.8% C_2+_ selectivity and 35.6% CH_4_ conversion) was 10 wt% MnTiO_3_-NW/SG with an olefins/paraffin ratio of 2.2. The catalyst surfaces with low oxygen-binding energies were associated with high CH_4_ conversion. Stability tests conducted for over 24 h revealed that SG-supported catalysts were more durable than those on CS because the active phase (especially Na_2_WO_4_) was more stable in SG than in CS. With the use of SG, the activity of MnTiO_3_-NW was not substantially different from that of Mn-Ti-NW, especially at high metal loading.

## Introduction

Methane (CH_4_) is a major chemical feedstock with a tetrahedral structure. The primary chemical conversions of CH_4_ include oxidation, syngas reforming, and halogenation^1^, which are difficult to control. The lifetime of CH_4_ in the environment is much shorter than that of CO_2_, but CH_4_ traps radiation more efficiently than CO_2_. Therefore, CH_4_ has a greater impact on global warming than the same amount of CO_2_^2^. Indeed, the cumulative influence of CH_4_ over 100 years is estimated to be 25 times greater than that of CO_2_^3^. In recent years, the total CH_4_ emissions have been found to result from natural gas and petroleum systems (30%), enteric fermentation (27%), landfills (17%), manure management (9%), and coal mining (7%), and others (10%)^3^. Processes for an efficient conversion of CH_4_ to useful chemicals are, therefore, of great interest to reduce the amount of CH_4_ released to the atmosphere.

Methane can be used both *indirectly* and *directly* to produce high-value hydrocarbons, such as olefins and paraffin = C_2+_. The indirect methods use methane steam reforming, dry reforming, or partial oxidation as the first process for the production of synthesis gas (CO and H_2_). Subsequently, Fischer–Tropsch and methanol synthesis processes are applied to generate value-added products^4^. The indirect methods are currently commercially applied in the petrochemical industry^5^, but direct methods would be clearly beneficial.

A feasible route is the direct conversion of methane to C_2+_ via oxidative coupling of methane (OCM) either by heterogeneous catalytic or homogeneous non-catalytic processes. The OCM reaction occurs at high temperatures (700–900 °C) at atmospheric pressure and requires only oxygen as the co-reactant in an inert gas (such as nitrogen) feed. As the non-catalytic process suffers from low methane conversion and selectivity, a heterogeneous catalytic process is desirable. Therefore, having a suitable solid catalyst is a prerequisite for the catalytic OCM process before commercialization to become industrially viable.

The OCM reaction is capable of generating C_2+_, specifically ethylene (C_2_H_4_), but yields below 20% prevent commercial OCM. Under appropriate process conditions, yields of 30% have been reported in laboratory tests, with a minimum C_2_H_4_ yield of 25% required to make the method economically feasible (35–50% would be more practical)^6^. Generally, to be commercially attractive, C_2+_ selectivity and CH_4_ conversion in OCM must exceed 80% and 30%, respectively^7^, which has stimulated research for many years^8^. However, despite exhaustive efforts, the OCM reaction still lacks a highly active and stable catalyst with high performance for C_2+_ formation.

Previous studies have indicated that Na_2_WO_4_-MnO_x_-SiO_2_-based catalysts were among the most active ones that yielded high C_2+_ selectivity at high CH_4_ conversions, resulting in a C_2+_ yield of 10–35%^9–19^. The high performance of these catalysts resulted from a complex combination of several factors, as inferred from literature. Some interesting facts on the active components are detailed as follows:

i. Na atoms are necessary for the phase transition of amorphous SiO_2_ to crystalline α-cristobalite at low calcination temperature (800 °C)^20,21^. It is noteworthy that the usual temperature for this phase change is approximately 1500 °C^22^. Additionally, the mobility of Na^+^ species during OCM at high temperatures (750–850 °C) can create active sites on the catalyst surface, facilitating hydrogen abstraction of CH_4_^23^. Moreover, it was suggested that Na^+^ aids in the stability of the active WO_4_ phase^9,24^. Furthermore, it was claimed that active Na_2_O species can be generated in small amounts in the catalytically relevant temperature regime (above 600 °C), and these Na_2_O species are responsible for high activity and C_2+_ selectivity^25^.
ii. In a study of Na_2_WO_4_/SiO_2_ using temporal analysis of products (TAP) and steady-state OCM reaction studies, the Na-WO_x_ sites on the surface were indicated to be responsible for the selective activation of CH_4_ to C_2_H_x_ and over-oxidization of CH_y_ to CO. On the other hand, the molten Na_2_WO_4_ phase promotes the oxidative dehydrogenation of C_2_H_6_ to C_2_H_4_, but it is also responsible for the over-oxidation of CH_4_ to CO_2_^26^.
iii. WO_4_ and MnO_x_ are the crucial active components for the generation of C_2+_ products. They cooperate during the reaction as follows. Initially, the O^2-^ species associated with the surface WO_4_ (W^6+^) sites, especially the tetrahedral form^27^, activates CH_4_ into a gaseous methyl radical. As a result, the W^6+^ center is transformed to W^5+^ with one chemical bond of W–OH. Thereafter, the oxidation of W^5+^ to W^6+^ occurs through electron transfer from W^5+^ to Mn^3+^, subsequently reducing Mn^3+^ to Mn^2+^. Finally, the oxidation of Mn^2+^ to Mn^3+^ occurs via reaction with gaseous oxygen, which generates an OH radical and an active oxygen atom^28–30^. In other words, the MnO_x_ species boosts the oxygen mobility in the catalyst, resulting in an improved exchange of gaseous and surface oxygen^31^. Additionally, an in-depth study of Mn addition to 5Na_2_WO_4_/SiO_2_ catalyst revealed that Mn enhances the formation of both dissolved O_2_ and lattice atomic O species, which are responsible for catalyzing the OCM reaction. Therefore, the CH_4_ conversion toward C_2+_ formation is improved^32,33^.
iv. The addition of TiO_2_ into the MnO_x_-Na_2_WO_4_/SiO_2_ catalyst further reduces the temperature of CH_4_ activation in the OCM reaction to approximately 650–720 °C. This is probably due to the formation of a MnTiO_3_ phase that enables the transition of Mn^2+^ to Mn^3+^ at a lower temperature than that of the MnO_x_ phase^34^.
v. The α-cristobalite SiO_2_ is considered to assist CH_4_ activation (although the underlying mechanism is unclear)^15,20^, but upon calcination (starting with amorphous SiO_2_), the surface area of the support declines substantially^14,35^. Accordingly, some reports have shown that the phase transformation during the reaction causes catalyst deactivation^27,36^. Other causes for catalyst deactivation may include the decomposition of active phases (such as Na_2_WO_4_)^37–39^ and/or loss of Na_2_O due to sublimation during the reaction^25^.

In several other studies, catalysts containing Mn, Na, Ti, W, and/or mesoporous SiO_2_ have been claimed as highly active for OCM^27,29,34,38–42^ with MnTiO_3_, MnO_x_, and TiO_2_ components being recommended as critical active phases for CH_4_ activation. Many studies have attempted to further improve the C_2+_ selectivity and CH_4_ conversion of Na_2_WO_4_-MnO_x_-SiO_2_-based catalysts by understanding the mechanism over the surface. For example, a study on the addition of H_2_O to the testing system revealed that H_2_O did not change the overall scheme of product formation but it was able to reduce the contribution of direct oxidation of CH_4_ to CO_2_^43^. The effect of pressure was studied on the performance of Mn/Na_2_WO_4_/SiO_2_, which revealed that the increase of pressure leads to higher C_2+_ selectivity and can accelerate unselective gas-phase reactions more than surface catalyzed processes^19^. It is also possible that the CH_4_ conversions of 60–75% range could be obtained at a maximized C_2+_ yield in each specific reactor setup^19^. Our previous investigation of MnTiO_3_ and Na_2_WO_4_ on different silica-based supports (fumed SiO_2_, MCM-41, and SBA-15) showed that the presence of MnTiO_3_ and Na_2_WO_4_ on SBA-15 substantially increased CH_4_ activation of CH_3_ and H radicals, causing the OCM reaction to efficiently generate C_2+_ hydrocarbons^27^. However, the MnTiO_3_ nanocomposite has been less studied than the MnO_x_ + TiO_2_ nanocomposite. Furthermore, the preparation of sol–gel SiO_2_ has never been combined with such catalysts. We have speculated that a catalyst prepared using the sol–gel SiO_2_ may resist catalyst deactivation better when fumed SiO_2_ is used. Therefore, the current study aimed for a systematic comparison of the activity/selectivity of MnTiO_3_-Na_2_WO_4_ and MnO_x_-TiO_2_-Na_2_WO_4_ supported on different silica-based supports (sol–gel SiO_2_ and commercial fumed SiO_2_). The results should reveal: (i) Whether the MnTiO_3_ phase indeed functions better in the OCM reaction than the nanocomposite mixture of MnO_x_ and TiO_2;_ and (ii) Whether the type of SiO_2_ support has a substantial influence on catalytic performance.

## Results and discussion

### Activity of catalysts in the OCM reaction

The nanocomposite MnTiO_3_-NW/CS, MnTiO_3_-NW/SG, and Mn-Ti-NW/SG catalysts, containing 0–20 wt% of MnTiO_3_ or Mn-Ti, respectively, were tested for the OCM reaction (Table 1, Supplementary Figure S1). Considering the catalyst performance in terms of C_2+_ yield per catalyst mass, when the amount of MnTiO_3_ or Mn-Ti increased from 0 to 5 or 10 wt%, respectively, the corresponding activities suddenly increased, but then maintained the maximum values for higher loadings. The highest C_2+_ yields achieved were 20.6% (58.0% C_2+_ selectivity with 35.5% CH_4_ conversion) for 5 wt% MnTiO_3_-NW/CS, 21.6% (60.8% C_2+_ selectivity with 35.6% CH_4_ conversion) for 10 wt% MnTiO_3_-NW/SG, and 21.5% (60.5% C_2+_ selectivity with 35.5% CH_4_ conversion) for 20 wt% Mn-Ti-NW/SG. The activity of MnTiO_3_-NW/CS gradually diminished as the MnTiO_3_ loading exceeded 10 wt%, whereas the activities of MnTiO_3_-NW/SG and Mn-Ti-NW/SG (both with the sol–gel SiO_2_ support) hardly changed.

**Table 1:** Performance of OCM catalysts.

Catalyst	Olefins selectivity (%)	Paraffins selectivity (%)	Olefins/paraffins (mol/mol)	C_2+_ selectivity (%)	CH_4_ conversion (%)	C_2+_ yield (%)	r_C2+_ *
NW/CS	18.2	13.7	1.3	33.1	21.9	7.2	0.42
5MnTiO_3_-NW/CS	37.2	17.6	2.1	58.1	35.5	20.6	0.60
10MnTiO_3_-NW/CS	37.6	17.0	2.2	57.8	35.7	20.6	0.40
15MnTiO_3_-NW/CS	34.2	17.5	2.0	54.7	34.4	18.8	0.28
20MnTiO_3_-NW/CS	27.6	15.2	1.8	45.0	31.7	14.4	0.17
NW/SG	14.5	12.1	1.2	27.5	20.5	5.6	0.33
5MnTiO_3_-NW/SG	32.2	18.8	1.7	53.8	29.9	16.2	0.47
10MnTiO_3_-NW/SG	39.1	18.1	2.2	60.8	35.6	21.6	0.42
15MnTiO_3_-NW/SG	39.0	18.0	2.2	60.5	35.5	21.4	0.31
20MnTiO_3_-NW/SG	39.6	18.1	2.2	61.3	35.3	21.6	0.25
5Mn-Ti-NW/SG	38.5	17.0	2.3	59.0	35.4	20.9	0.60
10Mn-Ti-NW/SG	37.9	17.1	2.2	58.5	35.4	20.7	0.40
15Mn-Ti-NW/SG	38.3	17.6	2.2	59.3	35.0	20.8	0.30
20Mn-Ti-NW/SG	39.4	17.5	2.3	60.5	35.5	21.5	0.24

Testing conditions: 50 mg catalyst, gas feed of CH_4_:O_2_:N_2_ = 3:1:4, reactor temperature = 700 °C, atmospheric pressure, total gas flow rate = 50 mL min^-1^ (GHSV = 30,588 h^-1^).

*r_C2+_  = moles of C_2+_/[(total moles of MnTiO_3_ or (Mn + Ti) and Na_2_WO_4_) × h].

**Scheme 1:**
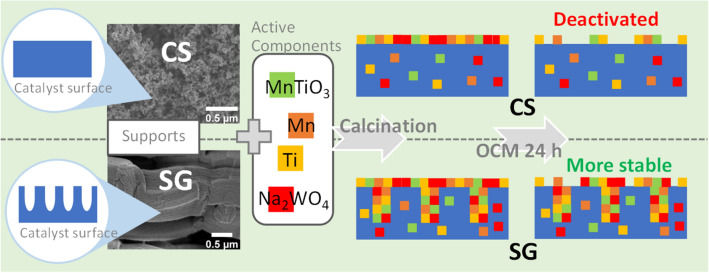
Models for the stability of the catalysts using CS- and SG-supports.

For the OCM reaction, designing a catalyst with high olefin selectivity is still challenging. Olefins, especially ethylene and propylene, have been important raw chemicals since the beginning of the chemical industry in the 1920s. Various downstream products of olefins, such as polyethylene, polypropylene, polyvinyl chloride, and ethanol), are utilized worldwide^44^. In Table 1, the catalytic performance is presented in terms of olefins/paraffin selectivity and olefins/paraffin ratio. It is important to note that the number (5, 10, 15, and 20) preceding each catalyst name refers to the loading of MnTiO_3_ or Mn-Ti, with “0wt%” omitted. The results indicated that NW/CS and NW/SG had relatively low olefins/paraffin ratios of 1.3 and 1.2, respectively. The 5MnTiO_3_-NW/CS and 10MnTiO_3_-NW/CS catalysts had relatively high olefins/paraffin ratios of 2.1, and 2.2, respectively, with the highest C_2+_ yield of 20.6%. When the MnTiO_3_ amount increased over 10 wt%, the olefins/paraffin ratio slightly decreased, corresponding to a higher CO_x_ selectivity. For MnTiO_3_-NW/SG and Mn-Ti-NW/SG, the olefins/paraffin ratios changed negligibly (2.2–2.3) as the amount of MnTiO_3_ and Mn-Ti increased. The difference in the behaviors of olefins/paraffin ratio at high loadings could be related to the distribution of the active metals on each support. The active metals of the catalysts using SG could be more well-dispersed due to the high porosity of the SG (see more detail in the discussion of Scheme 1). Considering the performance between MnTiO_3_-NW and Mn-Ti-NW on SG, at the same loading, the performance between these two had no significant difference, except at 5wt% loading 5MnTiO_3_-NW/SG exhibited a slightly lower performance. The catalysts producing the maximum yield in each group (5MnTiO_3_-NW/CS, 10MnTiO_3_-NW/SG, and 20Mn-Ti-NW/SG) exhibited high levels of dehydrogenation resulting in olefins/paraffin ratios of 2.1–2.3. However, when considering the C_2+_ formation rate (r_C2+_)—total moles of C_2+_ per total moles of MnTiO_3_ or (Mn + Ti) and Na_2_WO_4_ per h—of each catalyst (see Table 1), 5MnTiO_3_-NW/CS, 5MnTiO_3_-NW/SG, and 5Mn-Ti-NW/SG exhibited the highest r_C2+_ in each group. This suggests that the accessible active sites of the catalysts at high loadings (>5 wt%) are limited, possibly because of the low surface area and lack of pores of the catalysts. Nonetheless, this present work considers the catalyst performance in terms of C_2+_ yield per catalyst mass, and thus 5MnTiO_3_-NW/CS, 10MnTiO_3_-NW/SG, and 20Mn-Ti-NW/SG were chosen for further characterization.

### Catalyst characterization

The most promising catalysts from Sect. “Activity of catalysts in the OCM reaction” were further analyzed to explain their performance via their properties. The selected catalysts included the basic catalysts (NW/CS and NW/SG), the catalysts producing the maximum yield in each group (5MnTiO_3_-NW/CS, 10MnTiO_3_-NW/SG, and 20Mn-Ti-NW/SG), and one other catalyst from each group (20MnTiO_3_-NW/CS, 20MnTiO_3_-NW/SG, and 5Mn-Ti-NW/SG).

The XRD patterns of these catalysts are collected in Supplementary Figure S2, with the assignment of XRD peaks to crystalline phases summarized in Supplementary Table S1. All catalysts exhibited XRD peaks characteristic of Na_2_WO_4_ and α-cristobalite. This confirmed the transformation of amorphous SiO_2_ to α-cristobalite assisted by Na atoms, in line with the previous reports^20,23,45^. Nevertheless, crystalline Mn_2_O_3_, Mn_3_O_4,_ and TiO_2_ were also detected, indicating that the MnTiO_3_ particles partially decomposed into MnO_x_ and TiO_2_ during catalyst preparation. The crystalline Mn_2_O_3_, Mn_3_O_4,_ and TiO_2_ phases were observed for the catalysts prepared with Mn and Ti. The MnTiO_3_ phase was detected in all catalysts that were prepared with MnTiO_3_. Weak signals of the α-tridymite phase in all catalysts and strong signals of quartz were perceived in 10wt% and 20wt%MnTiO_3_-NW/SG. The catalytic tests (Table 1) indicate that the catalysts containing MnTiO_3_, MnO_x_, TiO_2_, and α-cristobalite had high C_2+_ yields; thus, these crystalline phases seem essential in enhancing C_2+_ formation.

**Figure 1:**
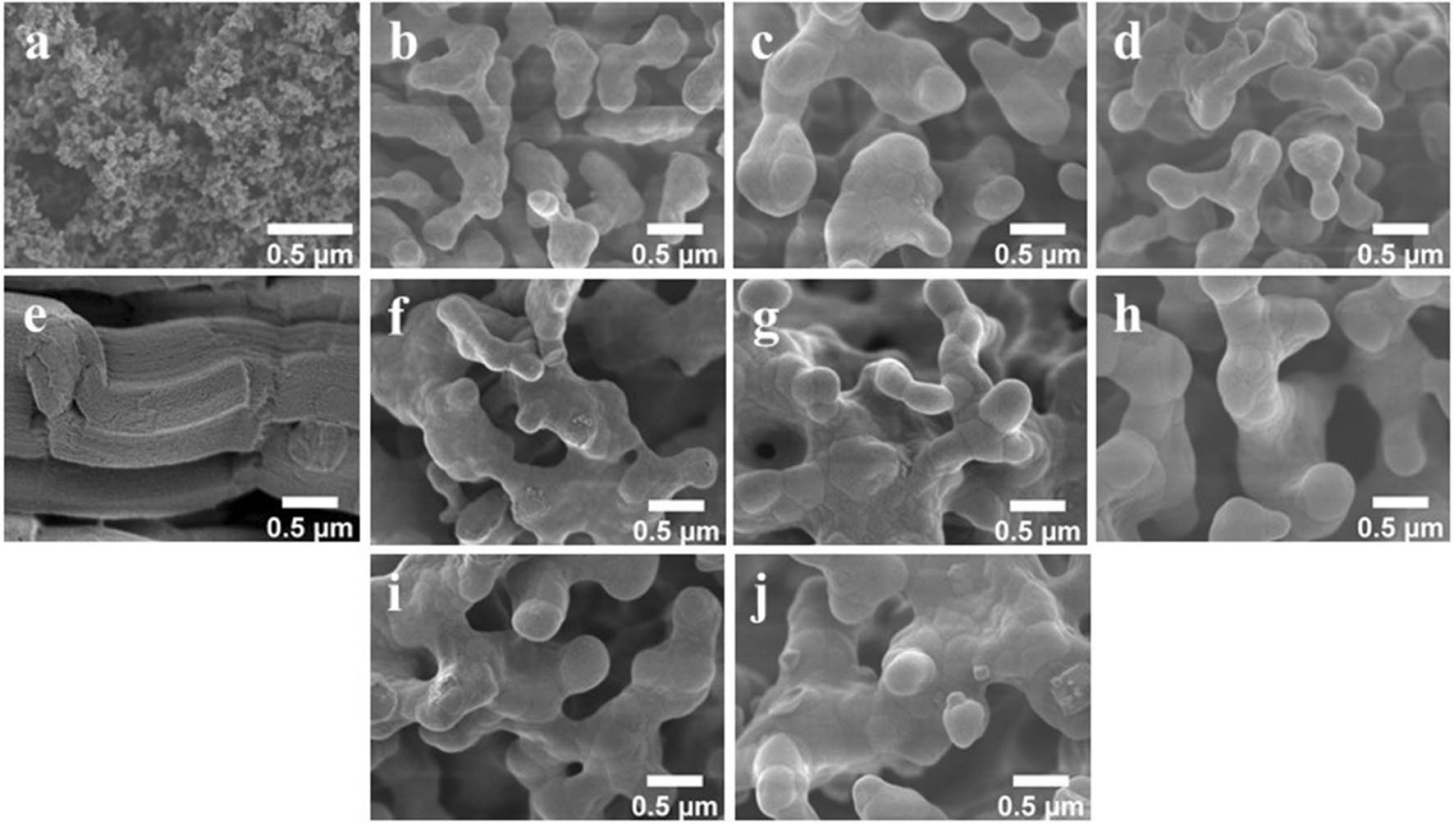
FE-SEM images of catalysts: (**a**) CS, (**b**) NW/CS, (**c**) 5MnTiO_3_-NW/CS, (**d**) 20MnTiO_3_-NW/CS, (**e**) SG, (**f**) NW/SG, (**g**) 10MnTiO_3_-NW/SG, (**h**) 20MnTiO_3_-NW/SG, (**i**) 5Mn-Ti-NW/SG, and (**j**) 20Mn-Ti-NW/SG.

**Figure 2:**
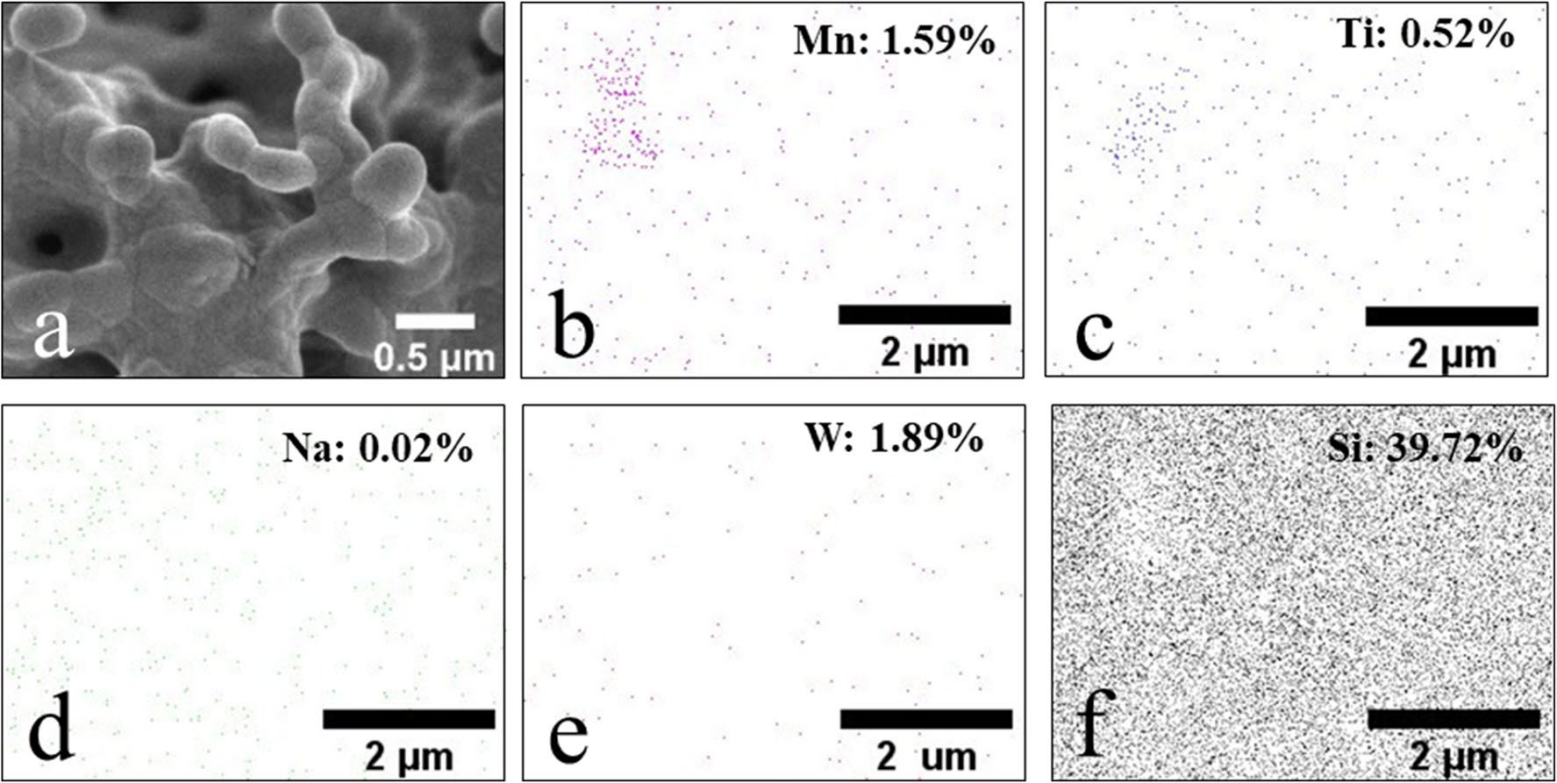
(**a**) FE-SEM and (**b**), (**c**), (**d**), (**e**), and (**f**) EDX images and weight percent each elemental of 10MnTiO_3_-NW/SG.

The morphology of the catalysts imaged by FE-SEM is presented in Fig. 1 and the average particle size of each catalyst determined using ImageJ software is presented in Supplementary Figure S3–S12 and Table S2–S11. The CS support appeared spherical with an average particle size of 34 nm (Fig. 1a), whereas SG contained multiple layers (average thinness of 0.4 µm) with porosity (Fig. 1e). All prepared catalysts had similar coral-like particles. However, the average width of particles of catalysts prepared from CS (approximately 0.3–0.5 µm) was somewhat smaller than those from SG (approximately 0.4–0.6 µm). Upon the addition of different quantities of MnTiO_3_ or Mn-Ti to the catalysts, the particle size and shape on the micrometer-scale changed negligibly. The elemental distribution and amount of Mn, Ti, Na, W, and Si in 10MnTiO_3_-NW/SG (Fig. 2) and the other selected catalysts (Supplementary Table S12) were examined by FE-SEM with EDX. Each element was well-dispersed over the surface of the catalysts, which facilitated the activation of CH_4_^10^.

**Figure 3:**
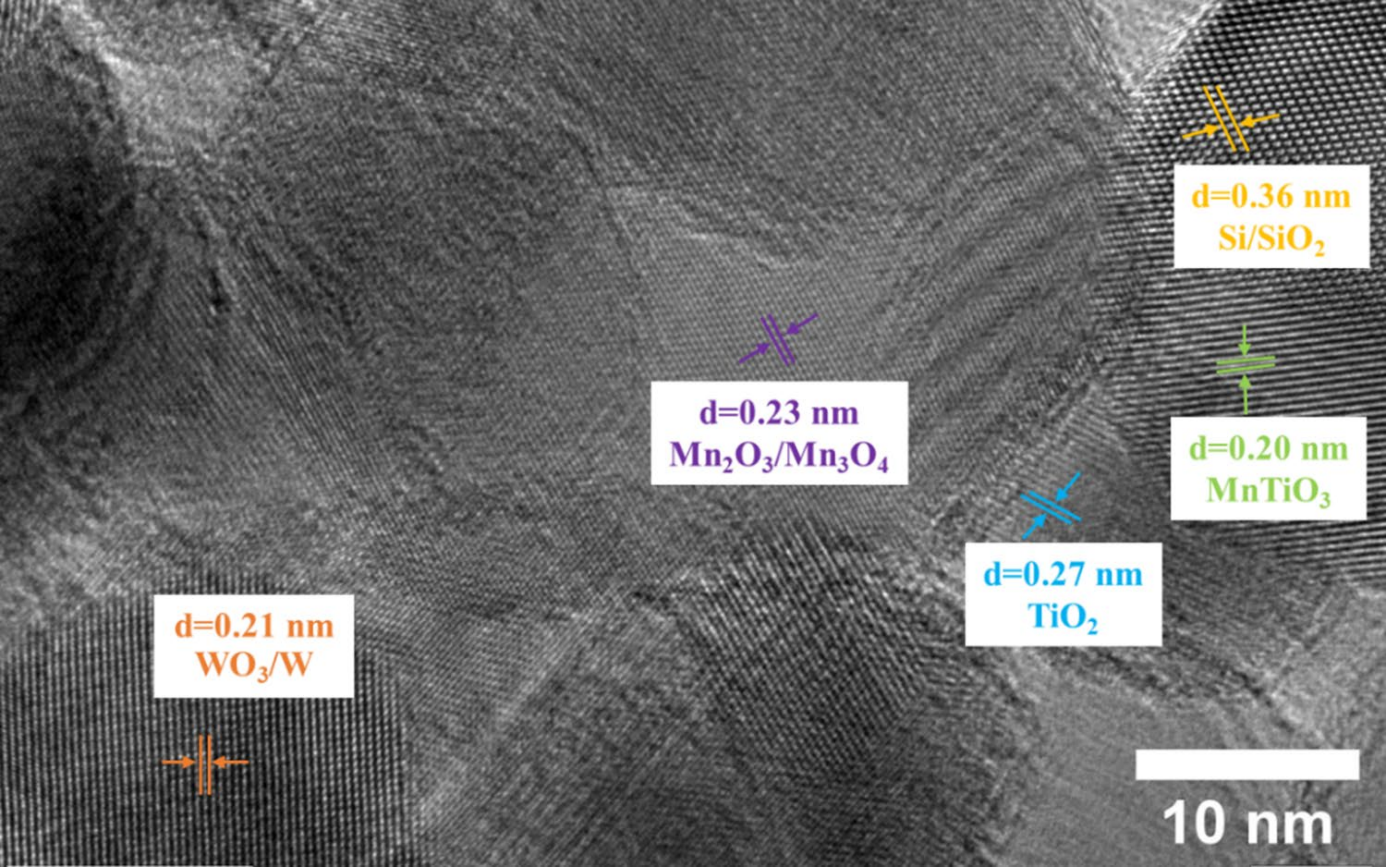
TEM image of 10MnTiO_3_-Na_2_WO_4_/SG.

The structural morphologies of 10MnTiO_3_-Na_2_WO_4_/SG were characterized using HR-TEM as shown in Fig. 3. As seen in Fig. 3, the active sites were distributed throughout the SG. The Si/SiO_2_ had a lattice d-spacing of 0.36 nm, corresponding to the Si crystal lattice [1 1 0]^46^. The lattice d-spacings of 0.20 and 0.27 nm corresponded to the crystal plane of MnTiO_3_ [1 1 0]^47^ and TiO_2_ [1 0 1]^47^, respectively. The WO_3_/W had a lattice d-spacing of 0.21 nm, relating to the lattice plane of WO_3_/W [2 0 1]^48^. The Mn_2_O_3_ and Mn_3_O_4_ had a lattice d-spacing of 0.23 nm, corresponding to the crystal plane of Mn_2_O_3_ [1 1 1]^49^ and Mn_3_O_4_ [0 1 0]^50^. The HR-TEM results confirm that the active phases distributed throughout the SG support and 10MnTiO_3_-Na_2_WO_4_/SG are nanocomposite due to the nano-scale structures^51^.

The BET surface area, pore-volume, and pore size of all catalysts determined by N_2_-physisorption are presented in Table 2 (isotherms are shown in Supplementary Figure S13). SG had the highest BET surface area (409.2 m^2^ g^-1^), followed by CS (89.6 m^2^ g^-1^). After using the supports for catalyst preparation, the BET surface areas were largely reduced (0.7–5.7 m^2^ g^-1^). The surface area of similar catalysts was also reduced after calcination (2.9–8.6 m^2^ g^-1^; amorphous SiO_2_ support 89.6 m^2^ g^-1^)^10,38^. The tremendous reduction of the surface area of the supports (i.e., SG and CS) generally occurs when preparing a catalyst containing Na_2_WO_4_/SiO_2_. This is because the structure of the support (e.g., porous material and/or nanoparticle) has completely changed from nanoparticles (30–50 nm) to large grain sizes (approximately 0.2–1.0 µm) as indicated by the SEM images in Fig. 1 of an α- cristobalite-containing catalyst without internal pores (i.e., non-porous material)^27,52^. Thus, a very small surface area (e.g., < 6 m^2^/g) is obtained. The catalysts containing MnTiO_3_ exhibited smaller surface areas, pore-volumes, and pore sizes than those without MnTiO_3_. Similarly, the addition of Mn + Ti produced catalysts with a small surface area (3.2–3.4 m^2^ g^-1^), but still larger than those containing MnTiO_3_. Nevertheless, there was a negligible difference between the pore volumes and pore sizes of catalysts with and without Mn + Ti. Moreover, the increased loading of MnTiO_3_ from 10 to 20 wt% on NW/SG or Mn + Ti from 5 to 20 wt% on NW/SG did not significantly change the surface area. Based on the N_2_-physisorption isotherm analysis in Supplementary Figure S13, and according to the IUPAC classification, the SG support is specified as type IV (mesoporous material, containing pore size of 5–10 nm) with H1 hysteresis, while the CS support and all the prepared catalysts showed an indistinct hysteresis loop. However, after careful analysis of the plots shown as inserts of Supplementary Figure S13 and the SEM images (Fig. 1), the CS support and the catalysts appeared to be non-porous with rough surfaces, which would be classified as type II (nonporous material).

**Table 2:** BET surface area, pore volume, and pore size of catalysts.

Catalyst	BET surface area (m^2^ g^-1^)	Pore volume (cm^3^ g^-1^)	Pore size (nm)
CS	89.6	-	-
NW/CS	5.7	-	-
5MnTiO_3_-NW/CS	3.2	-	-
20MnTiO_3_-NW/CS	0.7	-	-
SG	409.2	0.545	5–10
NW/SG	3.8	-	-
10MnTiO_3_-NW/SG	1.7	-	-
20MnTiO_3_-NW/SG	1.8	-	-
5Mn-Ti-NW/SG	3.2	-	-
20Mn-Ti-NW/SG	3.4	-	-

**Figure 4:**
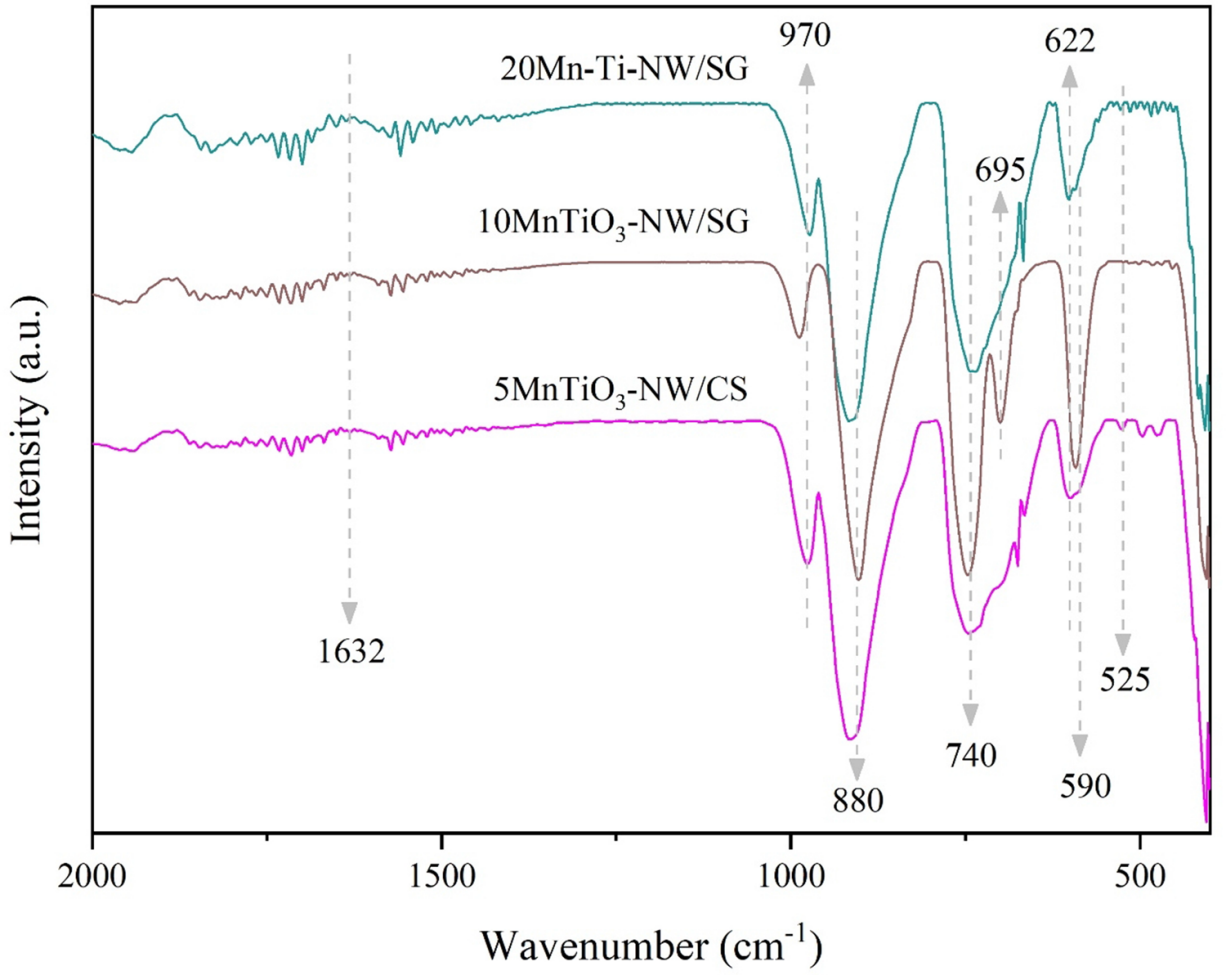
FTIR spectra of selected catalysts.

The FTIR patterns of selected catalysts producing the maximum yield in each group (5MnTiO_3_-NW/CS, 10MnTiO_3_-NW/SG, and 20Mn-Ti-NW/SG) are displayed in Fig. 4. The FTIR peaks at 970, 880, and 740 cm^-1^ correspond to Si–O–(H–H_2_O)^28^, Si–OH^53^, and Si–O–C^54,55^ bonds, respectively. The FTIR peak at 622 cm^-1^ for all catalysts characterizes α-cristobalite SiO_2_^45,56^. Previous studies suggested that the amorphous SiO_2_ can transform to the α-cristobalite SiO_2_ at 800 °C if Na is present during the calcination^20,21^. To confirm that, CS, Na/CS, and NW/CS were prepared using the same procedure as 5MnTiO_3_-NW/CS and analyzed using FTIR as shown in Supplementary Figure S14. The peak at 622 cm^-1^ that specifies the α-cristobalite SiO_2_ phase was also observed when Na was present (i.e., Na/CS and NW/CS). This is in good agreement with the reports^21^. An FTIR peak at 695 cm^-1^ was only observed for 10MnTiO_3_-NW/SG, indicating the presence of quartz (SiO_2_)^56^. The FTIR peak at 525 cm^−1^ is associated with the bending vibration mode of O–Mn–O^57^, indicating the presence of MnO_2_. The intense peak at 590 cm^−1^ originates from the vibration of the O–Ti–O bond^58^, which was detected in the TiO_2_-containing catalyst. The peak at 1632 cm^−1^ was assigned to the O–H bending mode, due to moisture^59^.

The surface composition of the catalysts was analyzed by XPS (Supplementary Table S13 and Supplementary Figure S15–S19). The curve-fitted and quantified XPS peaks of Na 1s, W 4f., Si 2p, Ti 2p, and Mn 2p are presented in Supplementary Table S13. The XPS peaks were considered as a mixture of Gaussian and Lorentzian functions (80:20 ratio)^60^. Na is a component of Na_2_WO_4_ (Supplementary Figure S15). The binding energies of W 4f_7/2_ and W 4f_5/2_ (Supplementary Figure S16) in every catalyst indicated the presence of WO_4_^2,23^. The peaks of Si 2p (Supplementary Figure S17) correspond to SiO_2_. The binding energies of Mn 2p (Supplementary Figure S18) and Ti 2p (Supplementary Figure S19) for nanocomposite MnTiO_3_-NW/CS and MnTiO_3_-NW/SG were different from those of nanocomposite Mn-Ti-NW/SG.

**Figure 5:**
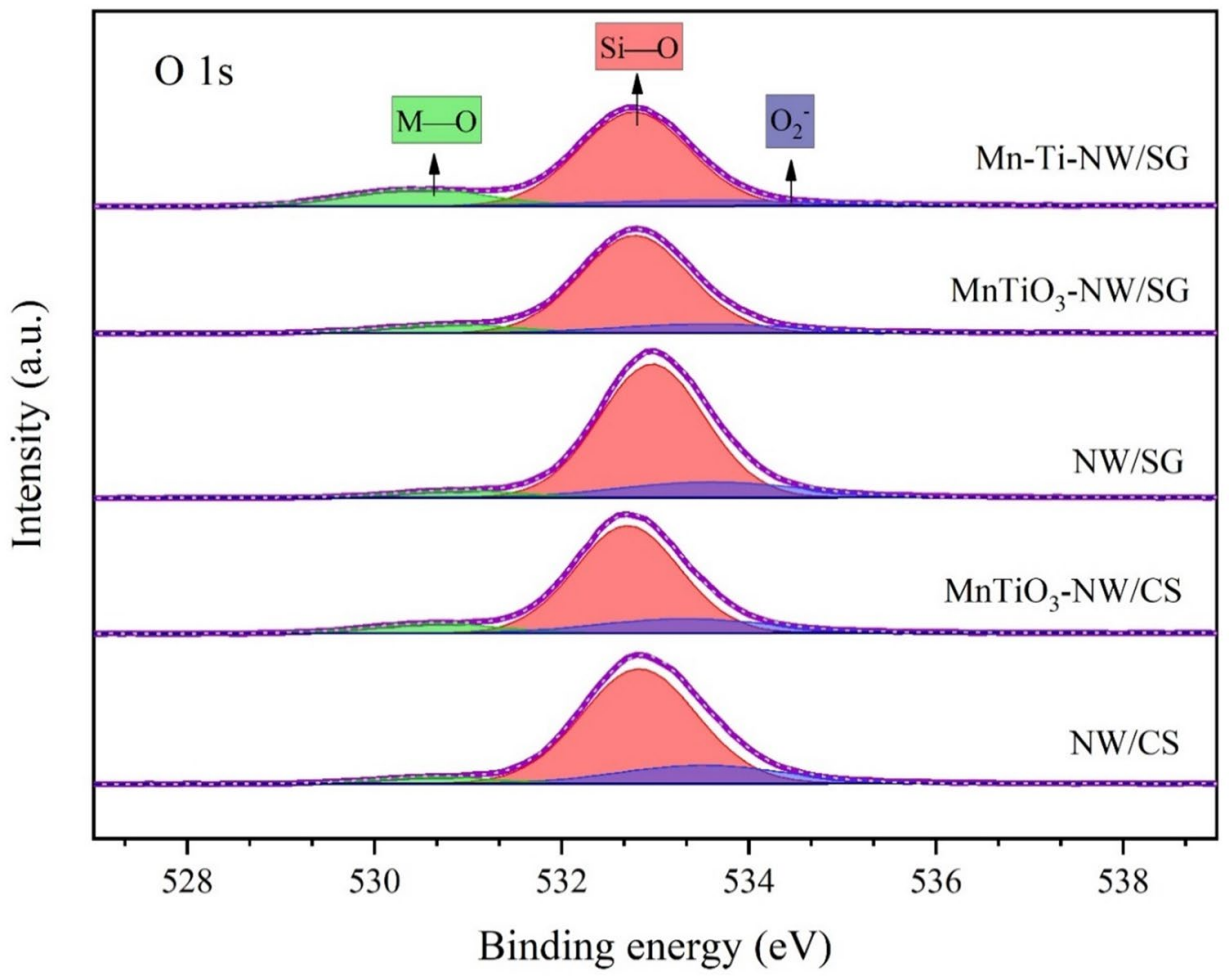
XPS spectra in the O 1 s region for different catalysts.

In the OCM mechanism, the oxygen species are crucial for CH_4_ activation. The XPS spectra and the O 1 s binding energies of the different catalysts are presented in Fig. 5 and Table 3, respectively. Overall, three types of oxygen species were identified: oxygen of M–O species at 530.5–530.9 eV, oxygen of Si–O at 532.7–532.9^61,62^, and O_2_^-^ at 533.3–533.6 eV^63^. M–O represents the oxygen species of Na–O at 530.8 eV^64^, W–O at 530.6 eV^64^, and Mn–O at 530.3 eV^65^, and Ti–O showing two peaks at 530.0 and 531.8 eV^64^. The peaks around 530.5–530.9 eV for M–O in NW/CS and NW/SG were relatively small (as NW/CS and NW/SG had no components of Mn–O and Ti–O), while relatively large peaks were observed for MnTiO_3_-NW/CS, MnTiO_3_-NW/SG, and Mn-Ti-NW/SG. The O^-^, and O_2_^-^ lattice species are essential for CH_4_ activation^66,67^, whereas the O^2-^ lattice species leads to complete oxidation of hydrocarbon products to CO_x_^60^. Based on the activity results in Fig. 5, catalysts containing M–O (i.e., XPS peak around 530.5–530.9 eV) exhibited high CH_4_ conversion. This implied that a catalyst with this specific oxygen species promotes CH_4_ activation as the lattice oxygen of the catalyst surface is easily reacted and re-populated (high oxygen mobility).

**Table 3:** Curve-fitted and quantified O 1 s XPS results of catalysts.

Catalyst	Binding energy (eV)		
	O1s, (FWHM), and relative amount (%)		
	^a^ M–O	Si–O	O_2_^-^
NW/CS	530.6 (1.5) 4.9	532.8 (1.4) 77.4	533.5 (2.1) 17.6
MnTiO_3_-NW/CS	530.6 (1.6) 8.7	532.7 (1.3) 74.9	533.3 (2.1) 16.4
NW/SG	530.9 (1.5) 5.1	532.9 (1.3) 79.3	533.6 (2.3) 15.7
MnTiO_3_-NW/SG	530.8 (1.7) 8.8	532.8 (1.4) 79.1	533.5 (2.3) 12.0
Mn-Ti-NW/SG	530.5 (1.8) 17.4	532.8 (1.4) 72.7	533.6 (3.1) 9.9

^a^ M–O (M = Na, W, Mn, or Ti): FWHM = Full width at half maximum.

### Stability of catalysts in the OCM reaction

**Figure 6:**
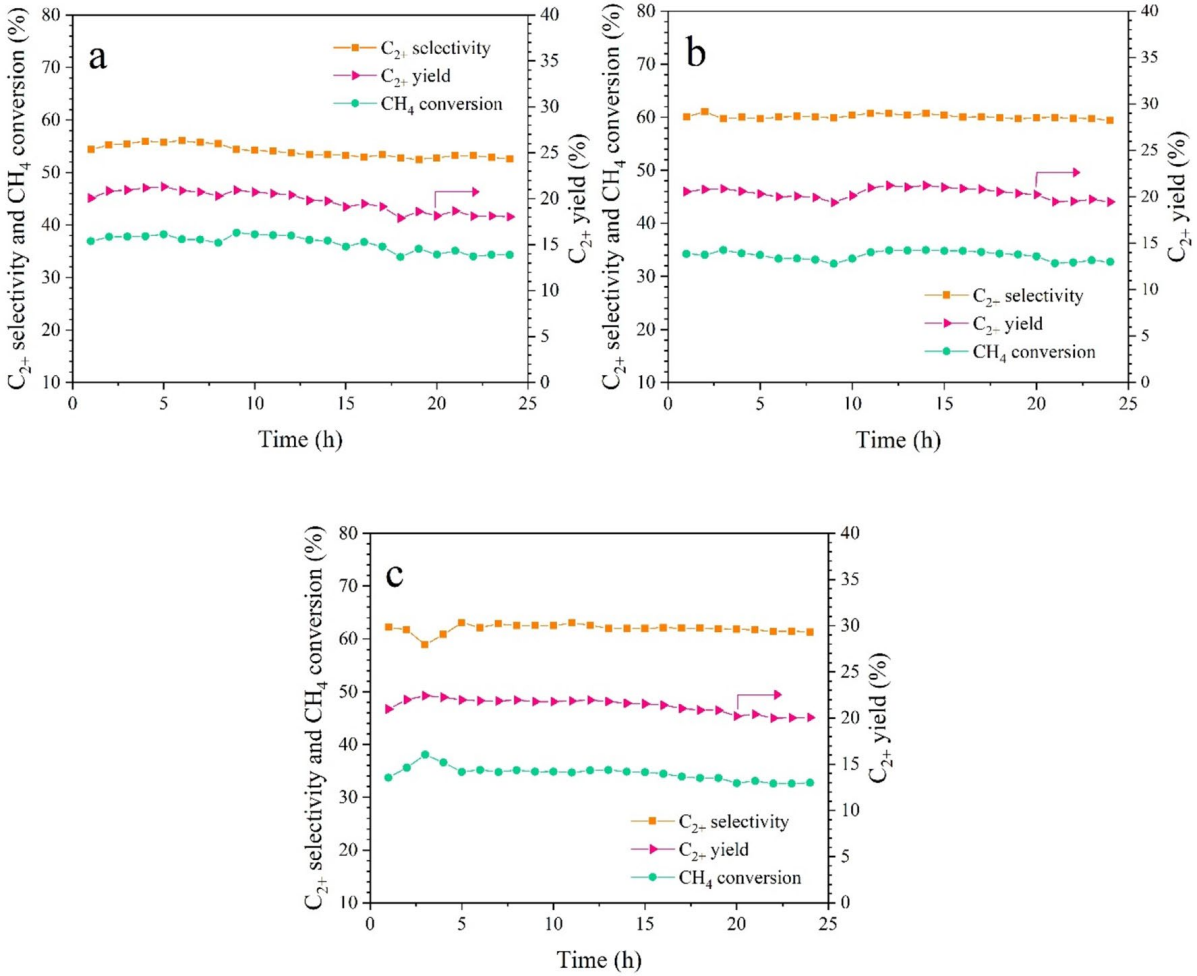
Catalytic performance in OCM reaction: (**a**) MnTiO_3_-NW/CS, (**b**) MnTiO_3_-NW/SG, and (**c**) Mn-Ti-NW/SG catalysts over 24 h. Conditions: 50 mg catalyst, gas feed of CH_4_:O_2_:N_2_ = 3:1:4, reactor temperature = 700 °C, atmospheric pressure, total feed gas flow rate = 50 mL min^-1^ (GHSV = 30,588 h^-1^).

The stability of 10MnTiO_3_-NW/CS, 5MnTiO_3_-NW/SG, and 20Mn-Ti-NW/SG, catalysts that had produced the highest C_2+_ yields, was further tested for over 24 h (Fig. 6). The three catalysts had similar performance with the maximum C_2+_ yield (~ 21–23%) obtained within 3–4 h. After that, performance slightly diminished, especially for CH_4_ conversion and C_2+_ yield. The reduction levels in terms of C_2+_ yield for MnTiO_3_-NW/CS (Fig. 6a), MnTiO_3_-NW/SG (Fig. 6b), and Mn-Ti-NW/SG (Fig. 6c) were 15.3%, 8.4%, and 10.5%, respectively. Moreover, 20wt% Mn-Ti-NW on CS (20Mn-Ti-NW/CS) was prepared and tested for reaction to compare its performance with that of 20Mn-Ti-NW/SG (see Supplementary Figure S20). As observed, the activity of Mn-Ti-NW/CS decreased faster than the other catalysts, similar to the previous reports^27,37–39^. This confirms that the MnTiO_3_-NW/SG catalyst was the most active and durable catalyst among all prepared.

**Figure 7:**
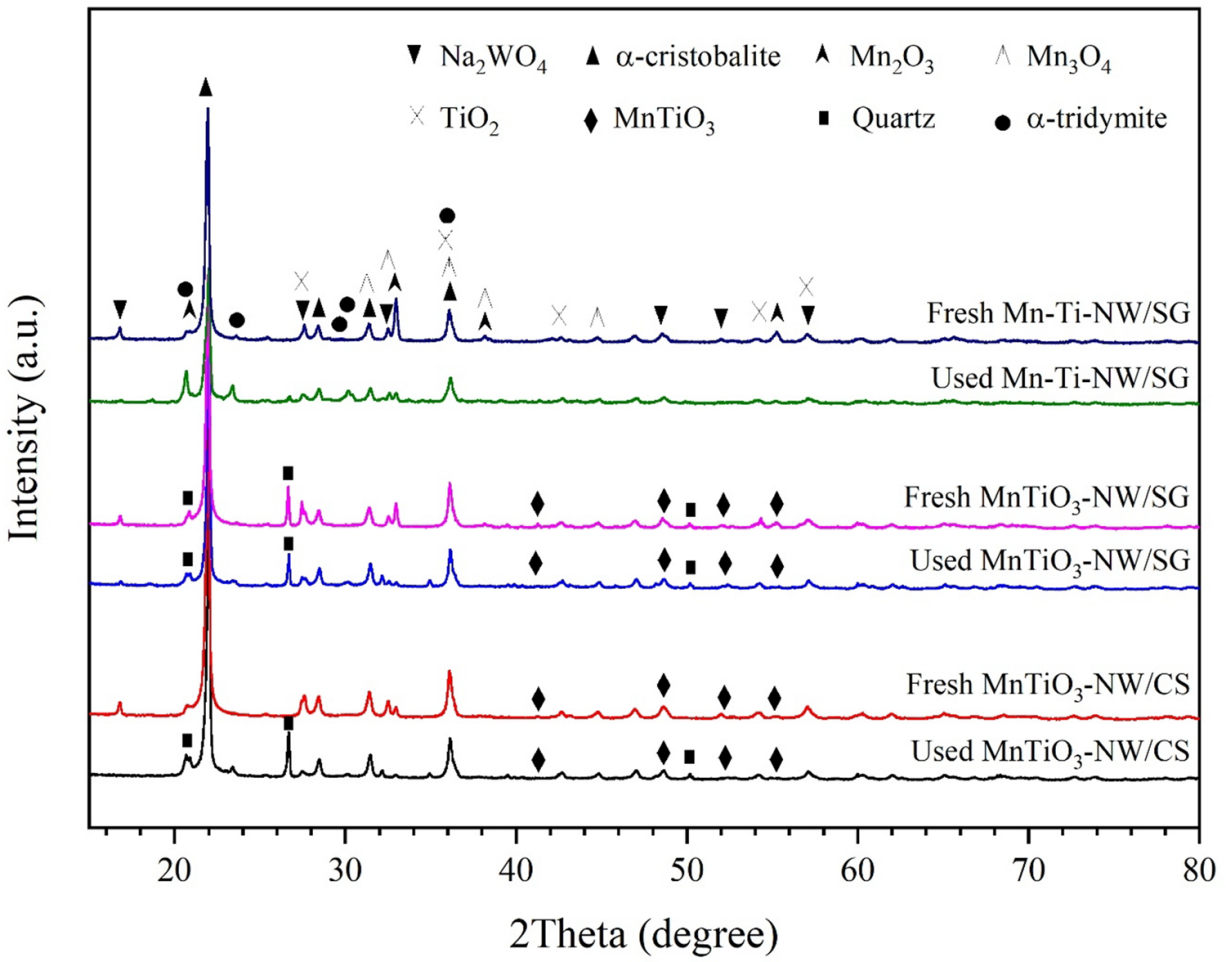
XRD patterns of fresh and used (24 h) catalysts.

The XRD patterns of fresh and used catalysts are presented in Fig. 7 (for peaks assignments see Supplementary Table S1). Some XRD peaks indicated changes. First, the Na_2_WO_4_ peaks in used MnTiO_3_-NW/CS and Mn-Ti-NW/SG catalysts had disappeared, which could be a result of the destruction of crystalline Na_2_WO_4_^11,68,69^. This may be related to the reduction in catalytic activity. Second, the quartz phase was found in the used MnTiO_3_-NW/CS catalyst and the presence of α-tridymite became more pronounced in the used Mn-Ti-NW/SG. Thus, the activity of the catalysts was reduced, because these two phases do not promote methane activation. For the MnTiO_3_-NW/SG catalyst, the XRD peaks after reaction remained practically unaffected, confirming the excellent catalytic stability of MnTiO_3_-NW/SG with no activity loss as changes in crystalline phases were absent.

**Figure 8:**
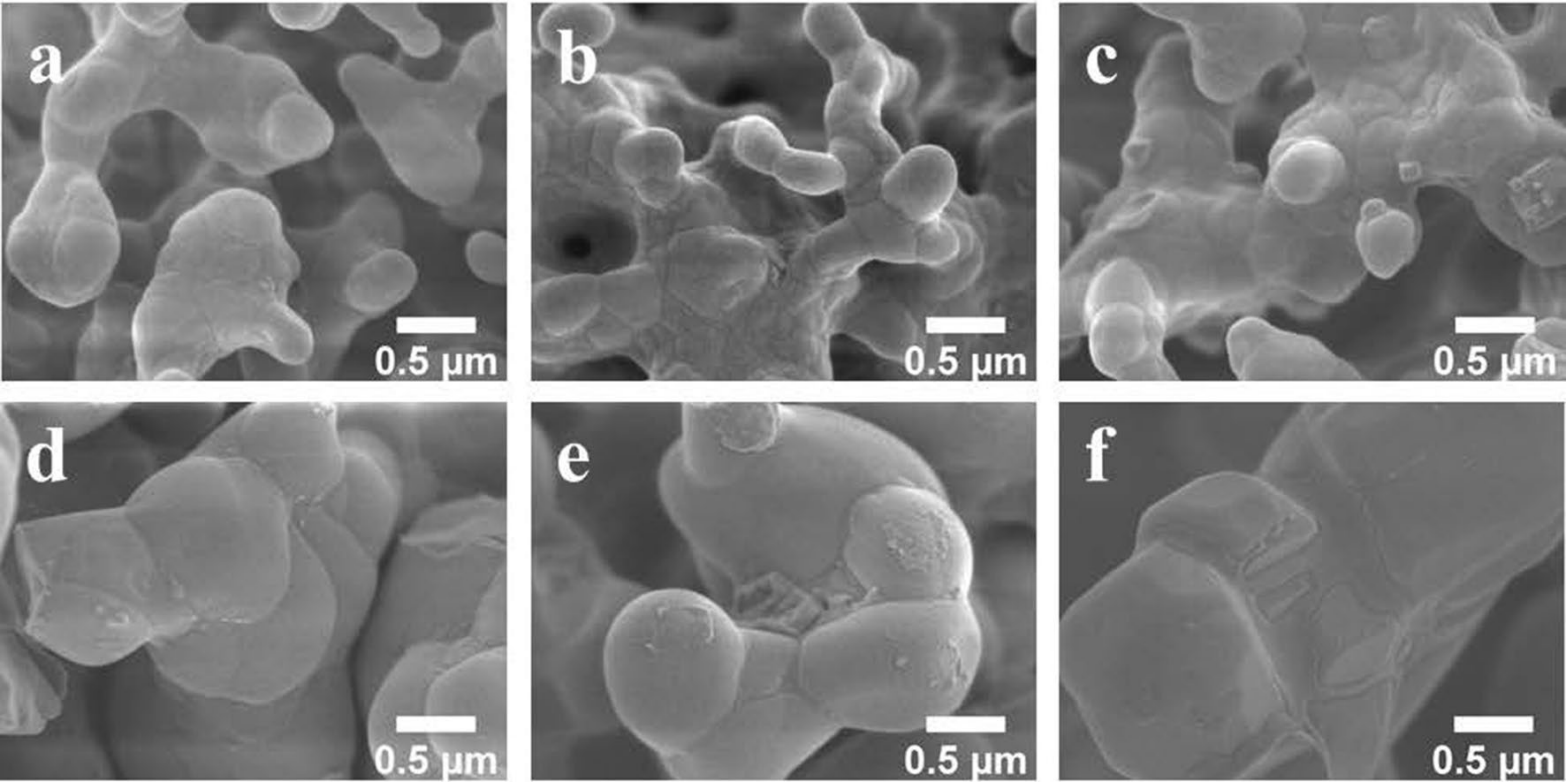
FE-SEM images of fresh and used catalysts: (**a**) Fresh MnTiO_3_-NW/CS, (**b**) Fresh MnTiO_3_-NW/SG, (**c**) Fresh Mn-Ti-NW/SG, (**d**) Used MnTiO_3_-NW/CS, (**e**) Used MnTiO_3_-NW/SG, and (**f**) Used Mn-Ti-NW/SG.

The morphologies of fresh and used catalysts once more analyzed by FE-SEM (Fig. 8) and the average particle size of used catalysts were determined using ImageJ software and is presented in Supplementary Figure S21–S23 and Table S14–S16. The metal distribution of each catalyst was also characterized by FE-SEM with EDX (Supplementary Table S12). When the FE-SEM images of fresh and used (24 h) catalysts were compared, the particle shapes appeared very similar. Nevertheless, the particle size of each used catalyst (approximately 1.2–1.6 µm) was more than double of each fresh catalyst (approximately 0.3–0.6 µm). This is paralleled by a reduction in surface area, so that performance in OCM decreased after testing for several hours. Moreover, the increase in particle size may also result in a partial loss of the catalytic interface between the active components for activating methane (i.e., Na_2_WO_4_ and Mn_2_O_3_)^68,69^, thereby causing decreased catalytic activity. The EDX images of the used catalysts (Supplementary Table S12) indicated the presence of Mn, Ti, Na, and W elementals on the catalyst surface. The distribution and concentration of the active elements did not significantly change, in good accordance with the stability tests.

Another reason why the catalysts prepared using SG were more stable than those using CS is presented in Scheme 1. According to the textural properties in Table 2, SG has a larger surface area with high porosity, while CS has no internal pores. Thus, during impregnation, the active components were impregnated inside the pores of SG before the transformation of amorphous SiO_2_ to α-cristobalite, so that the active components were widely dispersed inside the catalyst’s pores. In contrast, the active components impregnated on CS were dispersed only over the surface of CS (like a thin film coating^69^), since it is non-porous (see CS in Scheme 1). According to some reports, the crystalline phase of Na_2_WO_4_ disappeared due to slow evaporation within several hours of the stability test^11,68,69^ and/or it transforms to another phase (MnWO_4_)^68^. Notably, the melting temperature of Na_2_WO_4_ is 698 °C, while the reaction temperature for OCM is over 700 °C due to exothermicity^11^. Therefore, it seems easier to lose Na_2_WO_4_ from the CS-supported than from the SG-supported catalyst.

**Figure 9:**
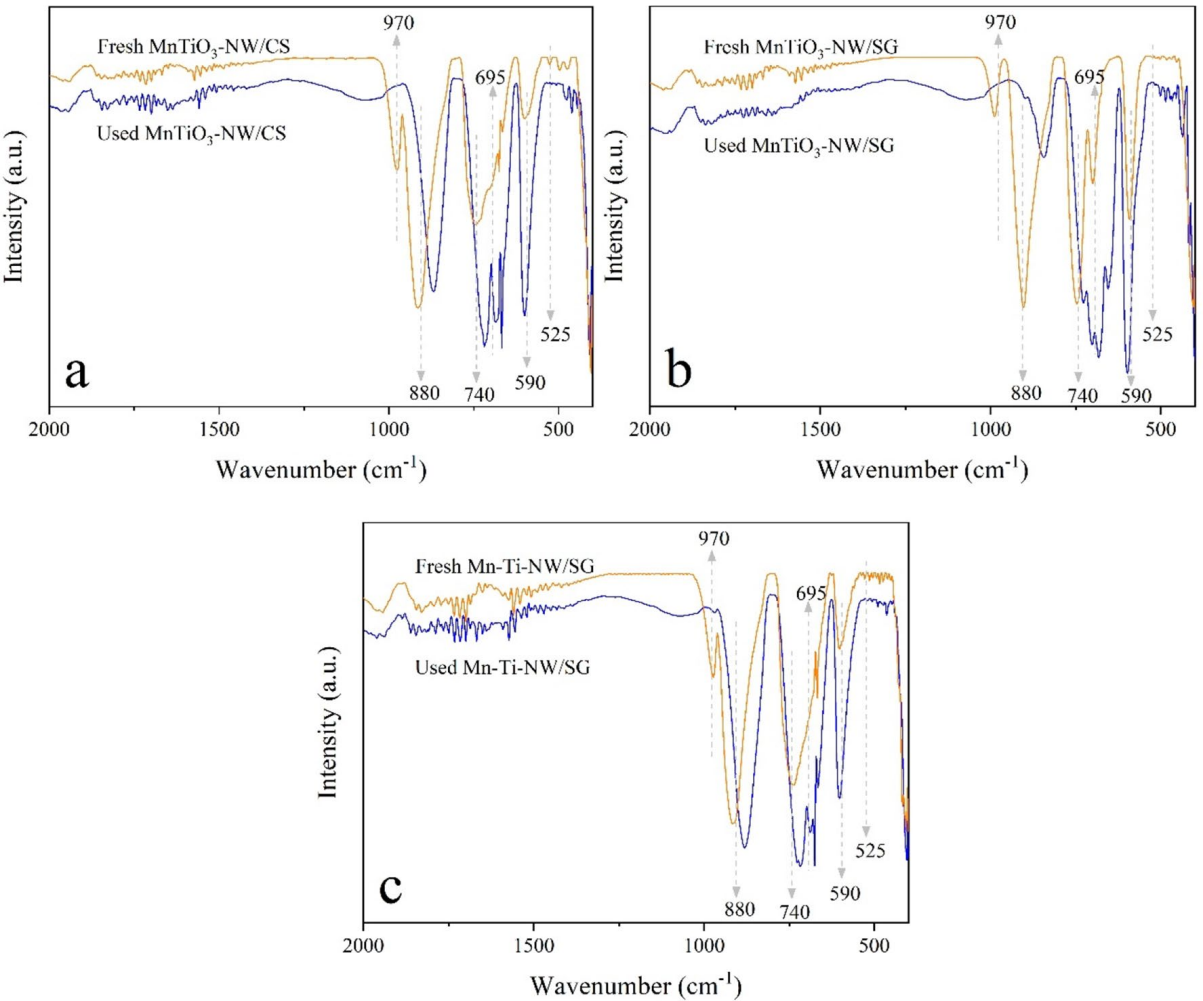
FTIR spectra of fresh and used catalysts: (**a**) 5MnTiO_3_-NW/CS, (**b**) 10MnTiO_3_-NW/SG, and (**c**) 20Mn-Ti-NW/SG.

The FTIR spectra of fresh and used catalysts are shown in Fig. 9. After the catalysts had been used, the FTIR peak of Si–O–(H–H_2_O) disappeared, suggesting that the H_2_O attached to Si–O–H was removed. Additionally, there was an FTIR peak appearing at 695 cm^-1^, indicating the presence of quartz (SiO_2_)^56^. The quartz phase was detected in the fresh MnTiO_3_-NW/SG and was clearly visible in all used catalysts, which is in good agreement with the XRD results in Fig. 7. This suggested that the α-cristobalite phase slowly transformed into the quartz phase. The FTIR peak at 524 cm^−1^ was assigned to the bending vibration of O–Mn–O^57^ and it was detected in each catalyst. The intense peak at 590 cm^−1^ was the vibration of the O–Ti–O bond^58^, which was also perceived in each catalyst.

**Scheme 2:**
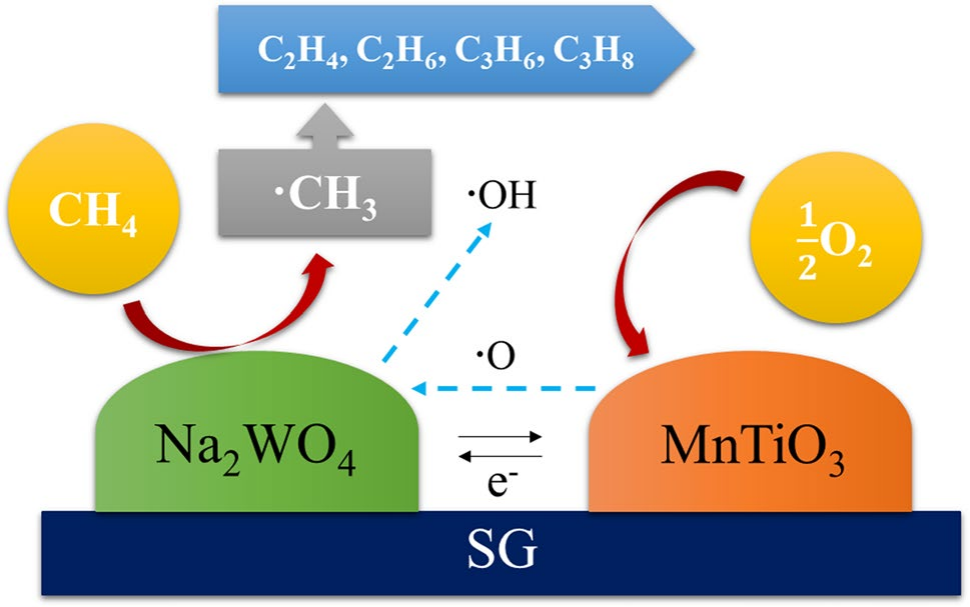
Mechanism of OCM over MnTiO_3_-NW/SG.

The plausible mechanism of this reaction over MnTiO_3_-NW/SG is illustrated in Scheme 2. The most possible active site for the CH_4_ activation is the lattice oxygens of WO_4_^2,30^. During the CH_4_ activation to CH_3_ radical by the lattice oxygens, W^6+^ is reduced to W^5+^ ^30^. The CH_3_ radical is then coupled with another CH_3_ radical to yield C_2_H_6_ in the gas phase, which can further generate C_2_H_4_ and other hydrocarbons via the C–H activation and dehydrogenation over the catalyst surface. However, the oxidation of CH_4_ can occur at the same temperature (600–1,000 ºC) to produce CO and CO_2_. A sketch of the mechanisms and series of OCM reaction, CH_4_ oxidation, and dehydrogenation are presented in Supplementary Information equations (S1)–(S14)^19^. At the same time, due to the facile mobility of the lattice oxygen of Mn–O species^27^, the lattice oxygen from MnTiO_3_ is transferred to the WO_4_^2-^ species. As a result, Mn^2+^ is oxidized to Mn^3+^. After that, the O_2_ molecule from the gas phase is adsorbed onto the surface of MnTiO_3_ and the OH radical is released from the WO_4_^–2^ surface. An electron exchange from W^5+^ to Mn^3+^ simultaneously occurs, which regenerates W^6+^ and Mn^2+^, and is ready for the new cycle of the reaction.

**Figure 10:**
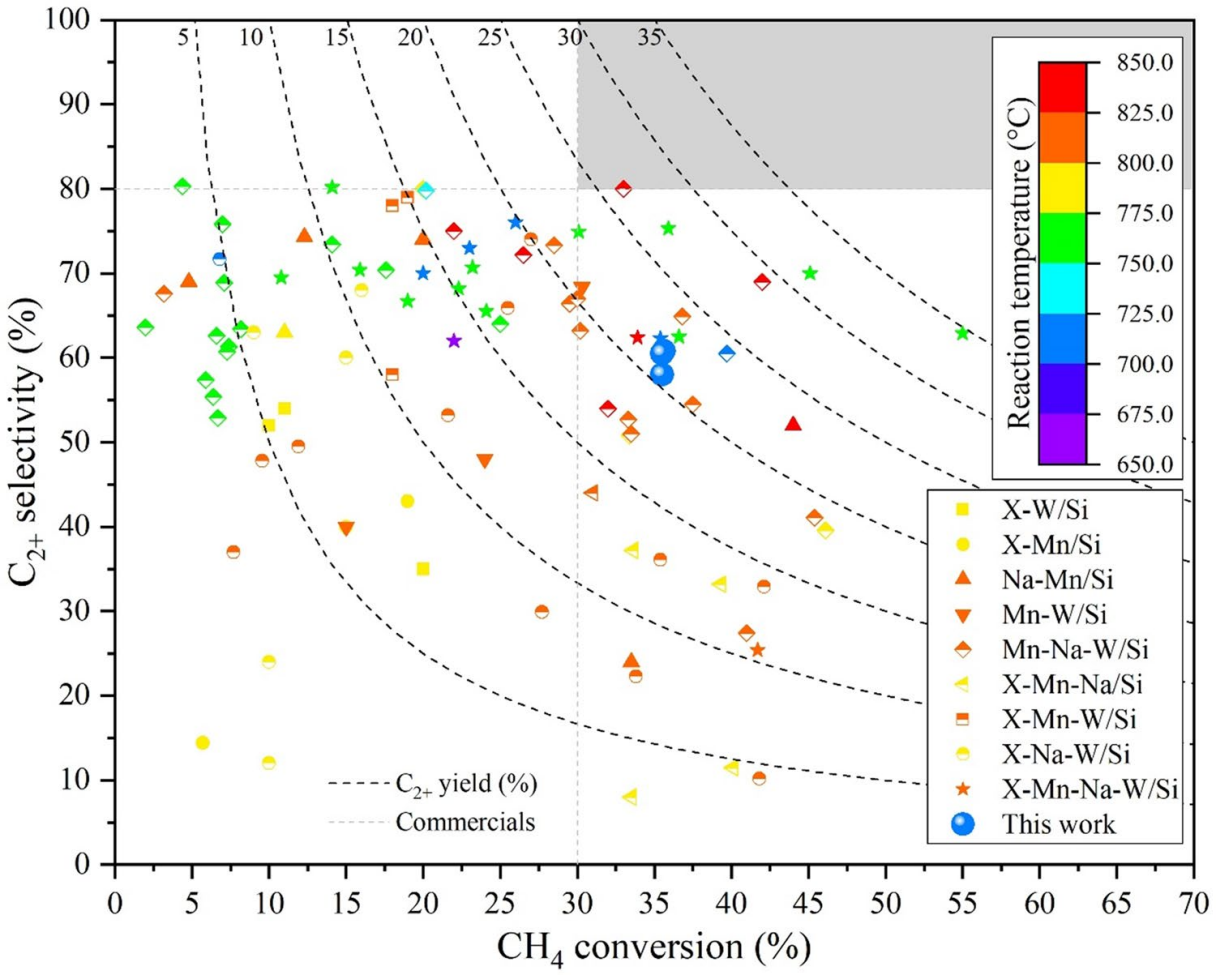
Survey of various catalysts reported in the literature, including the current ones. The gray area in the top right-hand corner indicates the target zone for commercially viable catalyst performance.

A survey of various catalysts reported in the literature for the OCM reaction is presented in Fig. 10, with the details of each catalyst described in Supplementary Table S17. The activity of OCM catalysts should be over 30% CH_4_ conversion and 80% C_2+_ selectivity^7^ to have the potential for commercial exploitation (indicated by the gray area in Fig. 10). The catalysts studied herein are outside the required area; specifically, as their C_2+_ selectivity is below 80%. Several reported catalysts also had a CH_4_ conversion above 30% with a C_2+_ selectivity below 80%, however, all of them were used at a temperature above 750 °C. Thus, in light of the lower temperature (700 °C), our present catalysts exhibited good performance. Nevertheless, further improvement is necessary, especially increasing C_2+_ selectivity above 80% while maintaining the CH_4_ conversion. Some Mn-Na-W/Si and X-Na-W/Si catalysts have a C_2+_ selectivity of around 80% at a reaction temperature of 725–775 °C, but they have low CH_4_ conversions (4.4–20.2%)^15–17,31^. No catalyst based on Na, W, and/or Mn can provide a C_2+_ selectivity above 81%, implying that improving the C_2+_ selectivity while maintaining the CH_4_ conversion above 30% is very challenging. Remarkably, one report in 1998 showed that an Mn-Na-W/Si catalyst performed well in the OCM reaction, producing C_2+_ selectivity of 80% and CH_4_ conversion of 33% (for a yield of 26.4%) at a reaction temperature of 850°C^20^. Nevertheless, thereafter such high performance of the same catalyst was not reported. The addition of additives to Mn-Na-W/Si (as X-Mn-Na-W/Si) can improve the C_2+_ selectivity to about 62–75% with a CH_4_ conversion above 30%. When X = NaCl, the highest C_2+_ yield reported was 34.6% (62.9% C_2+_ selectivity and 55% CH_4_ conversion)^17^, but the catalysts were not stable for long periods due to a loss of chloride. The performance of our catalysts containing Mn, Na, and W components upon using SiO_2_ as catalyst support has been improved, identifying several key factors, but a viable catalyst for industrial use is still at large.

## Conclusion

Different loadings of nanocomposite MnTiO_3_-NW or MnO_x_-TiO_2_-NW supported on silica (CS and SG) were successfully prepared and tested in the OCM reaction. The highest C_2+_ yields were obtained for 10MnTiO_3_-NW/SG (21.6%), followed by 20Mn-Ti-NW/SG (21.5%), and 5MnTiO_3_-NW/CS (20.6%). These catalysts produced high levels of dehydrogenation, generating olefins/paraffin ratios of 2.1–2.3. Catalysts characterization by XRD detected MnTiO_3_, MnO_x_, TiO_2_, and α-cristobalite phases, while using XPS identified oxygen species of M–O (M = Na, W, Mn, and Ti) that were strongly related to high CH_4_ conversion and high C_2+_ yield. Considering the catalytic activity between MnTiO_3_-NW and Mn-Ti-NW on the same support (i.e., SG), a small activity difference was observed between these two when the metal loading at 5wt%, in which the activity of 5MnTiO_3_-NW/SG was lower than that of 5Mn-Ti-NW/SG, while they perceived no substantial difference in the activity at the higher loadings. Comparing the type SiO_2_ support, the activity results presented that the catalysts with porous SG support were more stable than those with non-porous CS as support. The gradual catalyst deactivation observed during the stability test, especially for the catalyst with CS, was mainly due to the destruction of crystalline Na_2_WO_4_. Therefore, further improvement of catalytic performance seems to require an alternative active component that is retained under harsh operating conditions.

## Methods

### Catalysts preparation

#### Preparation of MnTiO_3_ nanocatalysts

The MnTiO_3_ catalyst was prepared using the stearic acid gel method^70^. In the first step, stearic acid (0.4 mol, C_18_H_36_O_2_, 98%, PanReac AppliChem) was heated in a beaker at 90 °C until it melted. After that, manganese acetate (0.1 mol, Mn(OOCCH_3_)_2_.4H_2_O, Mn 22%, Alfa Aesar) was added dropwise. The mixture was continuously stirred for 8 h to form a dark brown solution. Then, tetra butyl titanate (0.1 mol, Ti[O(CH_2_)_3_CH_3_]_4_, ≥ 97.0%, Sigma-Aldrich) was added dropwise to the mixture upon continuous stirring for 1 h to form a homogeneous brown liquid. Next, the mixture was dried in an oven at 100 °C for 12 h. Finally, the dried gel was calcined in four stages in the air (KJ-M1200-27L, Kejia furnace). In the first stage, the dried gel was heated to 400 °C with a heating rate of 1 °C min^-1^. Second, the temperature was held at 400 °C for 40 min. Third, the temperature was ramped up to 800 °C with a heating rate of 3 °C min^-1^. Finally, the temperature was held at 800 °C for 2 h and then slowly cooled down to room temperature^70^. The crystalline MnTiO_3_ sample was ground to obtain a fine powder.

#### Preparation of the sol–gel SiO_2_ support

Pluronic P123 (0.0005 mol, with an average molecular weight of ~ 5800, Aldrich) was dissolved in DI water (101 mL) with concentrated HCl (6.72 mL, 37%, Rankem). The mixture was continuously stirred at 40 °C until a clear solution was obtained. Then, tetraethyl orthosilicate (TEOS; 0.03 mol, (C_2_H_5_O)_4_Si, ≥ 99.0%, Aldrich) was added to the mixture followed by continuous stirring for 4 h at 40 °C. After that, the mixture was dried overnight in a hot-air oven at 100 °C. Next, the dried sample was washed with DI water (150 mL) and dried again in a hot-air oven at 100 °C for 12 h. Finally, the dried sample was calcined in air at 550 °C for 3 h with a heating rate of 3 °C min^-1^. The calcined sample was used as the sol–gel SiO_2_ support.

#### Preparation of MnTiO_3_-Na_2_WO_4_ nanocomposites on silica

The silica-supported MnTiO_3_-Na_2_WO_4_ catalyst was prepared using the impregnation method. The synthesized sol–gel SiO_2_ support and a commercial fumed SiO_2_ (c-SiO_2_, amorphous fumed, a specific surface area of 85–115 m^2^ g^-1^, Alfa Asesar) were used. Initially, sodium tungsten hydrate (Na_2_WO_4_.2H_2_O, 98.0–101.0%, Daejung) was dissolved in DI water to have a concentration of 0.05 M. The prepared catalysts had loadings of 5.0 wt% of Na_2_WO_4_ and 0.0–20.0 wt% MnTiO_3_ for each support. Accordingly, different amounts of Na_2_WO_4_.H_2_O solution and fine MnTiO_3_ powder were loaded on each support. After mixing, the solution was continuously stirred at room temperature for 1 h. Then, the mixture was dried in hot-air at 100 °C for 1 h. The dried mixture was finally calcined in the air furnace at 800 °C for 4 h with a heating rate of 2 °C min^-1^. The obtained catalysts were MnTiO_3_-Na_2_WO_4_/c-SiO_2_ (denoted as MnTiO_3_-NW/CS) and MnTiO_3_-Na_2_WO_4_/sol–gel SiO_2_ (denoted as MnTiO_3_-NW/SG). A commercial fumed silica with a surface area of 350–420 m^2^/g was purchased and used to prepare a parallel catalyst with 5MnTiO_3_-Na_2_WO_4_/CS to check if another amorphous fumed silica having a specific surface area greater than 85–115 m^2^ g^-1^ gives different performance. The performance test results of two catalysts having the same active components and loading but the difference in specific surface areas (see Supplementary Table S18) showed no significant difference in the OCM performance.

#### Preparation of MnO_x_-TiO_2_-Na_2_WO_4_ nanocomposites on sol–gel SiO_2_

The MnO_x_-TiO_2_-Na_2_WO_4_ nanocomposite supported on the synthesized sol–gel SiO_2_ was also prepared using the impregnation method. The sodium tungsten dehydrate and manganese (II) nitrate tetrahydrate (Mn(NO_3_)_2_.4H_2_O, 97%, Chem-Supply) precursors were dissolved in DI water at concentrations of 0.05 M and 0.16 M, respectively, and the titanium (IV) isopropoxide (Ti[OCH(CH_3_)_2_]_4_, ≥ 97.0%, Alfa Aesar) precursor was dissolved in ethanol (C_2_H_6_O, 99.9%, QREC) at a concentration of 0.07 M. Again, the concentration of Na_2_WO_4_ was fixed at 5 wt% and the amounts of Mn and Ti were adjusted to yield the same concentrations of MnTiO_3_ (in molar ratio) as the catalysts in Sect. 4.1.3. This approach allowed for a meaningful comparison of catalytic activity among the prepared catalysts. After all the precursor solutions were added to the synthesized sol–gel SiO_2_ support, the mixture was continuously stirred at room temperature for 1 h. The mixed solution was then dried in hot-air at 100 °C for 1 h and finally calcined in the air at 800 °C for 4 h with a heating rate of 2 °C min^-1^. The so-obtained catalyst was a nanocomposite of MnO_x_-TiO_2_-Na_2_WO_4_/sol–gel SiO_2_ (denoted as Mn-Ti-NW/SG).

### Catalyst activity testing

The catalytic activity of each prepared catalyst in the OCM reaction was evaluated in a plug flow reactor. A catalyst (50 mg) was packed in a borosilicate glass tube with an inner diameter of 5 mm and sandwiched between layers of quartz wool. The feed gases were methane (CH_4_, 99.999% HP, Labgaz), oxygen (O_2_, 99.999% HP, Linde), and nitrogen (N_2_, 99.999% UHP, Labgaz). The feed gas ratio of CH_4_:O_2_:N_2_ was 3:1:4 at a total feed flow rate of 50 mL min^-1^ (GHSV = 30,558 h^-1^), which was fed into the plug flow reactor (Kejia furnace KJ-TI200). All flow rates were controlled using mass flow controllers (Aalborg GFC17) and double-checked using a bubble flow meter. The operating conditions were atmospheric pressure and a reactor temperature of 700 °C. The catalyst activity was evaluated 1 h after the system had reached the established conditions. The quantification of the gas products was carried out by gas chromatography (SHIMADZU, GC-14A) using Unibead C column connected with a thermal conductivity detector (TCD) for determining CO, CO_2_, and CH_4_ and Porapak Q column connected with a flame ionization detector (FID) for determining C_2_H_4_, C_2_H_6_, C_3_H_6_, C_3_H_8_, C_4_H_8_, and C_4_H_10_. A standard calibration curve for each product was established using five calibration points with a coefficient of determination (R^2^) > 0.995. The activity of each catalyst is presented in terms of %CH_4_ conversion, %C_2+_ selectivity, %CO_x_ selectivity, %C_2+_ yield, olefins/paraffin ratio (mol/mol), and C_2+_ formation rate (r_C2+_), which were calculated using Eqs. (1)–(6), respectively.

1 $$\% {\text{CH}}_{4} \;{\text{conversion}} = \frac{{2\left( {{\text{n}}_{{{\text{C}}_{2} {\text{H}}_{4} }} + {\text{n}}_{{{\text{C}}_{2} {\text{H}}_{6} }} } \right) + 3\left( {{\text{n}}_{{{\text{C}}_{3} {\text{H}}_{6} }} + {\text{n}}_{{{\text{C}}_{3} {\text{H}}_{8} }} } \right) + 4\left( {{\text{n}}_{{{\text{C}}_{4} {\text{H}}_{10} }} } \right) + {\text{n}}_{{{\text{CO}}}} + n_{{{\text{CO}}_{2} }} }}{{2\left( {{\text{n}}_{{{\text{C}}_{2} {\text{H}}_{4} }} + {\text{n}}_{{{\text{C}}_{2} {\text{H}}_{6} }} } \right) + 3\left( {{\text{n}}_{{{\text{C}}_{3} {\text{H}}_{6} }} + {\text{n}}_{{{\text{C}}_{3} {\text{H}}_{8} }} } \right) + 4\left( {{\text{n}}_{{{\text{C}}_{4} {\text{H}}_{10} }} } \right) + {\text{n}}_{{{\text{CO}}}} + {\text{n}}_{{{\text{CO}}_{2} }} + {\text{n}}_{{{\text{CH}}_{{4,{\text{out}}}} }} }} \times 100$$

2 $$ {\text{\% C}}_{{2 + }} \;{\text{selectivity}} = \frac{{{2}\left( {{\text{n}}_{{{\text{C}}_{{2}} {\text{H}}_{{4}} }} {\text{ + n}}_{{{\text{C}}_{{2}} {\text{H}}_{{6}} }} } \right){ + 3}\left( {{\text{n}}_{{{\text{C}}_{{3}} {\text{H}}_{{6}} }} {\text{ + n}}_{{{\text{C}}_{{3}} {\text{H}}_{{8}} }} } \right){ + 4}\left( {{\text{n}}_{{{\text{C}}_{{4}} {\text{H}}_{{{10}}} }} } \right){ }}}{{{2}\left( {{\text{n}}_{{{\text{C}}_{{2}} {\text{H}}_{{4}} }} {\text{ + n}}_{{{\text{C}}_{{2}} {\text{H}}_{{6}} }} } \right){ + 3}\left( {{\text{n}}_{{{\text{C}}_{{3}} {\text{H}}_{{6}} }} {\text{ + n}}_{{{\text{C}}_{{3}} {\text{H}}_{{8}} }} } \right){ + 4}\left( {{\text{n}}_{{{\text{C}}_{{4}} {\text{H}}_{{{10}}} }} } \right){\text{ + n}}_{{{\text{CO}}}} {\text{ + n}}_{{{\text{CO}}_{{2}} }} }} \times {100} $$

3 $$ {\text{\% CO}}_{x} {\text{ selectivity = }} \frac{{{\text{n}}_{{{\text{CO}}}} {\text{ + n}}_{{{\text{CO}}_{{2}} }} }}{{{2}\left( {{\text{n}}_{{{\text{C}}_{{2}} {\text{H}}_{{4}} }} {\text{ + n}}_{{{\text{C}}_{{2}} {\text{H}}_{{6}} }} } \right){ + 3}\left( {{\text{n}}_{{{\text{C}}_{{3}} {\text{H}}_{{6}} }} {\text{ + n}}_{{{\text{C}}_{{3}} {\text{H}}_{{8}} }} } \right){ + 4}\left( {{\text{n}}_{{{\text{C}}_{{4}} {\text{H}}_{{{10}}} }} } \right){\text{ + n}}_{{{\text{CO}}}} {\text{ + n}}_{{{\text{CO}}_{{2}} }} }} \times {100} $$

4 $$ {\text{\% C}}_{{2 + }} {\text{ yield = }} \frac{{{\text{\% CH}}_{{4}} {\text{ Conversion}} \times {\text{\% C}}_{{2 + }} {\text{ Selectivity}}}}{{{100}}} $$

5 $$\frac{{{\text{Olefin}}}}{{{\text{Paraffin}}}}{ = }\frac{{{2}\left( {{\text{n}}_{{{\text{C}}_{{2}} {\text{H}}_{{4}} }} } \right){ + 3}\left( {{\text{n}}_{{{\text{C}}_{{3}} {\text{H}}_{{6}} }} } \right){ }}}{{{2}\left( {{\text{n}}_{{{\text{C}}_{{2}} {\text{H}}_{{6}} }} } \right){ + 3}\left( {{\text{n}}_{{{\text{C}}_{{3}} {\text{H}}_{{8}} }} } \right)}}$$

6 $${\text{r}}_{{\text{C2 + }}} { = }\frac{{{2}\left( {{\text{n}}_{{{\text{C}}_{{2}} {\text{H}}_{{4}} }} {\text{ + n}}_{{{\text{C}}_{{2}} {\text{H}}_{{6}} }} } \right){ + 3}\left( {{\text{n}}_{{{\text{C}}_{{3}} {\text{H}}_{{6}} }} {\text{ + n}}_{{{\text{C}}_{{3}} {\text{H}}_{{8}} }} } \right){ + 4}\left( {{\text{n}}_{{{\text{C}}_{{4}} {\text{H}}_{{{10}}} }} } \right)}}{{{\text{(Total moles of MnTiO}}_{{3}} {\text{or (Mn + Ti) and Na}}_{{2}} {\text{WO}}_{{4}} {)} \times {\text{h}}}}$$

where n is the number of moles. The reported data is an average obtained from at least three separate catalytic tests. An example of carbon balance checks is shown in Supplementary Information Table S19.

### Catalyst characterization

The morphology and metal dispersion of the catalysts were analyzed by field emission scanning electron microscopy with energy dispersive X-Ray spectroscopy (FE-SEM/EDS, FE-SEM: JEOL JSM7600F). Before the measurements, each catalyst was sputter-coated with platinum to increase the contrast for imaging.

The structural properties of each catalyst were examined by Fourier-transform infrared spectroscopy (FTIR; PerkinElmer Spectrum 400 FT-IR/FT-FIR). For each spectrum, 32 scans were collected over a spectral range of 400–4,000 cm^-1^at a resolution of 4 cm^-1^.

The crystal structure of each catalyst was analyzed by powder X-ray diffractometry (XRD; Bruker D8 Advance), using Cu-Kα radiation at 45 kV and 40 mA with a step size of 0.02° and a step time of 0.5 s.

The surface area, pore size, and pore volume of each catalyst were measured by an N_2_-adsorption analyzer (3Flex Physisorption Micromeritics), following the Brunauer–Emmett–Teller (BET) method being conducted at −196 °C.

X-ray photoelectron spectroscopy (XPS; Kratos Axis Ultra DLD) was used to characterize the elements in each catalyst, namely sodium (Na 1 s), tungsten (W 4f.), manganese (Mn 2p), titanium (Ti 2p), silicon (Si 2p), and oxygen (O 1 s). The binding energy of C 1 s (285.0 eV) was used as a standard for all other binding energies.

The particle size distribution of the samples was analyzed using high resolution–transmission electron microscopy with energy-dispersive X-ray spectroscopy (HR-TEM: JEM-2100). Each sample was suspended in ethanol solvent, dropped on carbon film coated on Cu TEM grids, and dried in a chamber filled with N_2_ at room temperature before the analysis.

## Supplementary Information

Supplementary Information.
