# Supplementary material for: Oxidative coupling of methane—comparisons of MnTiO3–Na2WO4 and MnOx–TiO2–Na2WO4 catalysts on different silica supports
Source: Sci Rep. 2022 Feb 16;12:2595. doi: 10.1038/s41598-022-06598-6 (PMC8850452; doi:10.1038/s41598-022-06598-6)
Supplement: Supplementary file 1 — Supplementary Information. [file 41598_2022_6598_MOESM1_ESM.docx]

**Supplementary Information**

**Oxidative coupling of methane—Comparisons of MnTiO_3_-Na_2_WO_4_ and MnO_x_-TiO_2_-Na_2_WO_4_ catalysts on different silica supports**

*Worapinit Tiyatha^1,2^, Thanaphat Chukeaw^1,2^, Sarannuch Sringam^1,2^, Thongthai Witoon^1,2,3^, Metta Chareonpanich^1,2,3^, Günther Rupprechter^4^, Anusorn Seubsai^1,2,3^**

*^1^ Department of Chemical Engineering, Faculty of Engineering, Kasetsart University, Bangkok 10900, Thailand*

*^2^ Center of Excellence on Petrochemical and Materials Technology, Kasetsart University, Bangkok 10900, Thailand*

*^3^ Research Network of NANOTEC–KU on NanoCatalysts and NanoMaterials for Sustainable Energy and Environment, Kasetsart University, Bangkok 10900, Thailand*

*^4^ Institute of Materials Chemistry, TU Wien, 1060 Vienna, Austria*

***Corresponding author**: fengasn@ku.ac.th

**Supplementary Figure S1** Activity in OCM reaction: a) MnTiO_3_-NW/CS, b) MnTiO_3_-NW/SG, and c) Mn-Ti-NW/SG catalysts. Testing conditions: 50 mg catalyst, gas feed of CH_4_:O_2_:N_2_ = 3:1:4, reactor temperature = 700 °C, atmospheric pressure, total gas flow rate = 50 mL min^-1^ (GHSV = 30,588 h^-1^).

**Supplementary Table S1** Characteristic XRD values (2θ) crystalline phases found in different catalysts.

| **Catalyst** | **Crystalline phase** | **2θ (degree)** | **ICDD No.** |
| --- | --- | --- | --- |
| NW/CS | Sodium tungsten oxide (Na_2_WO_4_) | 16.806, 27.612, 32.499, 48.828, 52.004, 57.015 | 01-074-2369 |
|  | α-cristobalite (SiO_2_) | 21.947, 28.401, 31.375, 36.056 | 01-071-0785 |
|  | α-tridymite (SiO_2_) | 23.440, 24.443, 29.625, 30.064, 35.853 | 01-086-0681 |
| 5MnTiO_3_-NW/CS | Sodium tungsten oxide (Na_2_WO_4_) | 16.806, 27.612, 32.499, 48.828, 52.004, 57.015 | 01-074-2369 |
|  | α-cristobalite (SiO_2_) | 21.947, 28.401, 31.375, 36.056 | 01-071-0785 |
|  | Manganese oxide (Mn_2_O_3_) | 21.101, 32.930, 38.171, 55.110 | 01-071-0635 |
|  | Manganese oxide (Mn_3_O_4_) | 31.138, 32.533, 36.191, 38.101, 44.600 | 00-001-1127 |
|  | Rutile (TiO_2_) | 27.436, 36.080, 41.240, 54.321, 56.759 | 01-075-1753 |
|  | Manganese titanium oxide (MnTiO_3_) | 41.385, 48.104, 52.262, 55.368 | 00-002-0846 |
|  | α-tridymite (SiO_2_) | 23.440, 24.443, 29.625, 30.064, 35.853 | 01-086-0681 |
| 20MnTiO_3_-NW/CS | Sodium tungsten oxide (Na_2_WO_4_) | 16.806, 27.612, 32.499, 48.828, 52.004, 57.015 | 01-074-2369 |
|  | α-cristobalite (SiO_2_) | 21.947, 28.401, 31.375, 36.056 | 01-071-0785 |
|  | Manganese oxide (Mn_2_O_3_) | 21.101, 32.930, 38.171, 55.110 | 01-071-0635 |
|  | Manganese oxide (Mn_3_O_4_) | 31.138, 32.533, 36.191, 38.101, 44.600 | 00-001-1127 |
|  | Rutile (TiO_2_) | 27.436, 36.080, 41.240, 54.321, 56.759 | 01-075-1753 |
|  | Manganese titanium oxide (MnTiO_3_) | 41.385, 48.104, 52.262, 55.368 | 00-002-0846 |
|  | α-tridymite (SiO_2_) | 23.440, 24.443, 29.625, 30.064, 35.853 | 01-086-0681 |
| NW/SG | Sodium tungsten oxide (Na_2_WO_4_) | 16.806, 27.612, 32.499, 48.828, 52.004, 57.015 | 01-074-2369 |
|  | α-cristobalite (SiO_2_) | 21.947, 28.401, 31.375, 36.056 | 01-071-0785 |
|  | α-tridymite (SiO_2_) | 23.440, 24.443, 29.625, 30.064, 35.853 | 01-086-0681 |
| 10MnTiO_3_-NW/SG | Sodium tungsten oxide (Na_2_WO_4_) | 16.806, 27.612, 32.499, 48.828, 52.004, 57.015 | 01-074-2369 |
|  | α-cristobalite (SiO_2_) | 21.947, 28.401, 31.375, 36.056 | 01-071-0785 |
|  | Manganese oxide (Mn_2_O_3_) | 21.101, 32.930, 38.171, 55.110 | 01-071-0635 |
|  | Manganese oxide (Mn_3_O_4_) | 31.138, 32.533, 36.191, 38.101, 44.600 | 00-001-1127 |
|  | Rutile (TiO_2_) | 27.436, 36.080, 41.240, 54.321, 56.759 | 01-075-1753 |
|  | Manganese titanium oxide (MnTiO_3_) | 41.385, 48.104, 52.262, 55.368 | 00-002-0846 |
|  | Quartz (SiO_2_) | 20.861, 26.642, 50.145 | 01-075-0443 |

**Supplementary Table S1** Characteristic XRD values (2θ) crystalline phases found in different catalysts. (Continued)

| **Catalyst** | **Crystalline phase** | **2θ (degree)** | **ICDD No.** |
| --- | --- | --- | --- |
| 10MnTiO3-NW/SG | α-tridymite (SiO_2_) | 23.440, 24.443, 29.625, 30.064, 35.853 | 01-086-0681 |
| 20MnTiO_3_-NW/SG | Sodium tungsten oxide (Na_2_WO_4_) | 16.806, 27.612, 32.499, 48.828, 52.004, 57.015 | 01-074-2369 |
|  | α-cristobalite (SiO_2_) | 21.947, 28.401, 31.375, 36.056 | 01-071-0785 |
|  | Manganese oxide (Mn_2_O_3_) | 21.101, 32.930, 38.171, 55.110 | 01-071-0635 |
|  | Manganese oxide (Mn_3_O_4_) | 31.138, 32.533, 36.191, 38.101, 44.600 | 00-001-1127 |
|  | Rutile (TiO_2_) | 27.436, 36.080, 41.240, 54.321, 56.759 | 01-075-1753 |
|  | Manganese titanium oxide (MnTiO_3_) | 41.385, 48.104, 52.262, 55.368 | 00-002-0846 |
|  | Quartz (SiO_2_) | 20.861, 26.642, 50.145 | 01-075-0443 |
|  | α-tridymite (SiO_2_) | 23.440, 24.443, 29.625, 30.064, 35.853 | 01-086-0681 |
| 5Mn-Ti-NW/SG | Sodium tungsten oxide (Na_2_WO_4_) | 16.806, 27.612, 32.499, 48.828, 52.004, 57.015 | 01-074-2369 |
|  | α-cristobalite (SiO_2_) | 21.947, 28.401, 31.375, 36.056 | 01-071-0785 |
|  | Manganese oxide (Mn_2_O_3_) | 21.101, 32.930, 38.171, 55.110 | 01-071-0635 |
|  | Manganese oxide (Mn_3_O_4_) | 31.138, 32.533, 36.191, 38.101, 44.600 | 00-001-1127 |
|  | Rutile (TiO_2_) | 27.436, 36.080, 41.240, 54.321, 56.759 | 01-075-1753 |
|  | α-tridymite (SiO_2_) | 23.440, 24.443, 29.625, 30.064, 35.853 | 01-086-0681 |
| 20Mn-Ti-NW/SG | Sodium tungsten oxide (Na_2_WO_4_) | 16.806, 27.612, 32.499, 48.828, 52.004, 57.015 | 01-074-2369 |
|  | α-cristobalite (SiO_2_) | 21.947, 28.401, 31.375, 36.056 | 01-071-0785 |
|  | Manganese oxide (Mn_2_O_3_) | 21.101, 32.930, 38.171, 55.110 | 01-071-0635 |
|  | Manganese oxide (Mn_3_O_4_) | 31.138, 32.533, 36.191, 38.101, 44.600 | 00-001-1127 |
|  | Rutile (TiO_2_) | 27.436, 36.080, 41.240, 54.321, 56.759 | 01-075-1753 |
|  | α-tridymite (SiO_2_) | 23.440, 24.443, 29.625, 30.064, 35.853 | 01-086-0681 |
| Used MnTiO_3_-NW/CS | Sodium tungsten oxide (Na_2_WO_4_) | 16.806, 27.612, 32.499, 48.828, 52.004, 57.015 | 01-074-2369 |
|  | α-cristobalite (SiO_2_) | 21.947, 28.401, 31.375, 36.056 | 01-071-0785 |
|  | Manganese oxide (Mn_2_O_3_) | 21.101, 32.930, 38.171, 55.110 | 01-071-0635 |
|  | Manganese oxide (Mn_3_O_4_) | 31.138, 32.533, 36.191, 38.101, 44.600 | 00-001-1127 |
|  | Rutile (TiO_2_) | 27.436, 36.080, 41.240, 54.321, 56.759 | 01-075-1753 |
|  | Manganese titanium oxide (MnTiO_3_) | 41.385, 48.104, 52.262, 55.368 | 00-002-0846 |
|  | Quartz (SiO_2_) | 20.861, 26.642, 50.145 | 01-075-0443 |
|  | α-tridymite (SiO_2_) | 23.440, 24.443, 29.625, 30.064, 35.853 | 01-086-0681 |

**Supplementary Table S1** Characteristic XRD values (2θ) crystalline phases found in different catalysts. (Continued)

| **Catalyst** | **Crystalline phase** | **2θ (degree)** | **ICDD No.** |
| --- | --- | --- | --- |
| Used MnTiO_3_-NW/SG | Sodium tungsten oxide (Na_2_WO_4_) | 16.806, 27.612, 32.499, 48.828, 52.004, 57.015 | 01-074-2369 |
|  | α-cristobalite (SiO_2_) | 21.947, 28.401, 31.375, 36.056 | 01-071-0785 |
|  | Manganese oxide (Mn_2_O_3_) | 21.101, 32.930, 38.171, 55.110 | 01-071-0635 |
|  | Manganese oxide (Mn_3_O_4_) | 31.138, 32.533, 36.191, 38.101, 44.600 | 00-001-1127 |
|  | Rutile (TiO_2_) | 27.436, 36.080, 41.240, 54.321, 56.759 | 01-075-1753 |
|  | Manganese titanium oxide (MnTiO_3_) | 41.385, 48.104, 52.262, 55.368 | 00-002-0846 |
|  | Quartz (SiO_2_) | 20.861, 26.642, 50.145 | 01-075-0443 |
|  | α-tridymite (SiO_2_) | 23.440, 24.443, 29.625, 30.064, 35.853 | 01-086-0681 |
| Used Mn-Ti-NW/SG | Sodium tungsten oxide (Na_2_WO_4_) | 16.806, 27.612, 32.499, 48.828, 52.004, 57.015 | 01-074-2369 |
|  | α-cristobalite (SiO_2_) | 21.947, 28.401, 31.375, 36.056 | 01-071-0785 |
|  | Manganese oxide (Mn_2_O_3_) | 21.101, 32.930, 38.171, 55.110 | 01-071-0635 |
|  | Manganese oxide (Mn_3_O_4_) | 31.138, 32.533, 36.191, 38.101, 44.600 | 00-001-1127 |
|  | Rutile (TiO_2_) | 27.436, 36.080, 41.240, 54.321, 56.759 | 01-075-1753 |
|  | α-tridymite (SiO_2_) | 23.440, 24.443, 29.625, 30.064, 35.853 | 01-086-0681 |


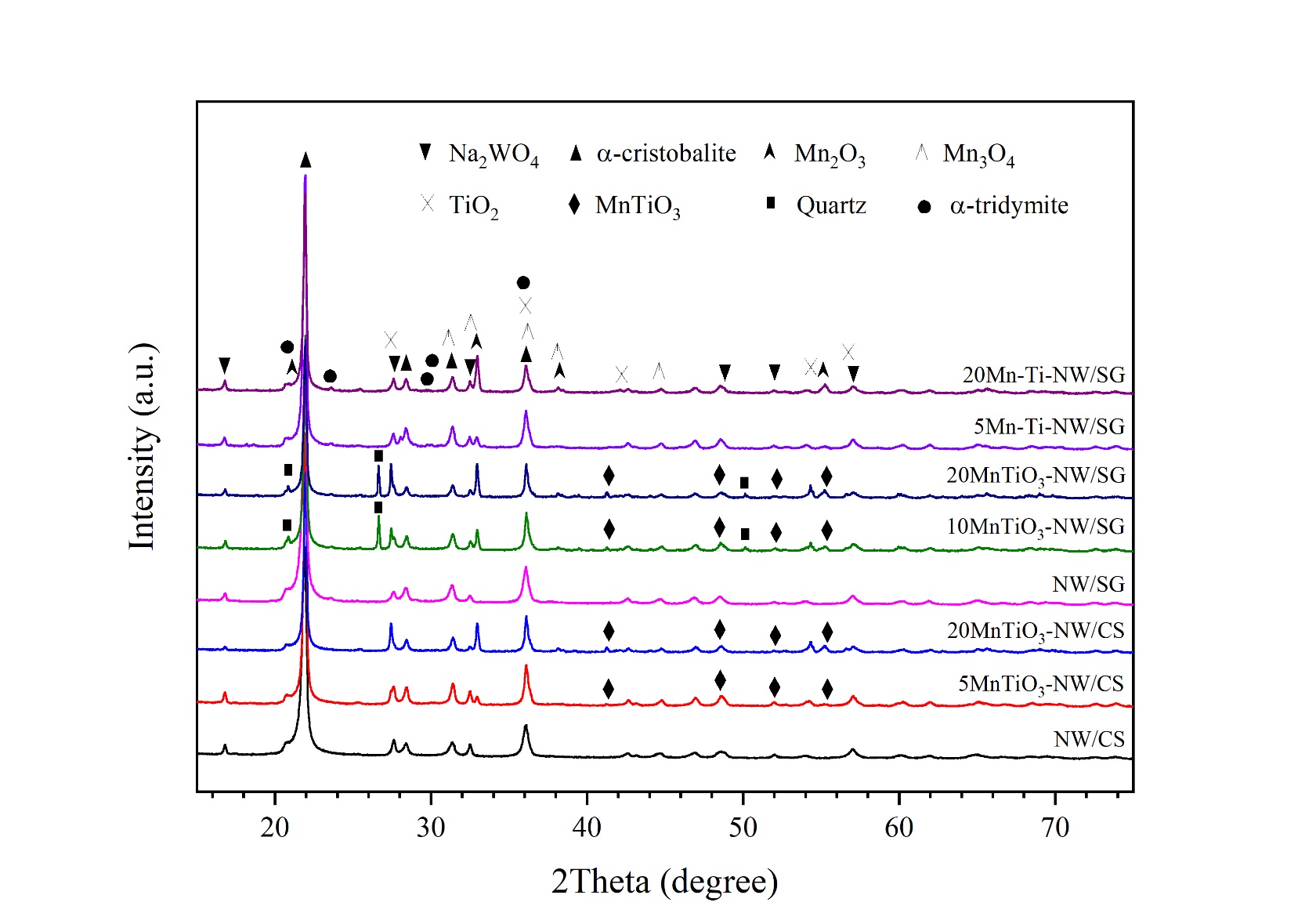


**Supplementary Figure S2** XRD patterns of catalysts.


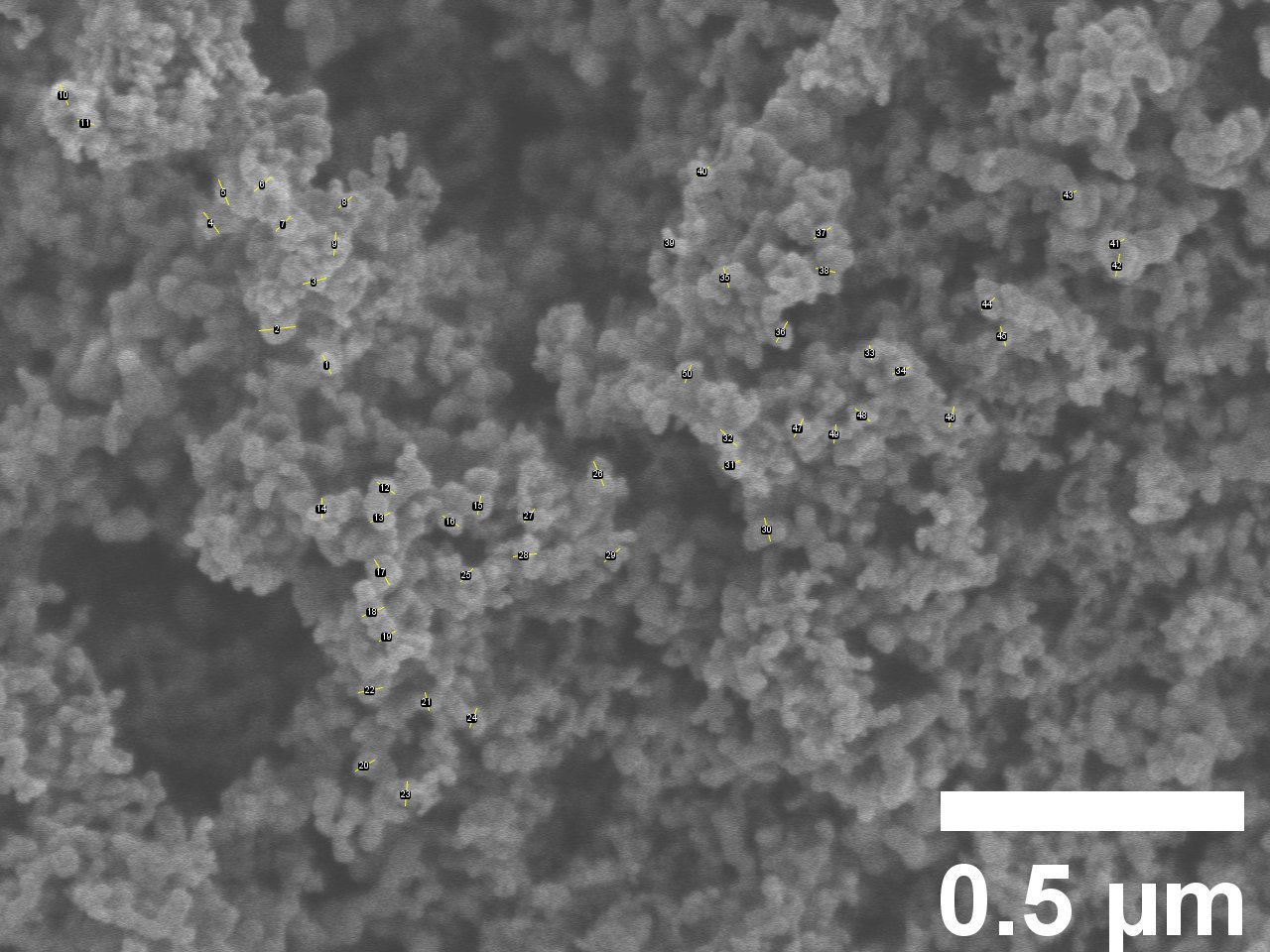


**Supplementary Figure S3** The average particle size of CS.

**Supplementary Table S2** The average particle size of CS.

| No. | Length (μm) | No. | Length (μm) | No. | Length (μm) | No. | Length (μm) | No. | Length (μm) |
| --- | --- | --- | --- | --- | --- | --- | --- | --- | --- |
| 1 | 0.032 | 11 | 0.027 | 21 | 0.030 | 31 | 0.031 | 41 | 0.028 |
| 2 | 0.060 | 12 | 0.033 | 22 | 0.040 | 32 | 0.037 | 42 | 0.037 |
| 3 | 0.039 | 13 | 0.035 | 23 | 0.040 | 33 | 0.023 | 43 | 0.027 |
| 4 | 0.041 | 14 | 0.033 | 24 | 0.033 | 34 | 0.028 | 44 | 0.029 |
| 5 | 0.044 | 15 | 0.030 | 25 | 0.029 | 35 | 0.032 | 45 | 0.032 |
| 6 | 0.036 | 16 | 0.031 | 26 | 0.043 | 36 | 0.038 | 46 | 0.034 |
| 7 | 0.033 | 17 | 0.048 | 27 | 0.027 | 37 | 0.033 | 47 | 0.033 |
| 8 | 0.028 | 18 | 0.038 | 28 | 0.038 | 38 | 0.032 | 48 | 0.030 |
| 9 | 0.038 | 19 | 0.031 | 29 | 0.033 | 39 | 0.015 | 49 | 0.030 |
| 10 | 0.035 | 20 | 0.036 | 30 | 0.039 | 40 | 0.025 | 50 | 0.030 |
|  |  |  |  |  |  |  |  | **Avg.** | **0.034** |


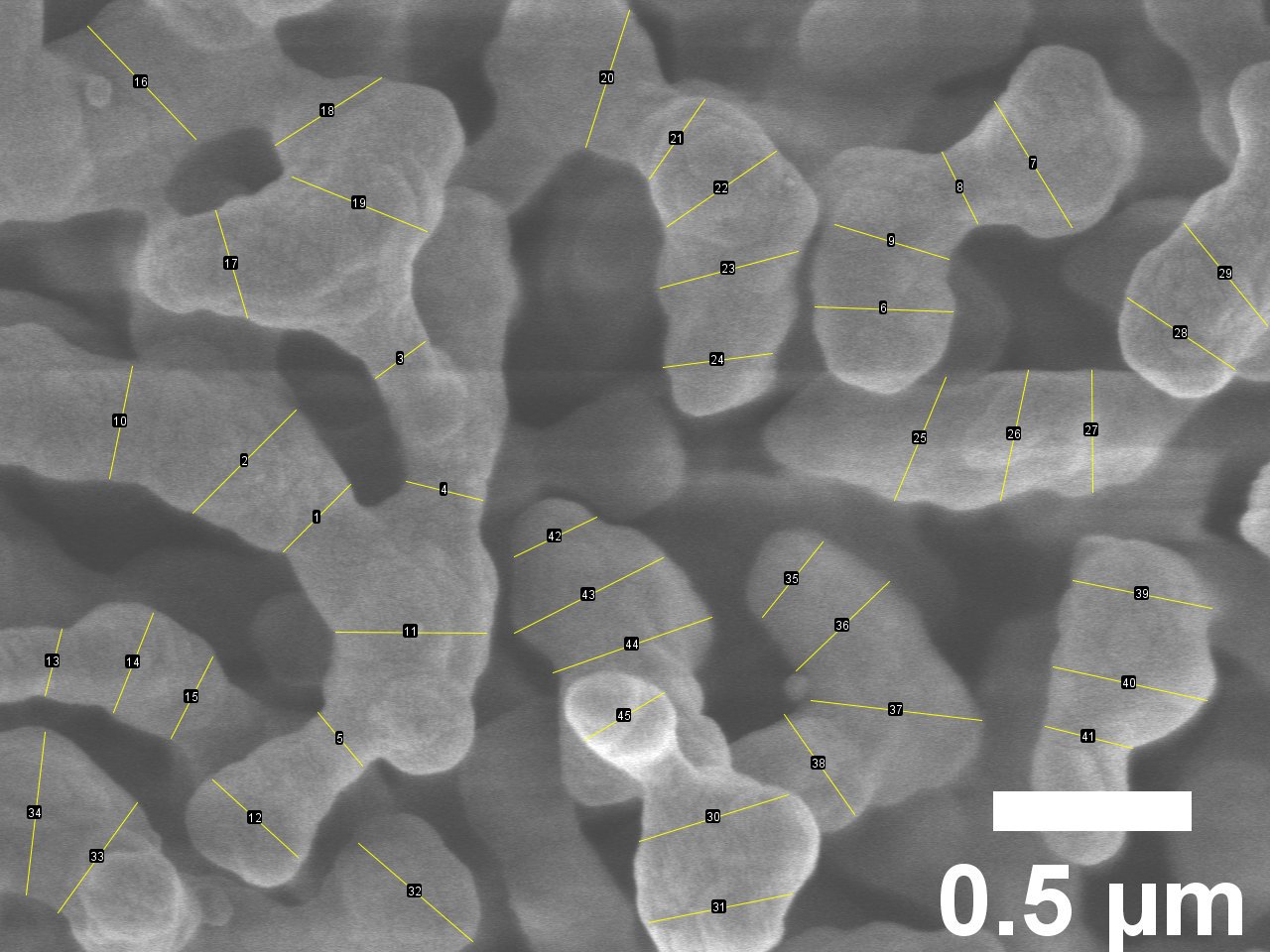


**Supplementary Figure S4** The average particle size of NW/CS.

**Supplementary Table S3** The average particle size of NW/CS.

| No. | Length (μm) | No. | Length (μm) | No. | Length (μm) | No. | Length (μm) | No. | Length (μm) |
| --- | --- | --- | --- | --- | --- | --- | --- | --- | --- |
| 1 | 0.240 | 11 | 0.380 | 21 | 0.244 | 31 | 0.370 | 41 | 0.227 |
| 2 | 0.368 | 12 | 0.290 | 22 | 0.334 | 32 | 0.379 | 42 | 0.230 |
| 3 | 0.156 | 13 | 0.173 | 23 | 0.360 | 33 | 0.341 | 43 | 0.420 |
| 4 | 0.198 | 14 | 0.269 | 24 | 0.279 | 34 | 0.413 | 44 | 0.424 |
| 5 | 0.176 | 15 | 0.230 | 25 | 0.337 | 35 | 0.244 | 45 | 0.234 |
| 6 | 0.348 | 16 | 0.394 | 26 | 0.335 | 36 | 0.325 | **Avg.** | **0.312** |
| 7 | 0.373 | 17 | 0.283 | 27 | 0.308 | 37 | 0.433 |  |  |
| 8 | 0.201 | 18 | 0.317 | 28 | 0.325 | 38 | 0.309 |  |  |
| 9 | 0.301 | 19 | 0.367 | 29 | 0.332 | 39 | 0.357 |  |  |
| 10 | 0.289 | 20 | 0.362 | 30 | 0.393 | 40 | 0.397 |  |  |


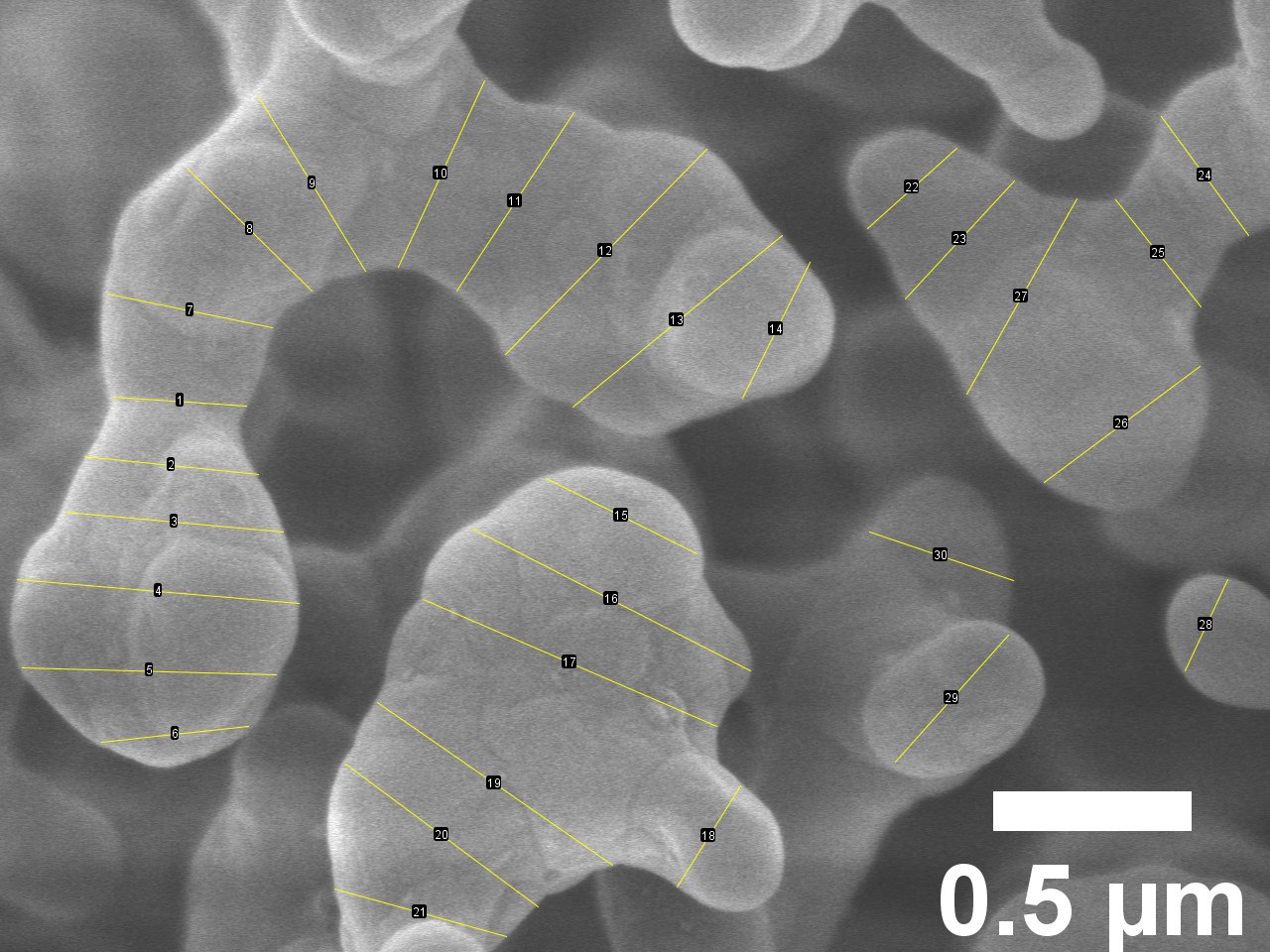


**Supplementary Figure S5** The average particle size of 5MnTiO_3_-NW/CS.

**Supplementary Table S4** The average particle size of 5MnTiO_3_-NW/CS.

| No. | Length (μm) | No. | Length (μm) | No. | Length (μm) |
| --- | --- | --- | --- | --- | --- |
| 1 | 0.336 | 11 | 0.538 | 21 | 0.449 |
| 2 | 0.440 | 12 | 0.725 | 22 | 0.303 |
| 3 | 0.550 | 13 | 0.682 | 23 | 0.405 |
| 4 | 0.713 | 14 | 0.383 | 24 | 0.372 |
| 5 | 0.640 | 15 | 0.425 | 25 | 0.345 |
| 6 | 0.372 | 16 | 0.787 | 26 | 0.490 |
| 7 | 0.424 | 17 | 0.806 | 27 | 0.566 |
| 8 | 0.442 | 18 | 0.301 | 28 | 0.254 |
| 9 | 0.516 | 19 | 0.721 | 29 | 0.429 |
| 10 | 0.518 | 20 | 0.604 | 30 | 0.383 |
|  |  |  |  | **Avg.** | **0.497** |


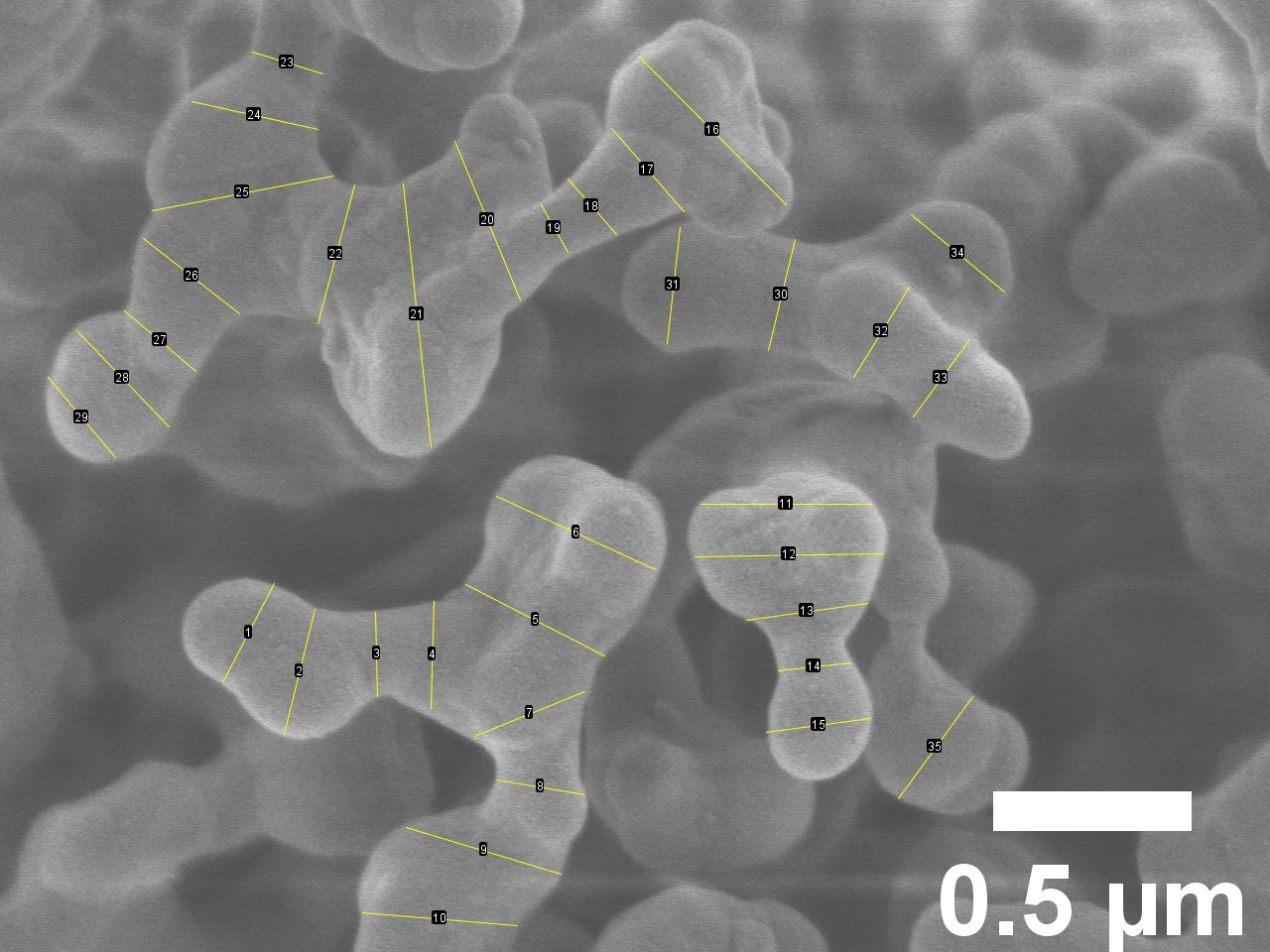


**Supplementary Figure S6** The average particle size of 20MnTiO_3_-NW/CS.

**Supplementary Table S5** The average particle size of 20MnTiO_3_-NW/CS.

| No. | Length (μm) | No. | Length (μm) | No. | Length (μm) | No. | Length (μm) |
| --- | --- | --- | --- | --- | --- | --- | --- |
| 1 | 0.281 | 11 | 0.430 | 21 | 0.666 | 31 | 0.294 |
| 2 | 0.324 | 12 | 0.475 | 22 | 0.362 | 32 | 0.267 |
| 3 | 0.212 | 13 | 0.308 | 23 | 0.186 | 33 | 0.242 |
| 4 | 0.270 | 14 | 0.184 | 24 | 0.325 | 34 | 0.305 |
| 5 | 0.394 | 15 | 0.266 | 25 | 0.466 | 35 | 0.318 |
| 6 | 0.441 | 16 | 0.526 | 26 | 0.310 | **Avg.** | **0.324** |
| 7 | 0.299 | 17 | 0.278 | 27 | 0.236 |  |  |
| 8 | 0.226 | 18 | 0.186 | 28 | 0.336 |  |  |
| 9 | 0.408 | 19 | 0.138 | 29 | 0.266 |  |  |
| 10 | 0.391 | 20 | 0.433 | 30 | 0.288 |  |  |


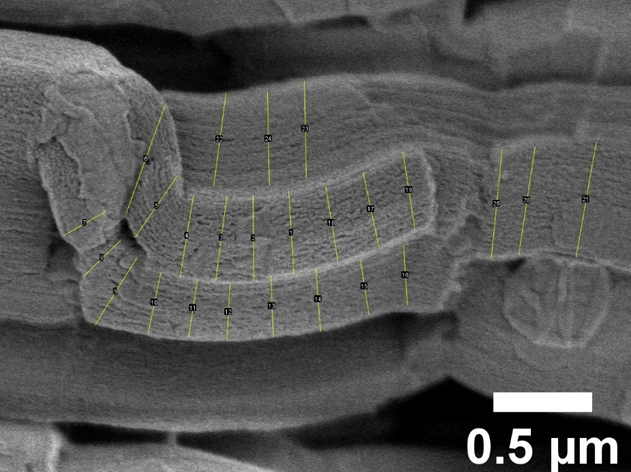


**Supplementary Figure S7** The average particle size of SG.

**Supplementary Table S6** The average particle size of SG.

| No. | Length (μm) | No. | Length (μm) | No. | Length (μm) | No. | Length (μm) | No. | Length (μm) |
| --- | --- | --- | --- | --- | --- | --- | --- | --- | --- |
| 1 | 0.426 | 6 | 0.579 | 11 | 0.296 | 16 | 0.311 | 21 | 0.591 |
| 2 | 0.430 | 7 | 0.245 | 12 | 0.288 | 17 | 0.383 | 22 | 0.477 |
| 3 | 0.434 | 8 | 0.262 | 13 | 0.310 | 18 | 0.385 | 23 | 0.493 |
| 4 | 0.416 | 9 | 0.401 | 14 | 0.323 | 19 | 0.395 | 24 | 0.470 |
| 5 | 0.379 | 10 | 0.317 | 15 | 0.284 | 20 | 0.551 | 25 | 0.553 |
|  |  |  |  |  |  |  |  | **Avg.** | **0.400** |


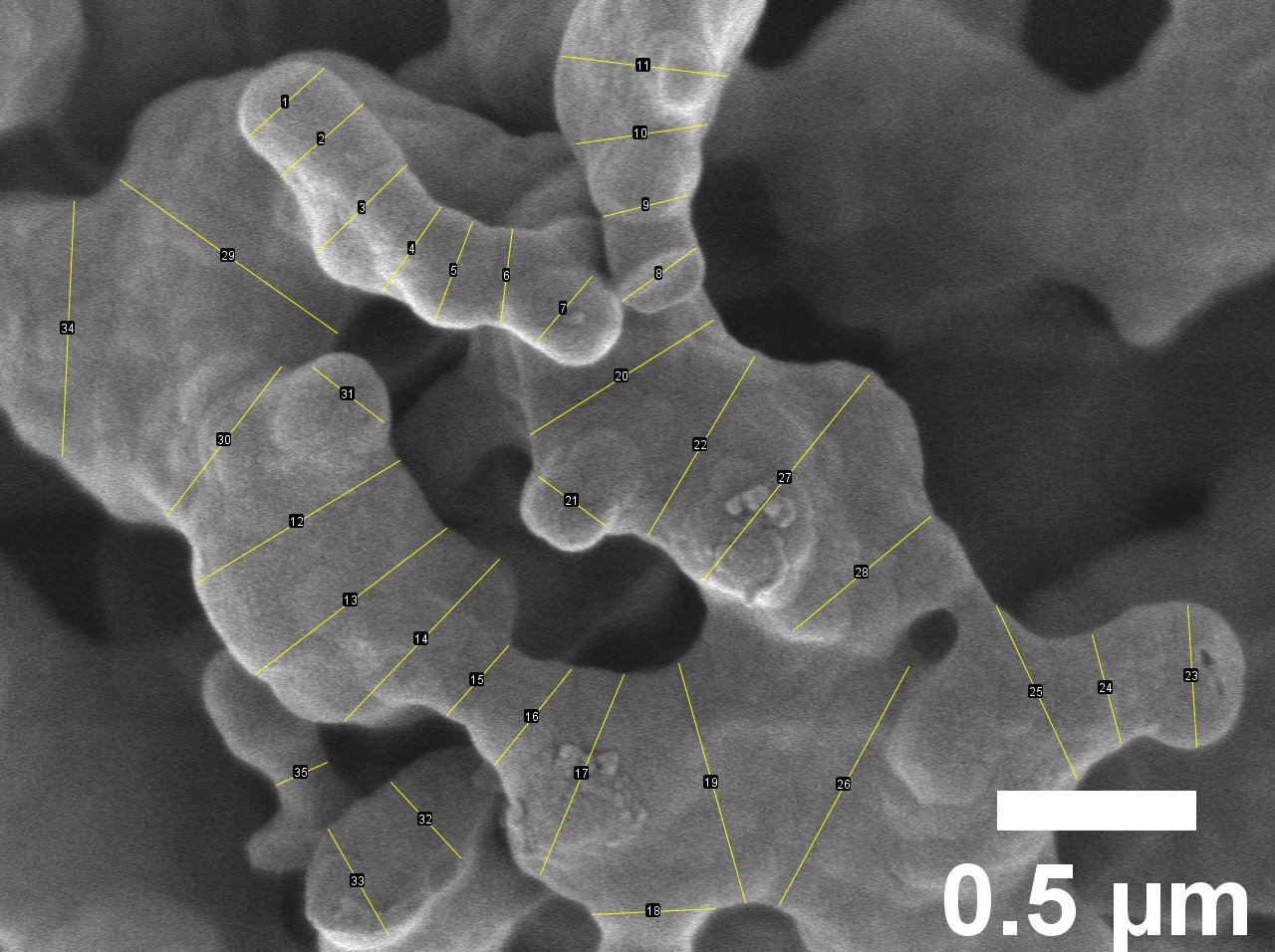


**Supplementary Figure S8** The average particle size of NW/SG.

**Supplementary Table S7** The average particle size of NW/SG.

| No. | Length (μm) | No. | Length (μm) | No. | Length (μm) | No. | Length (μm) |
| --- | --- | --- | --- | --- | --- | --- | --- |
| 1 | 0.260 | 11 | 0.416 | 21 | 0.211 | 31 | 0.228 |
| 2 | 0.276 | 12 | 0.599 | 22 | 0.521 | 32 | 0.258 |
| 3 | 0.315 | 13 | 0.605 | 23 | 0.356 | 33 | 0.305 |
| 4 | 0.255 | 14 | 0.564 | 24 | 0.282 | 34 | 0.641 |
| 5 | 0.267 | 15 | 0.234 | 25 | 0.480 | 35 | 0.140 |
| 6 | 0.239 | 16 | 0.308 | 26 | 0.680 | **Avg.** | **0.392** |
| 7 | 0.220 | 17 | 0.545 | 27 | 0.668 |  |  |
| 8 | 0.228 | 18 | 0.310 | 28 | 0.449 |  |  |
| 9 | 0.216 | 19 | 0.623 | 29 | 0.667 |  |  |
| 10 | 0.331 | 20 | 0.542 | 30 | 0.469 |  |  |


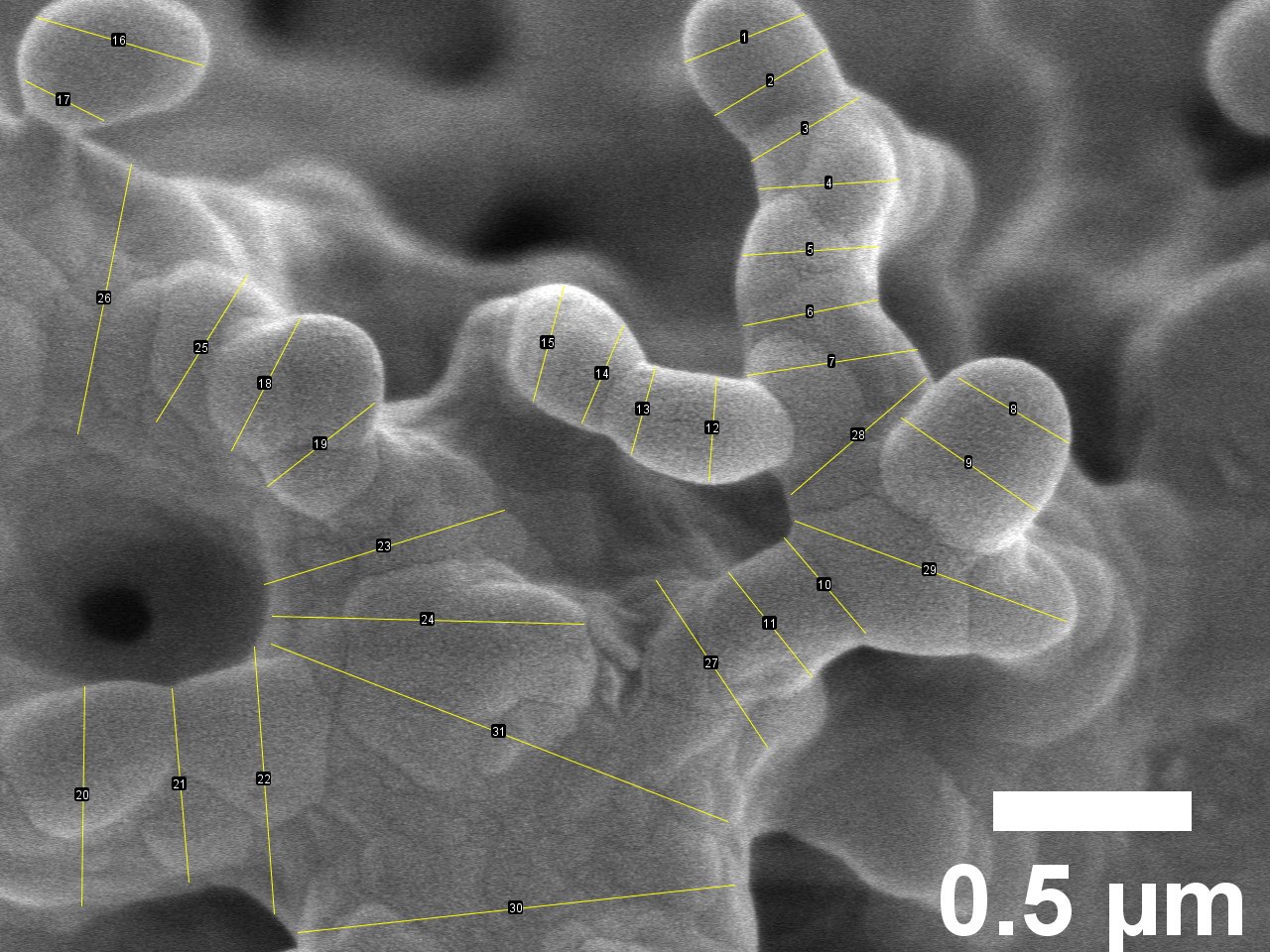


**Supplementary Figure S9** The average particle size of 10MnTiO_3_-NW/SG.

**Supplementary Table S8** The average particle size of 10MnTiO_3_-NW/SG.

| No. | Length (μm) | No. | Length (μm) | No. | Length (μm) | No. | Length (μm) |
| --- | --- | --- | --- | --- | --- | --- | --- |
| 1 | 0.323 | 11 | 0.336 | 21 | 0.489 | 31 | 1.234 |
| 2 | 0.328 | 12 | 0.261 | 22 | 0.674 | **Avg.** | **0.469** |
| 3 | 0.314 | 13 | 0.226 | 23 | 0.633 |  |  |
| 4 | 0.351 | 14 | 0.267 | 24 | 0.785 |  |  |
| 5 | 0.341 | 15 | 0.300 | 25 | 0.436 |  |  |
| 6 | 0.344 | 16 | 0.440 | 26 | 0.693 |  |  |
| 7 | 0.432 | 17 | 0.221 | 27 | 0.505 |  |  |
| 8 | 0.319 | 18 | 0.375 | 28 | 0.449 |  |  |
| 9 | 0.412 | 19 | 0.342 | 29 | 0.728 |  |  |
| 10 | 0.316 | 20 | 0.553 | 30 | 1.107 |  |  |


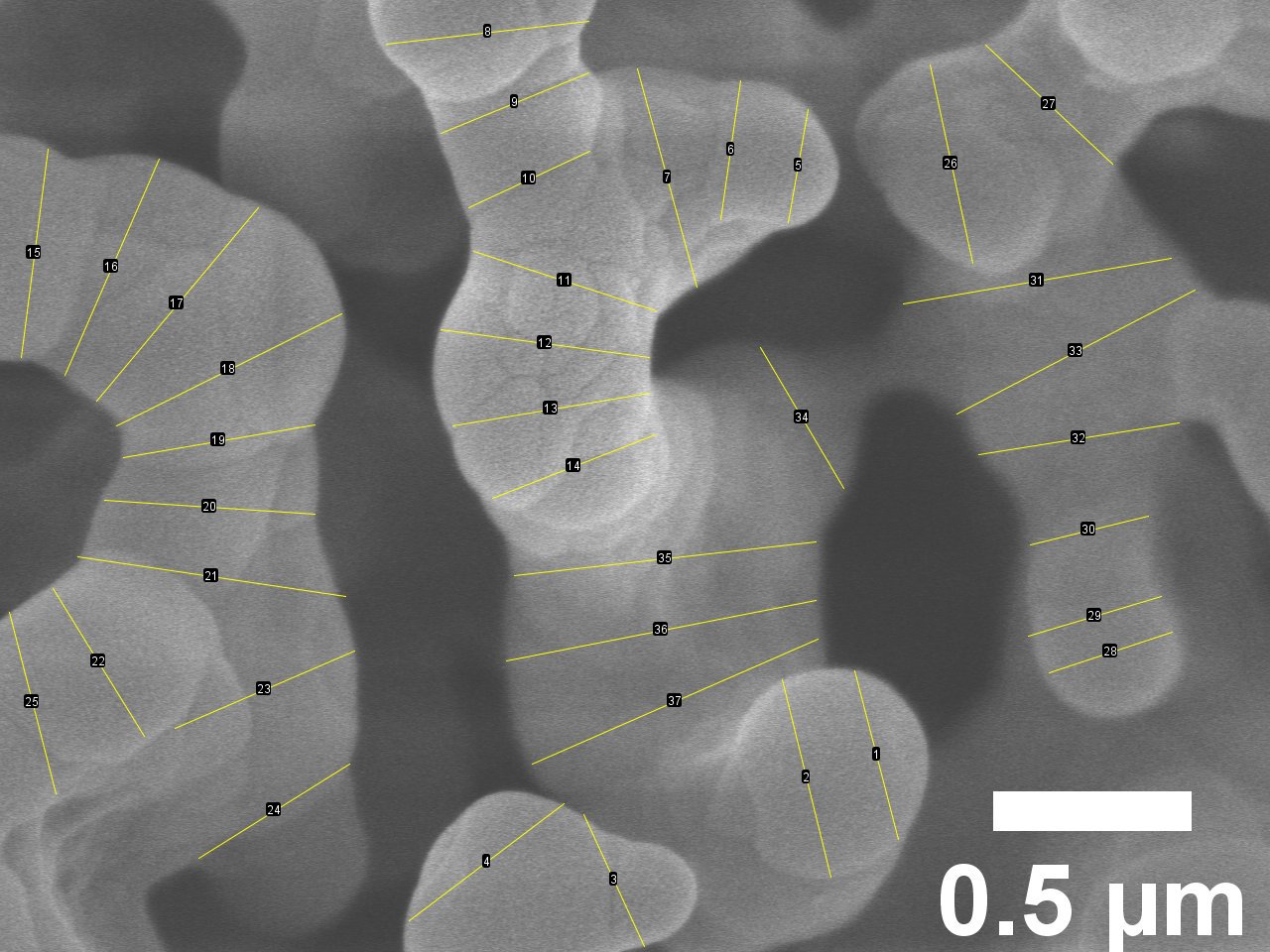


**Supplementary Figure S10** The average particle size of 20MnTiO_3_-NW/SG.

**Supplementary Table S9** The average particle size of 20MnTiO_3_-NW/SG.

| No. | Length (μm) | No. | Length (μm) | No. | Length (μm) | No. | Length (μm) |
| --- | --- | --- | --- | --- | --- | --- | --- |
| 1 | 0.439 | 11 | 0.484 | 21 | 0.682 | 31 | 0.685 |
| 2 | 0.512 | 12 | 0.530 | 22 | 0.438 | 32 | 0.511 |
| 3 | 0.366 | 13 | 0.502 | 23 | 0.493 | 33 | 0.677 |
| 4 | 0.489 | 14 | 0.440 | 24 | 0.448 | 34 | 0.412 |
| 5 | 0.289 | 15 | 0.529 | 25 | 0.472 | 35 | 0.765 |
| 6 | 0.354 | 16 | 0.595 | 26 | 0.511 | 36 | 0.795 |
| 7 | 0.570 | 17 | 0.635 | 27 | 0.439 | 37 | 0.786 |
| 8 | 0.513 | 18 | 0.634 | 28 | 0.327 | **Avg.** | **0.506** |
| 9 | 0.403 | 19 | 0.490 | 29 | 0.350 |  |  |
| 10 | 0.337 | 20 | 0.531 | 30 | 0.306 |  |  |

**
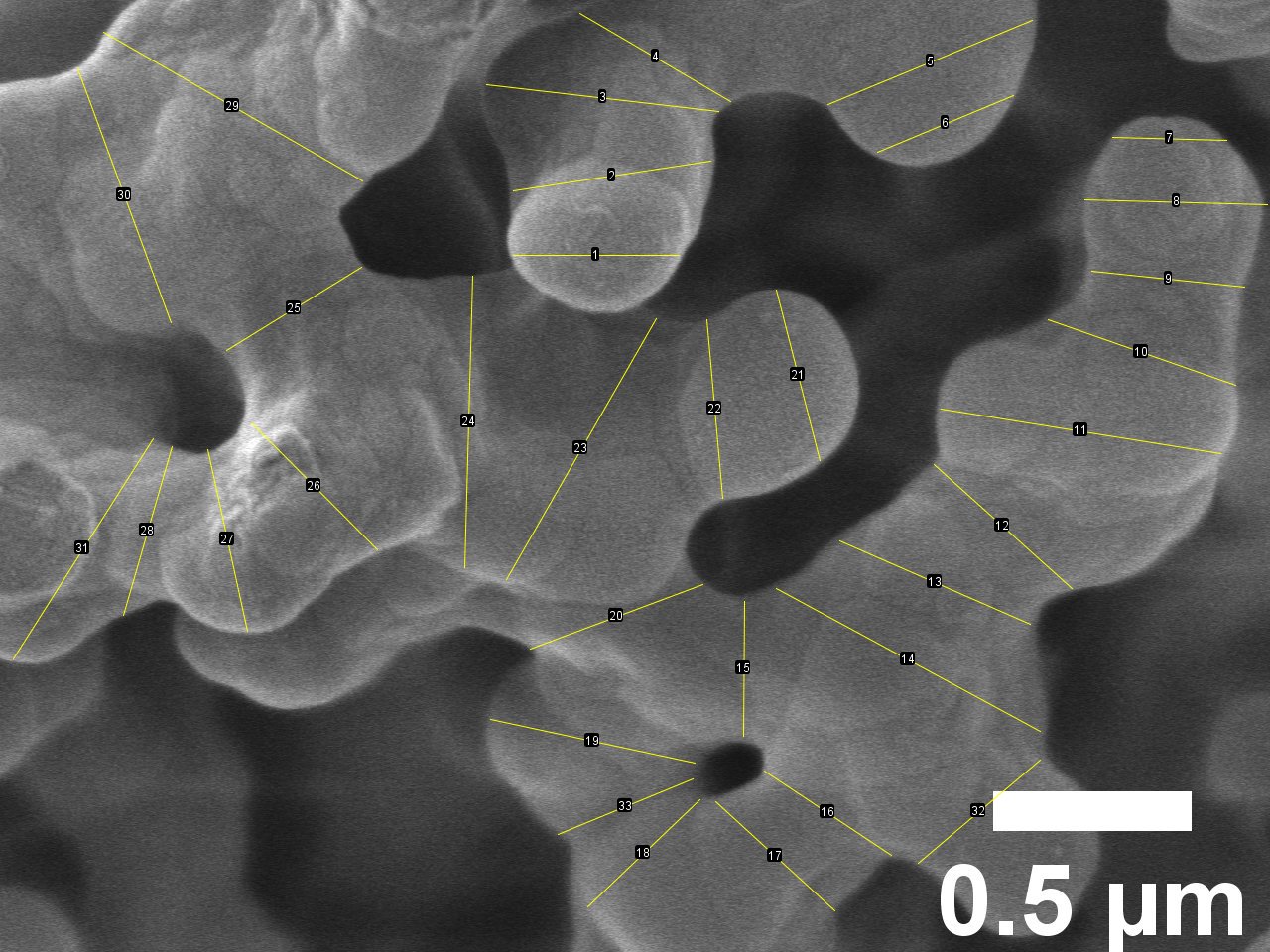
**

**Supplementary Figure S11** The average particle size of 5Mn-Ti-NW/SG.

**Supplementary Table S10** The average particle size of 5Mn-Ti-NW/SG.

| No. | Length (μm) | No. | Length (μm) | No. | Length (μm) | No. | Length (μm) |
| --- | --- | --- | --- | --- | --- | --- | --- |
| 1 | 0.418 | 11 | 0.714 | 21 | 0.444 | 31 | 0.657 |
| 2 | 0.503 | 12 | 0.467 | 22 | 0.452 | 32 | 0.403 |
| 3 | 0.589 | 13 | 0.524 | 23 | 0.758 | 33 | 0.368 |
| 4 | 0.440 | 14 | 0.756 | 24 | 0.735 | **Avg.** | **0.500** |
| 5 | 0.557 | 15 | 0.340 | 25 | 0.400 |  |  |
| 6 | 0.371 | 16 | 0.384 | 26 | 0.451 |  |  |
| 7 | 0.288 | 17 | 0.407 | 27 | 0.468 |  |  |
| 8 | 0.461 | 18 | 0.391 | 28 | 0.442 |  |  |
| 9 | 0.387 | 19 | 0.527 | 29 | 0.753 |  |  |
| 10 | 0.500 | 20 | 0.464 | 30 | 0.682 |  |  |


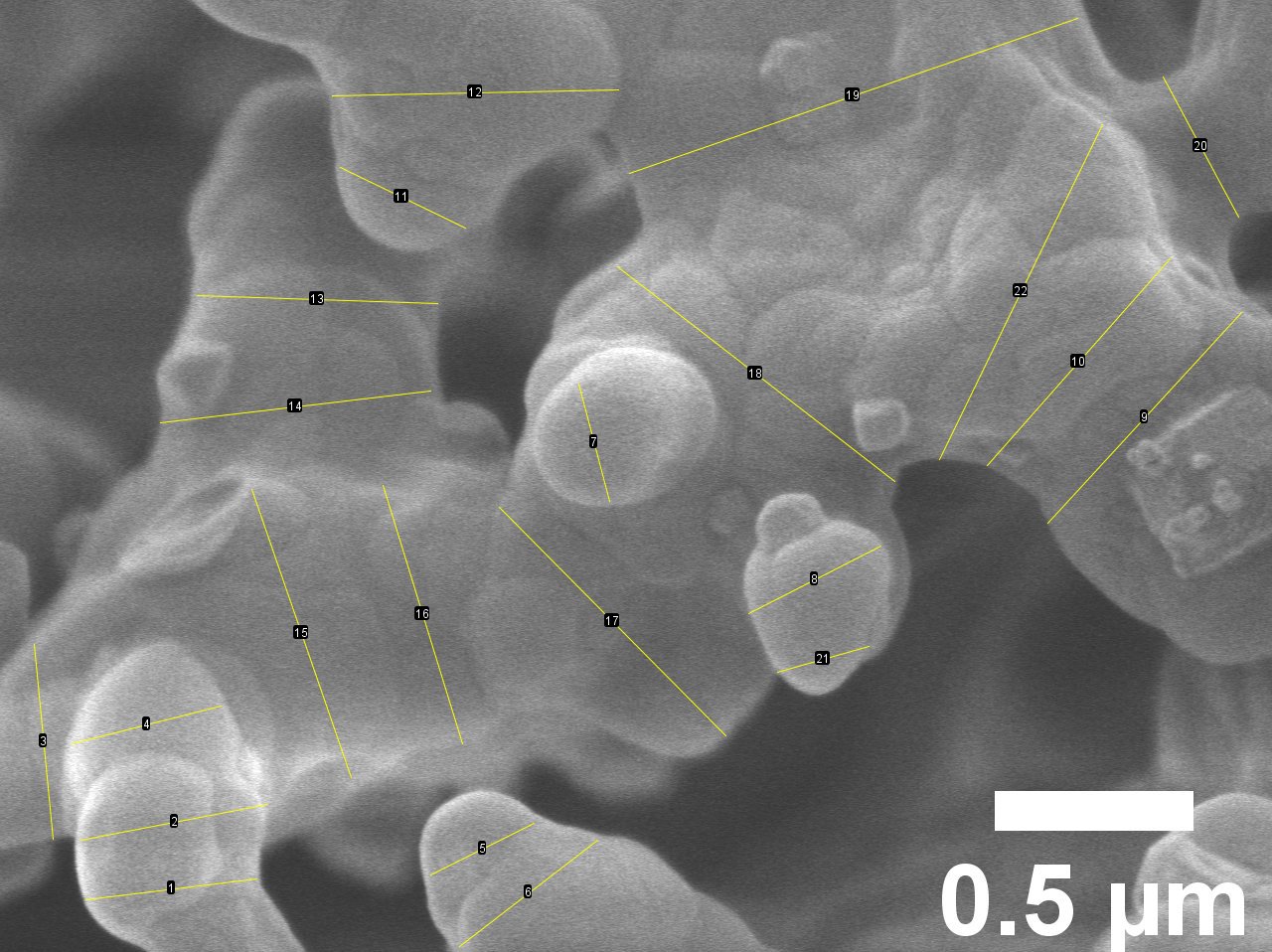


**Supplementary Figure S12** The average particle size of 20Mn-Ti-NW/SG.

**Supplementary Table S11** The average particle size of 20Mn-Ti-NW/SG.

| No. | Length (μm) | No. | Length (μm) | No. | Length (μm) |
| --- | --- | --- | --- | --- | --- |
| 1 | 0.433 | 9 | 0.720 | 17 | 0.810 |
| 2 | 0.479 | 10 | 0.694 | 18 | 0.882 |
| 3 | 0.492 | 11 | 0.349 | 19 | 1.193 |
| 4 | 0.387 | 12 | 0.720 | 20 | 0.401 |
| 5 | 0.291 | 13 | 0.605 | 21 | 0.239 |
| 6 | 0.439 | 14 | 0.685 | 22 | 0.937 |
| 7 | 0.305 | 15 | 0.767 | **Avg.** | **0.585** |
| 8 | 0.374 | 16 | 0.679 |  |  |

**Supplementary Figure S13** Plots of N_2_-physisorption at standard temperature and pressure (STP): a) CS and SG, b) MnTiO_3_-NW/CS, c) MnTiO_3_-NW/SG, and d) Mn-Ti-NW/SG catalysts. Each inset shows the corresponding pore size distribution plot.

**Supplementary Table S12** FE-SEM and EDX images of catalysts.

| **Catalyst** | **SEM** | **EDX and weight percent elemental** | | | | |
| --- | --- | --- | --- | --- | --- | --- |
|  |  | **Mn** | **Ti** | **Na** | **W** | **Si** |
| NW/CS | 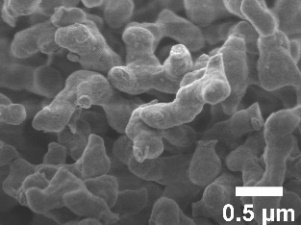 |  |  | 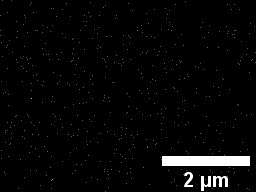  0.30% | 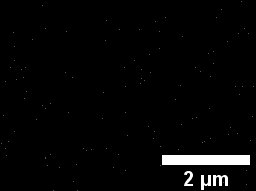  2.80% | 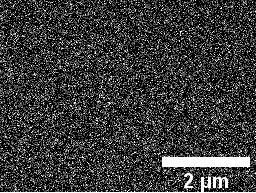  39.34% |
| 5MnTiO_3_-NW/CS | 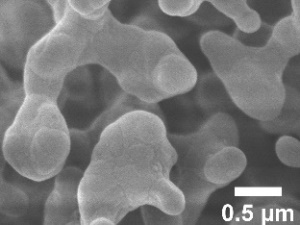 | 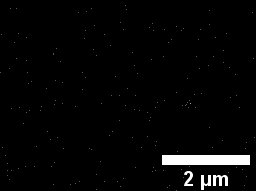  0.01% | 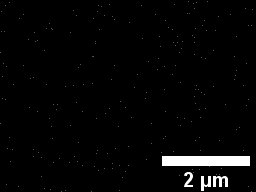  0.01% | 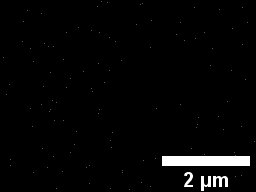  0.10% | 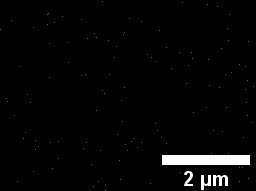  2.15% | 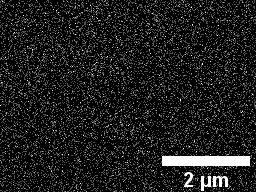  45.46% |
| 20MnTiO_3_-NW/CS | 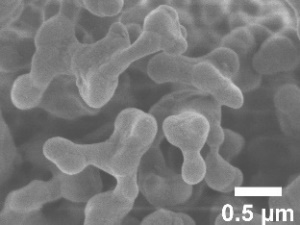 | 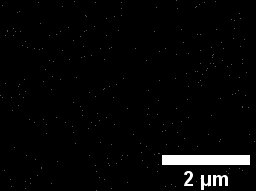  0.34% | 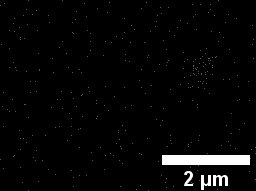  0.28% | 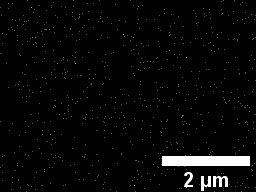  0.14% | 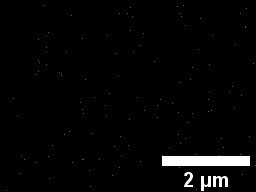  3.11% | 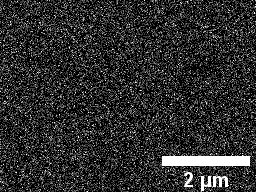  27.81% |
| NW/SG | 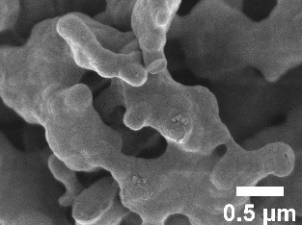 |  |  | 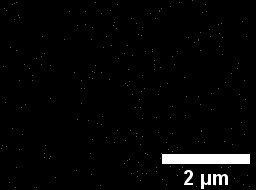  0.01% | 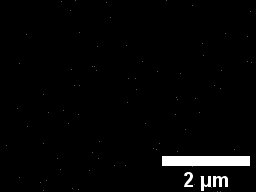  2.17% | 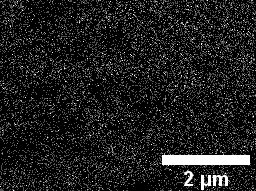  27.81% |

**Supplementary Table S12** FE-SEM and EDX images of catalysts. (Continued)

| **Catalyst** | **SEM** | **EDX and weight percent elemental** | | | | |
| --- | --- | --- | --- | --- | --- | --- |
|  |  | **Mn** | **Ti** | **Na** | **W** | **Si** |
| 10MnTiO_3_-NW/SG | 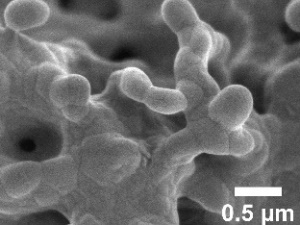 | 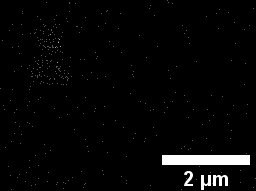  1.59% | 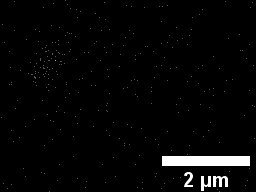  0.52% | 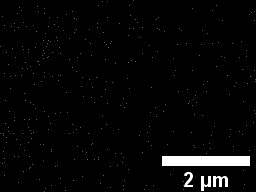  0.02% | 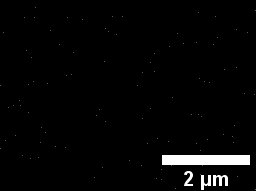  1.89% | 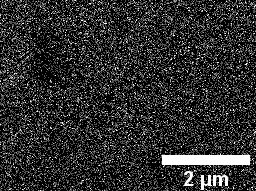  39.72% |
| 20MnTiO_3_-NW/SG | 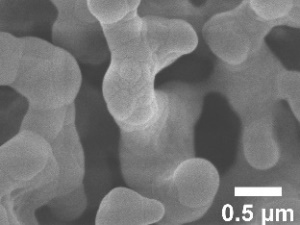 | 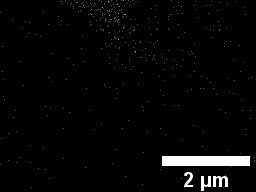  1.56% | 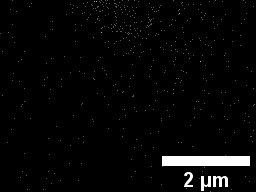  0.93% | 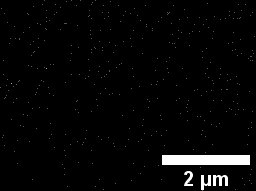  0.15% | 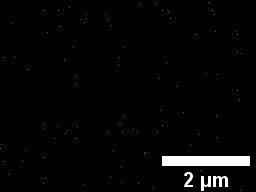  0.01% | 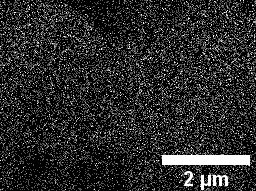  25.84% |
| 5Mn-Ti-NW/SG | 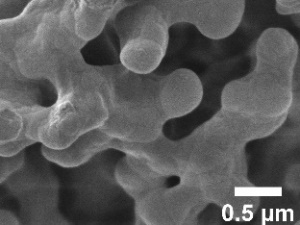 | 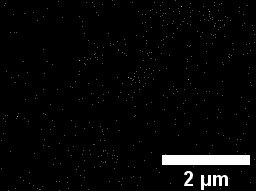  1.23% | 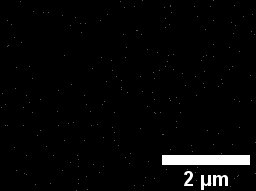  0.01% | 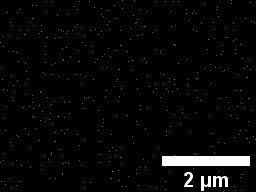  0.01% | 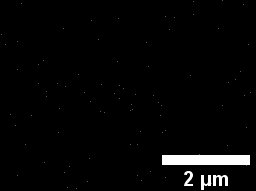  1.22% | 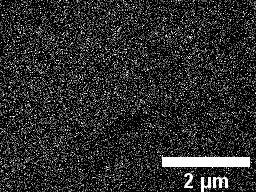  19.79% |
| 20Mn-Ti-NW/SG | 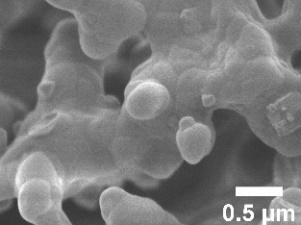 | 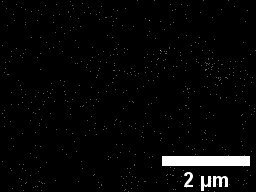  2.81% | 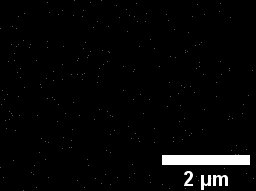  0.01% | 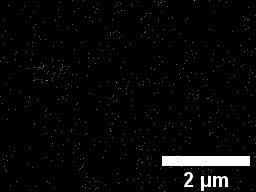  0.53% | 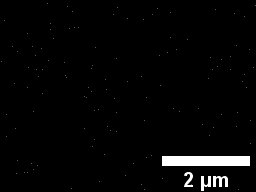  2.81% | 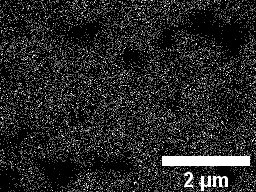  28.15% |

**Supplementary Table S12** FE-SEM and EDX images of catalysts. (Continued)

| **Catalyst** | **SEM** | **EDX and weight percent elemental** | | | | |
| --- | --- | --- | --- | --- | --- | --- |
|  |  | **Mn** | **Ti** | **Na** | **W** | **Si** |
| Used MnTiO_3_-NW/CS | 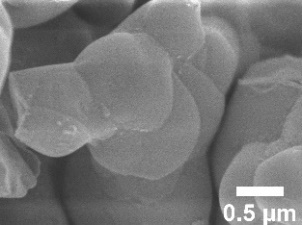 | 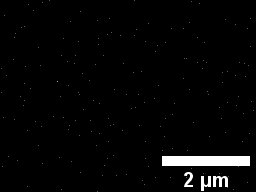  0.15% | 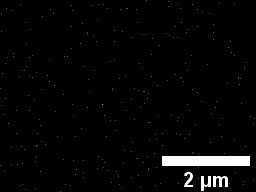  0.16% | 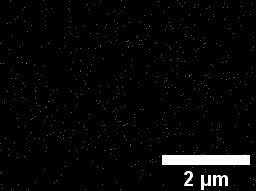  0.23% | 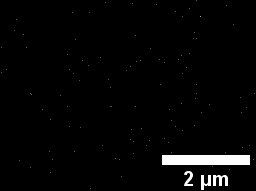  1.66% | 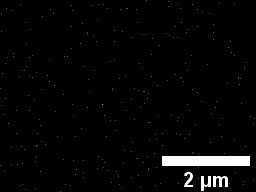  35.15% |
| Used MnTiO_3_-NW/SG | 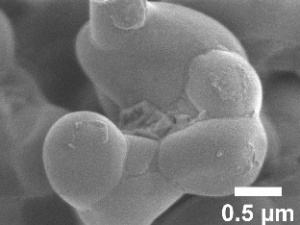 | 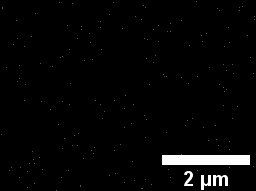  0.01% | 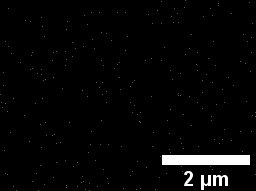  0.01% | 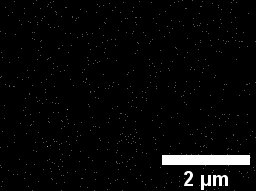  0.30% | 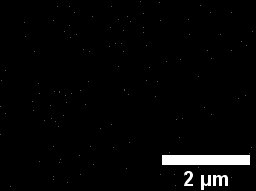  1.44% | 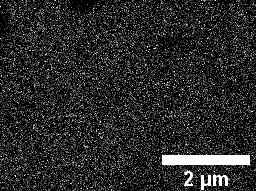  24.64% |
| Used Mn-Ti-NW/SG | 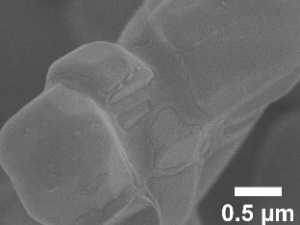 | 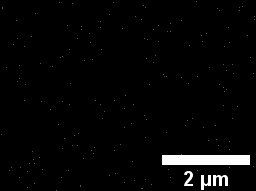  4.26% | 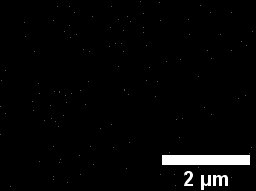  0.01% | 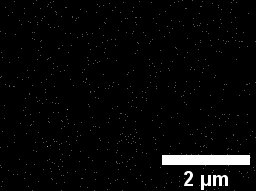  0.30% | 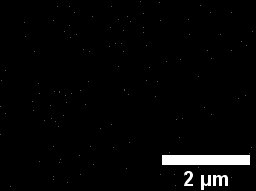  1.96% | 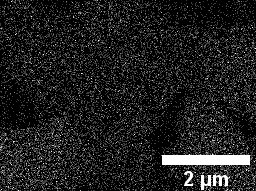  24.44% |

**Supplementary Figure S14**  FTIR spectra of CS, Na/CS, NW/CS, and MnTiO_3_-NW/CS.

**Supplementary Table S13** Observed XPS binding energies of Na 1s, W 4f, Si 2p, Ti 2p, and Mn 2p.

| Catalyst | Binding energy (eV) | | | | | | | | |
| --- | --- | --- | --- | --- | --- | --- | --- | --- | --- |
|  | Na 1s | W 4f | | Si 2p | Mn 2p | | Ti 2p | |  |
|  |  | 4f_5/2_ | 4f_7/2_ |  | 2p_1/2_ | 2p_3/2_ | 2p_1/2_ | 2p_3/2_ |  |
| NW/CS | 1071.3 | 37.6 | 35.4 | 103.5 | - | - | - | - |  |
| MnTiO_3_-NW/CS | 1071.3 | 37.6 | 35.4 | 103.4 | 655.4,  654.1,  652.8 | 643.9,  642.4,  641.3 | 464.74 | 459.1 |  |
| NW/SG | 1071.6 | 37.8 | 35.7 | 103.7 | - | - | - | - |  |
| MnTiO_3_-NW/SG | 1071.6 | 37.8 | 35.7 | 103.1 | 655.7,  654.1,  652.8 | 644.9,  642.1,  641.4 | 464.8 | 459.2 |  |
| Mn-Ti-NW/SG | 1071.3 | 37.6 | 35.5 | 103.4 | 655.1,  654.0,  652.8 | 643.3,  642.4,  641.4 | 464.0 | 458.5 |  |

**
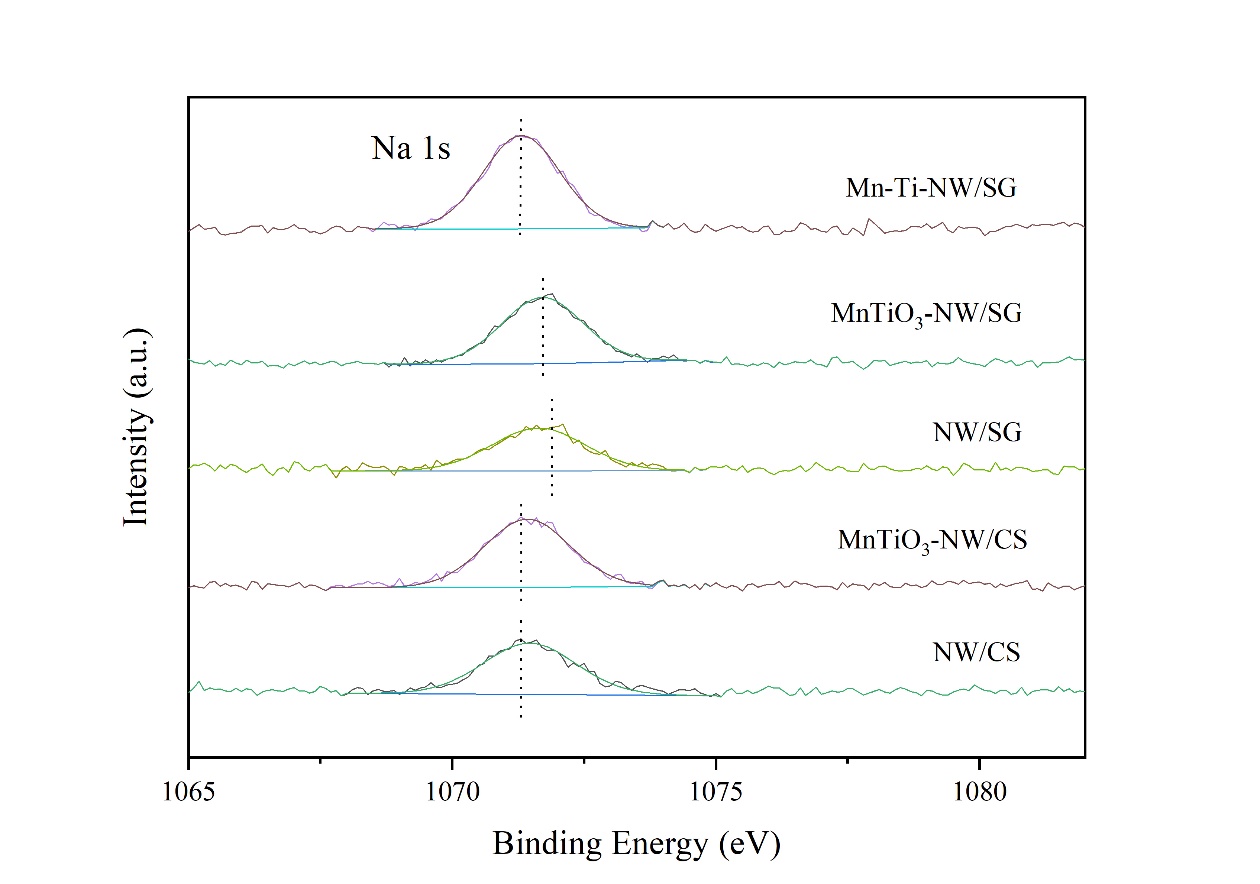
**

**Supplementary Figure S15** XPS Na 1s spectra of catalysts.

**
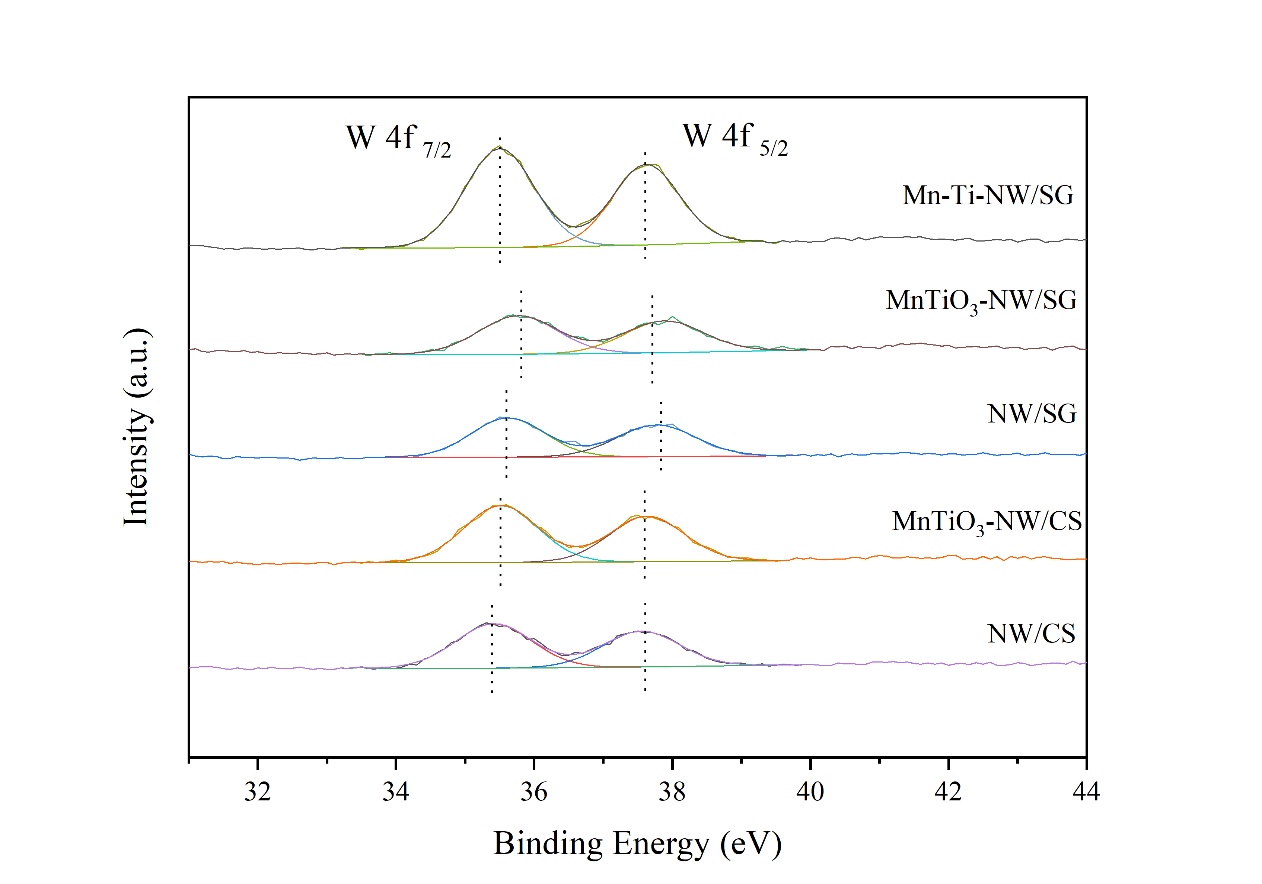
**

**Supplementary Figure S16** XPS W 4f spectra of catalysts.

**
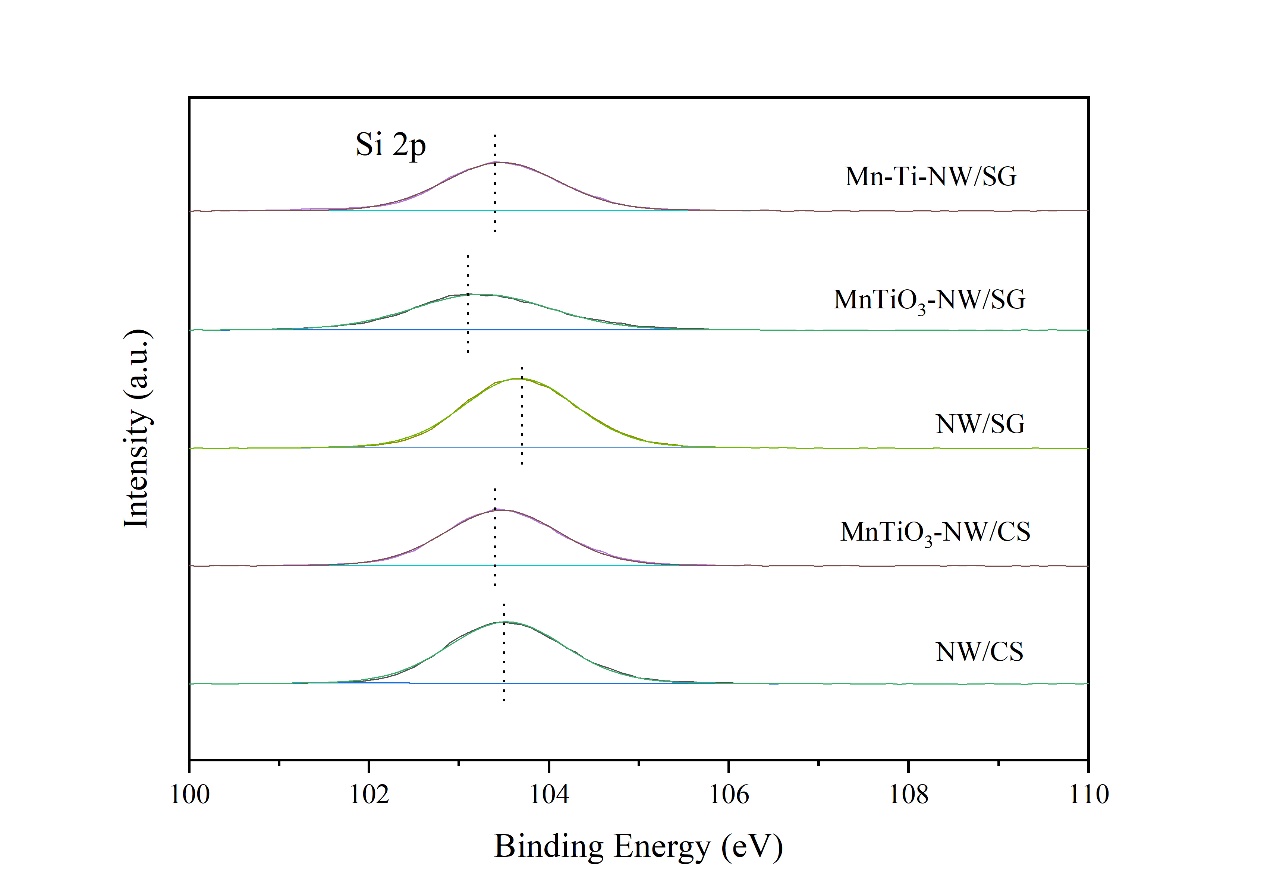
**

**Supplementary Figure S17** XPS Si 2p spectra of catalysts.

**
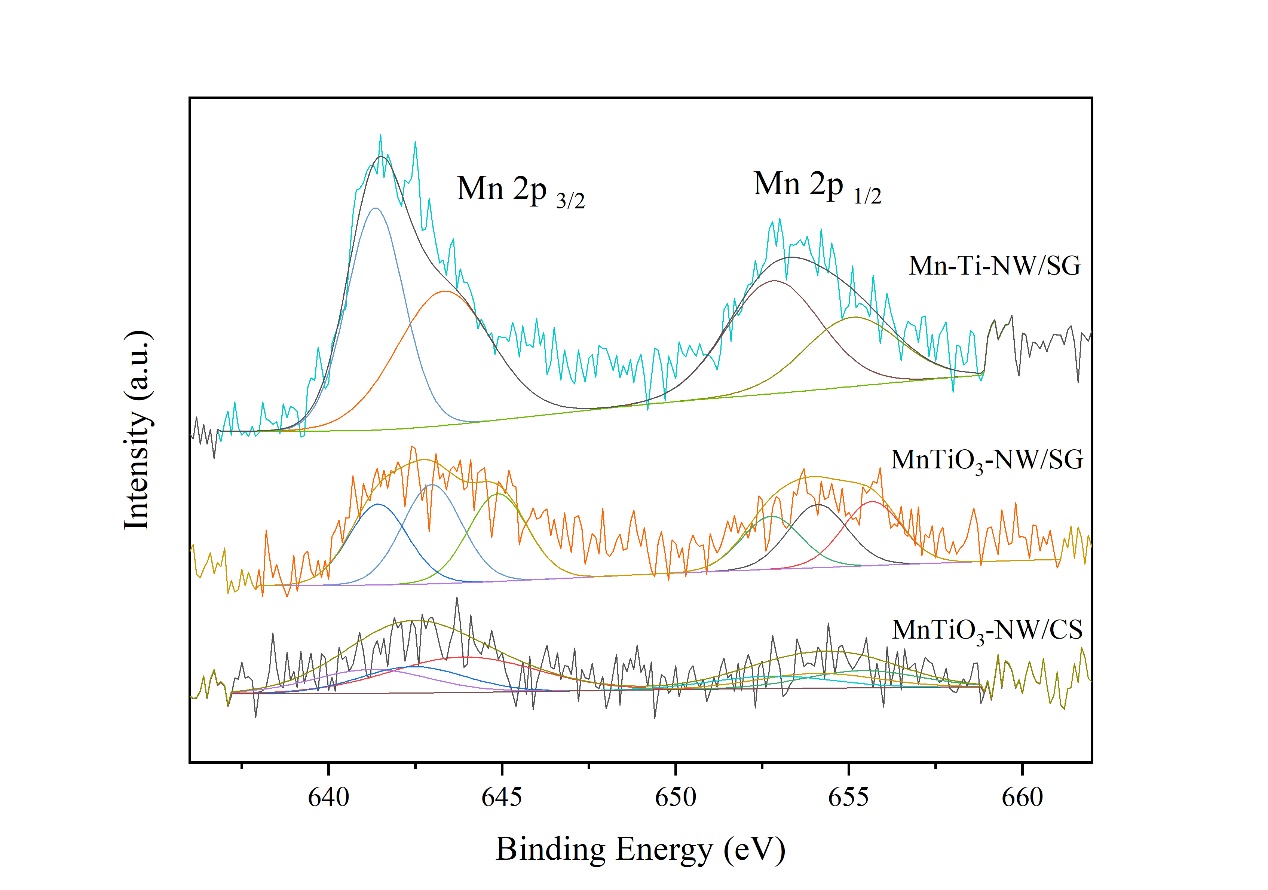
**

**Supplementary Figure S18** XPS Mn 2p spectra of catalysts.


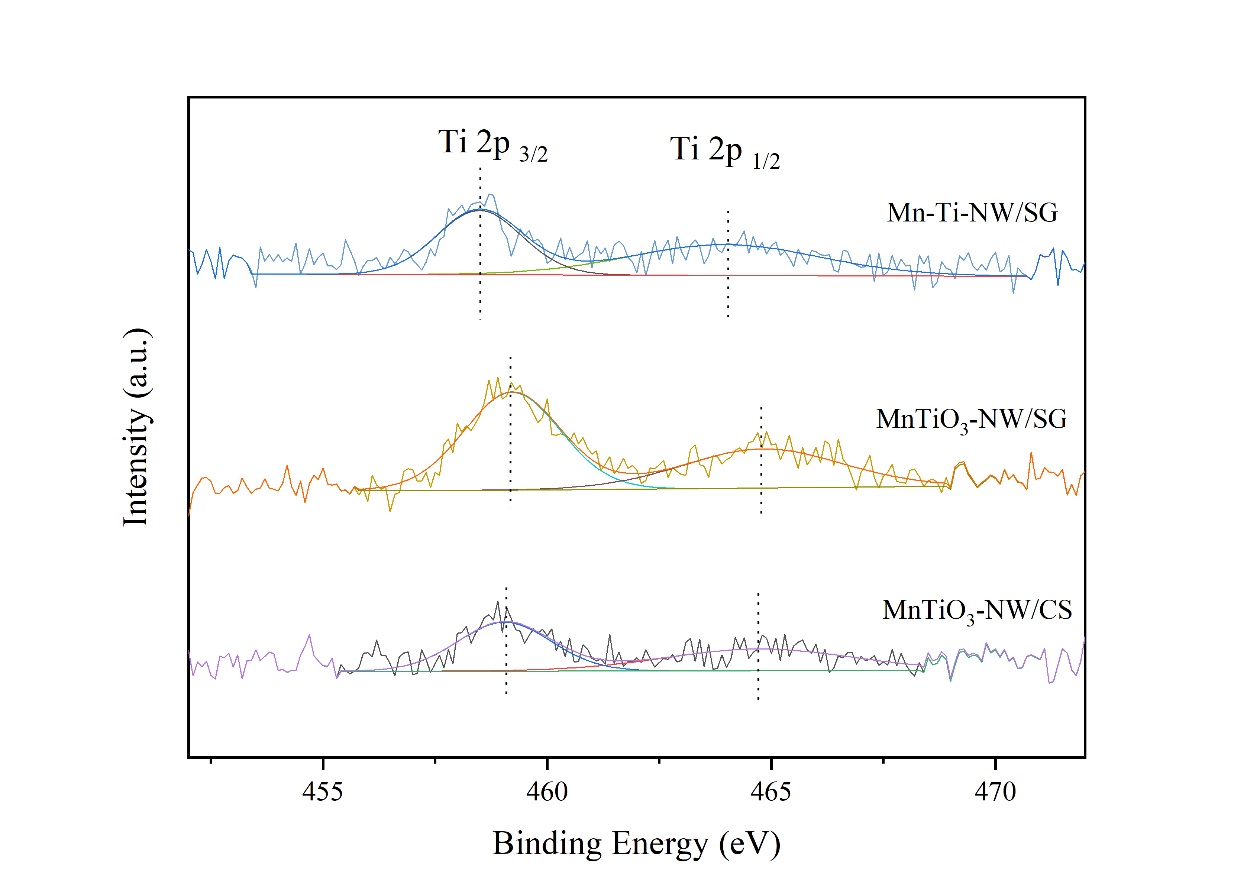


**Supplementary Figure S19** XPS Ti 2p spectra of the catalysts.

**Supplementary Figure S20** Catalytic performance of Mn-Ti-NW/CS.

**
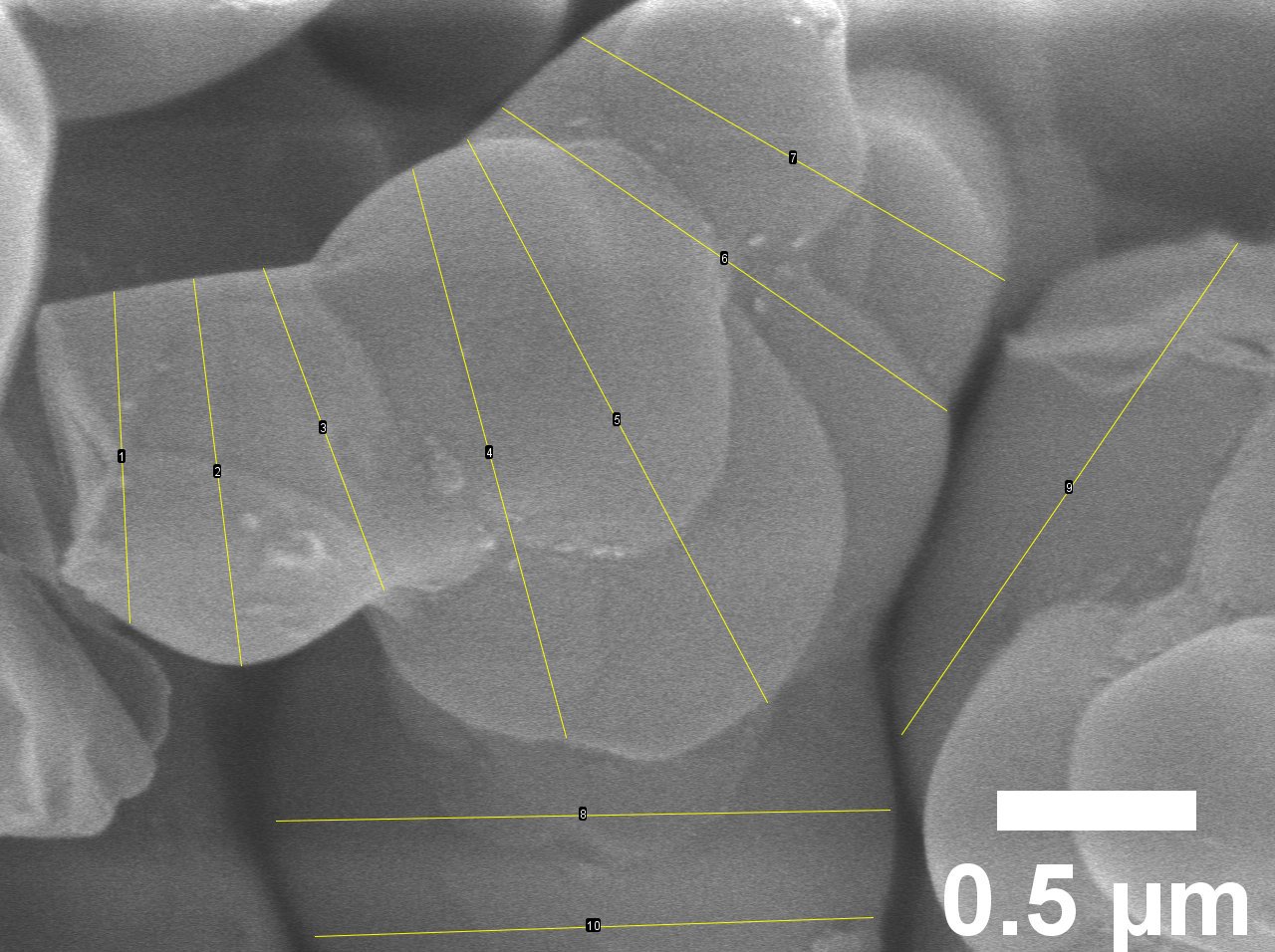
**

**Supplementary Figure S21** The average particle size of Used MnTiO_3_-NW/CS.

**Supplementary Table S14** The average particle size of Used MnTiO_3_-NW/CS.

| No. | Length (μm) |
| --- | --- |
| 1 | 0.831 |
| 2 | 0.977 |
| 3 | 0.862 |
| 4 | 1.476 |
| 5 | 1.600 |
| 6 | 1.349 |
| 7 | 1.223 |
| 8 | 1.540 |
| 9 | 1.493 |
| 10 | 1.401 |
| Avg. | **1.275** |

**
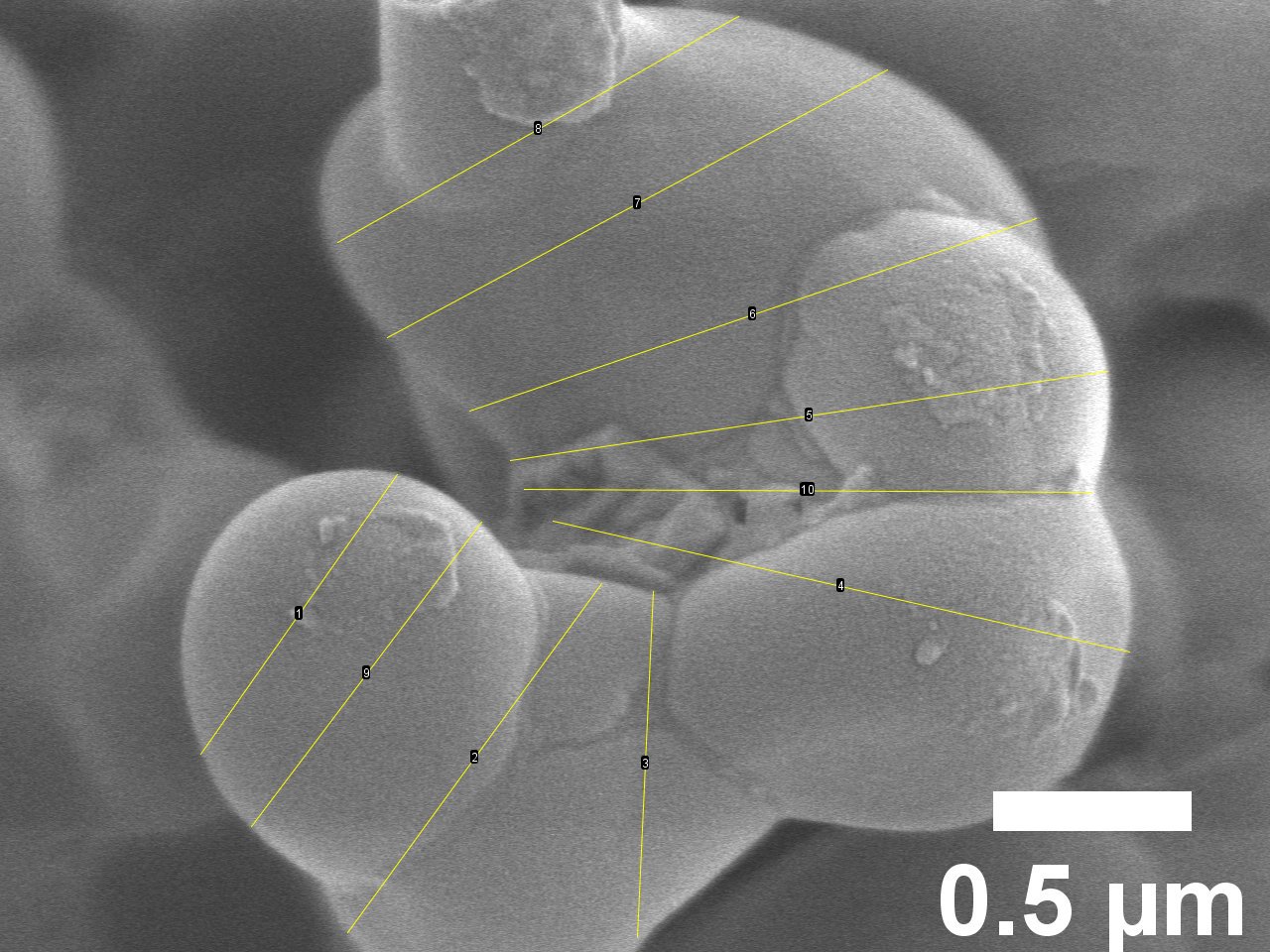
**

**Supplementary Figure S22** The average particle size of Used MnTiO_3_-NW/SG.

**Supplementary Table S15** The average particle size of Used MnTiO_3_-NW/SG.

| No. | Length (μm) |
| --- | --- |
| 1 | 0.861 |
| 2 | 1.088 |
| 3 | 0.871 |
| 4 | 1.490 |
| 5 | 1.522 |
| 6 | 1.508 |
| 7 | 1.429 |
| 8 | 1.160 |
| 9 | 0.962 |
| 10 | 1.430 |
| Avg. | **1.232** |


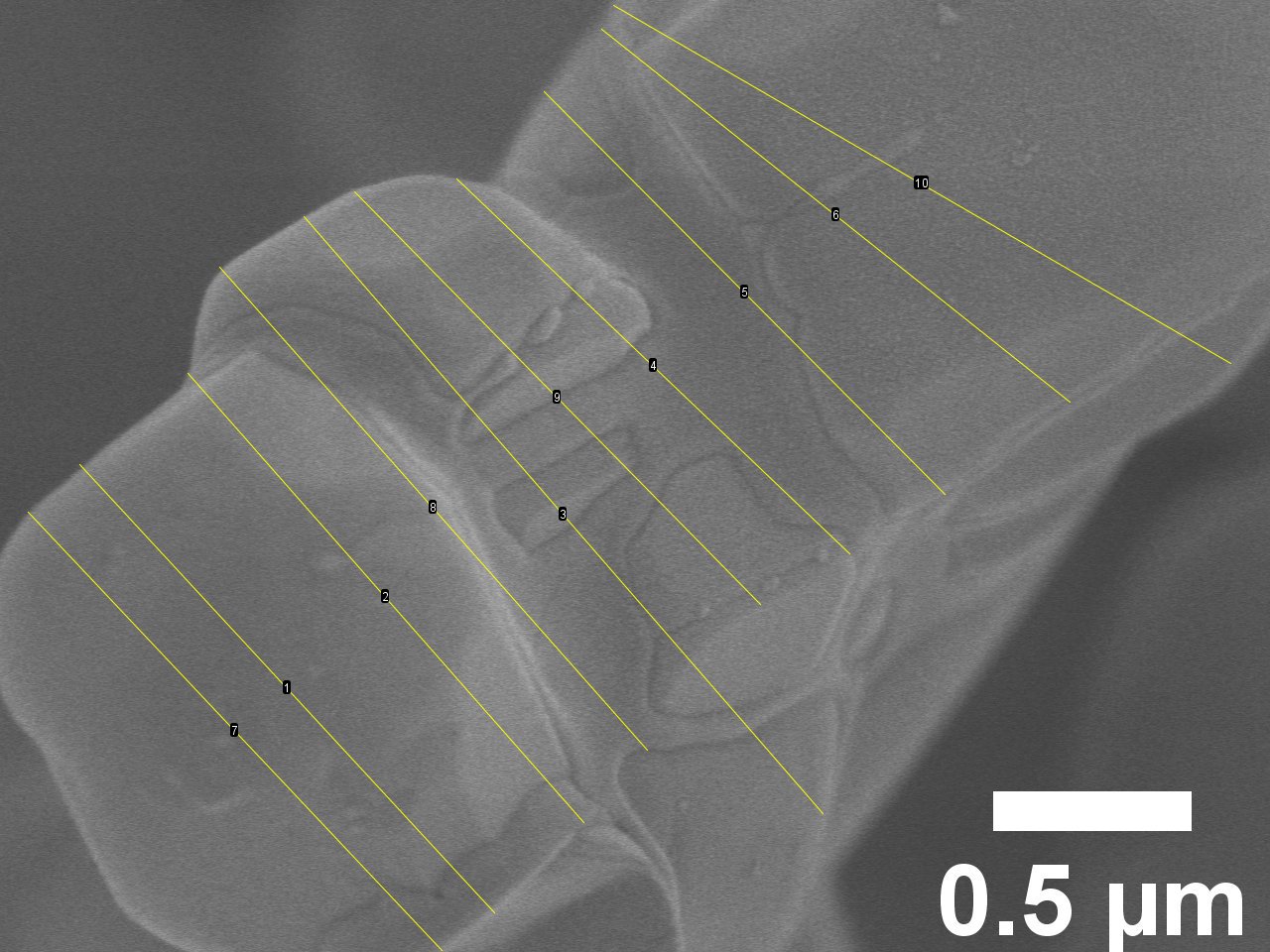


**Supplementary Figure S23** The average particle size of Used Mn-Ti-NW/SG.

**Supplementary Table S16** The average particle size of Used Mn-Ti-NW/SG.

| No. | Length (μm) |
| --- | --- |
| 1 | 1.541 |
| 2 | 1.509 |
| 3 | 1.994 |
| 4 | 1.369 |
| 5 | 1.432 |
| 6 | 1.509 |
| 7 | 1.519 |
| 8 | 1.626 |
| 9 | 1.458 |
| 10 | 1.798 |
| Avg. | **1.575** |

The mechanisms of OCM reaction, methane oxidation, and dehydrogenation are shown in equations (S1)–(S14).

.

$\text{2CH}\text{4}\text{ + }\text{0}\text{.}\text{5O}\text{2}\text{ }\text{ }\to\text{ C}\text{2}\text{H}\text{6}\text{ + }\text{H}\text{2}\text{O}$ (S1)

$\text{C}\text{2}\text{H}\text{6}\text{ + }\text{0}\text{.}\text{5O}\text{2}\text{ }\to\text{ C}\text{2}\text{H}\text{4}\text{ + }\text{H}\text{2}\text{O}$ (S2)

$\text{CH}\text{4}\text{ + }\text{O}\text{2}\text{ }\text{ }\to\text{ CO}\text{2}\text{ + }\text{2H}\text{2}\text{O}$ (S3)

$\text{CH}\text{4}\text{ + }\text{O}\text{2}\text{ }\text{ }\to\text{ CO}\text{2}\text{ + }\text{2H}\text{2}\text{O}\text{ + }\text{H}\text{2}$ (S4)

$\text{CO}\text{ + }\text{0}\text{.}\text{5O}\text{2}\text{ }\text{ }\to\text{ CO}\text{2}$ (S5)

$\text{C}\text{2}\text{H}\text{4}\text{ + }\text{2O}\text{2}\text{ }\text{ }\to\text{ 2CO}\text{ + }\text{2H}\text{2}\text{O}$ (S6)

$\text{C}\text{2}\text{H}\text{6}\text{ }\text{ }\to\text{ C}\text{2}\text{H}\text{4}\text{ + }\text{H}\text{2}$ (S7)

$\text{C}\text{2}\text{H}\text{4}\text{ + }\text{2H}\text{2}\text{O}\text{ }\to\text{ 2CO}\text{ + }\text{4H}\text{2}$ (S8)

$\text{CO}\text{ + }\text{H}\text{2}\text{O}\text{ }\to\text{ }\text{CO}\text{2}\text{+ }\text{H}\text{2}$ (S9)

$\text{CO}\text{2}\text{ + }\text{H}\text{2}\text{ }\to\text{ CO}\text{+ }\text{H}\text{2}\text{O}$ (S10)

$\text{C}_{\text{3}}\text{H}_{\text{6}}\text{ }\text{+}\text{ }\text{4.5O}_{\text{2}}\text{ }\text{→} \text{3CO}_{\text{2}}\text{ }\text{+}\text{ }\text{3H}_{\text{2}}\text{O}$ (S11)

$\text{C}_{\text{3}}\text{H}_{\text{6}}\text{ }\text{+}\text{ }\text{H}_{\text{2}}\text{ }\text{→} \text{C}_{\text{3}}\text{H}_{\text{8}}$ (S12)

$\text{C}_{\text{3}}\text{H}_{\text{8}}\text{ }\text{+}\text{ }\text{0.5}\text{O}_{\text{2}}\text{ }\text{→} \text{C}_{\text{3}}\text{H}_{\text{6}}\text{ }\text{+}\text{ }\text{H}_{\text{2}}\text{O}$ (S13)

$\text{C}_{\text{3}}\text{H}_{\text{8}}\text{ }\text{+}{\text{ }\text{5O}}_{\text{2}}\text{ }\text{→}{\text{ }\text{3CO}}_{\text{2}}\text{ }\text{+}{\text{ }\text{4H}}_{\text{2}}\text{O}$ (S14)

[Reference: B. Beck et al. / Catalysis Today 228 (2014) 212–218]

**Supplementary Table S17** A survey of catalysts reported in literature.

| No. | Catalyst | Reaction temperature (˚C) | C_2+_ yield  (%) | | C_2+_ selectivity  (%) | | CH_4_ conversion  (%) | | Ref. |
| --- | --- | --- | --- | --- | --- | --- | --- | --- | --- |
| 1 | Na_2_WO_4_/SiO_2_ | 800 | 9.1 |  | 74.3 |  | 12.3 |  | ^1^ |
| 2 | Mn-Na_2_WO_4_/SiO_2_ | 800 | 23.9 |  | 64.9 |  | 36.8 |  | ^1^ |
| 3 | Mn/Na_2_WO_4_/SiO_2_ | 850 | 26.4 |  | 80.0 |  | 33.0 |  | ^2^ |
| 4 | Na_2_WO_4_/SiO_2_ | 850 | 22.9 |  | 52.0 |  | 44.0 |  | ^2^ |
| 5 | Na_2_WO_4_/SiO_2_ | 775 | 7.0 |  | 63.0 |  | 11.0 |  | ^3^ |
| 6 | V/Na_2_WO_4_/SiO_2_ | 775 | 1.2 |  | 12.0 |  | 10.0 |  | ^3^ |
| 7 | Cr/Na_2_WO_4_/SiO_2_ | 775 | 2.4 |  | 24.0 |  | 10.0 |  | ^3^ |
| 8 | Mn/Na_2_WO_4_/SiO_2_ | 775 | 16.0 |  | 80.0 |  | 20.0 |  | ^3^ |
| 9 | Fe/Na_2_WO_4_/SiO_2_ | 775 | 9.0 |  | 60.0 |  | 15.0 |  | ^3^ |
| 10 | CO/Na_2_WO_4_/SiO_2_ | 775 | 11.0 |  | 68.0 |  | 16.0 |  | ^3^ |
| 11 | Zn/Na_2_WO_4_/SiO_2_ | 775 | 6.0 |  | 63.0 |  | 9.0 |  | ^3^ |
| 12 | Mn-Na_2_WO_4_/SiO_2_ | 850 | 17.0 |  | 75.0 |  | 22.0 |  | ^4^ |
| 13 | Na_2_WO_4_/Mn/SiO_2_ | 850 | 17.2 |  | 54.0 |  | 32.0 |  | ^5^ |
| 14 | W-Mn/SiO_2_ | 820 | 20.7 |  | 68.4 |  | 30.3 |  | ^6^ |
| 15 | W-Li/SiO_2_ | 800 | 6.0 |  | 52.0 |  | 10.0 |  | ^7^ |
| 16 | Mn-Li/SiO_2_ | 800 | 6.0 |  | 40.0 |  | 15.0 |  | ^7^ |
| 17 | Mn-W/SiO_2_ | 800 | 6.0 |  | 40.0 |  | 15.0 |  | ^7^ |
| 18 | Mn-0.1Li-W/SiO_2_ | 800 | 11.0 |  | 58.0 |  | 18.0 |  | ^7^ |
| 19 | Mn-0.25Li-W/SiO_2_ | 800 | 15.0 |  | 78.0 |  | 18.0 |  | ^7^ |
| 20 | W-Li-W/SiO_2_ | 800 | 15.0 |  | 79.0 |  | 19.0 |  | ^7^ |
| 21 | Na_2_WO_4_/Mn/SiO_2_ | 800 | 11.2 |  | 27.4 |  | 41.0 |  | ^8^ |
| 22 | La/Na_2_WO_4_/Mn/SiO_2_ | 800 | 10.6 |  | 25.4 |  | 41.7 |  | ^8^ |
| 23 | Na-W-Mn/SiO_2_ | 850 | 29.0 |  | 69.0 |  | 42.0 |  | ^9^ |
| 24 | Na_2_WO_4_/SiO_2_ | 800 | 15.0 |  | 74.0 |  | 20.0 |  | ^10^ |
| 25 | W/SiO_2_ | 800 | 6.0 |  | 54.0 |  | 11.0 |  | ^10^ |
| 26 | Mn/SiO_2_ | 800 | 9.0 |  | 43.0 |  | 19.0 |  | ^10^ |
| 27 | W-Na-Mn/SiO_2_ | 775 | 18.3 |  | 39.6 |  | 46.1 |  | ^11^ |
| 28 | Mo-Na-Mn/SiO_2_ | 775 | 12.5 |  | 37.2 |  | 33.6 |  | ^11^ |
| 29 | Nb-Na-Mn/SiO_2_ | 775 | 13.0 |  | 33.2 |  | 39.3 |  | ^11^ |
| 30 | V-Na-Mn/SiO_2_ | 775 | 2.7 |  | 8.0 |  | 33.5 |  | ^11^ |
| 31 | Cr-Na-Mn/SiO_2_ | 775 | 4.6 |  | 11.5 |  | 40.1 |  | ^11^ |
| 32 | Ce-Mn/Na_2_WO_4_/SiO_2_ | 840 | 21.1 |  | 62.4 |  | 33.9 |  | ^12^ |
| 33 | Na-W-Mn/SiO_2_ | 800 | 19.1 |  | 63.2 |  | 30.2 |  | ^13^ |
| 34 | Mn-Na_2_WO_4_/SiO_2_ | 825 | 19.1 |  | 72.2 |  | 26.5 |  | ^14^ |
| 35 | Mn/SiO_2_ | 800 | 0.6 |  | 14.4 |  | 5.7 |  | ^15^ |
| 36 | Na_2_WO_4_/SiO_2_ | 800 | 2.9 |  | 69.0 |  | 4.8 |  | ^15^ |
| 37 | Mn-Na_2_WO_4_/SiO_2_ | 800 | 18.5 |  | 73.3 |  | 28.5 |  | ^15^ |
| 38 | NaCl-Mn-Na_2_WO_4_/SiO_2_ | 750 | 34.6 |  | 62.9 |  | 55.0 |  | ^16^ |
| 39 | (NaCl+KCl)-Mn-Na_2_WO_4_/SiO_2_ | 750 | 31.6 |  | 70.0 |  | 45.1 |  | ^16^ |

**Supplementary Table S17** A survey of catalysts reported in literature. (Continued)

| No. | Catalyst | Reaction temperature (˚C) | C_2+_ yield  (%) | | C_2+_ selectivity  (%) | | CH_4_ conversion  (%) | | Ref. |
| --- | --- | --- | --- | --- | --- | --- | --- | --- | --- |
| 40 | KCl-Mn-Na_2_WO_4_/SiO_2_ | 750 | 27.0 |  | 75.3 |  | 35.9 |  | ^16^ |
| 41 | CsCl-Mn-Na_2_WO_4_/SiO_2_ | 750 | 22.6 |  | 74.9 |  | 30.1 |  | ^16^ |
| 42 | LiCl-Mn-Na_2_WO_4_/SiO_2_ | 750 | 11.3 |  | 80.2 |  | 14.1 |  | ^16^ |
| 43 | Mn-Na_2_WO_4_/SiO_2_ | 750 | 5.2 |  | 63.4 |  | 8.2 |  | ^16^ |
| 44 | Mn_x_O_y_-Na_2_WO_4_/D11-10 | 750 | 3.6 |  | 52.9 |  | 6.7 |  | ^17^ |
| 45 | Mn_x_O_y_-Na_2_WO_4_/SiO_2_ (grade 923) | 750 | 1.3 |  | 63.6 |  | 2.0 |  | ^17^ |
| 46 | Mn_x_O_y_-Na_2_WO_4_/SiO_2_ (fumed) | 750 | 4.5 |  | 61.3 |  | 7.4 |  | ^17^ |
| 47 | Mn_x_O_y_-Na_2_WO_4_/Aerosil TT 600 | 750 | 4.5 |  | 60.7 |  | 7.3 |  | ^17^ |
| 48 | Mn_x_O_y_-Na_2_WO_4_/Aeroperl R 806/30 | 750 | 4.9 |  | 68.9 |  | 7.1 |  | ^17^ |
| 49 | Mn_x_O_y_-Na_2_WO_4_/Aerosil OX 50 | 750 | 3.5 |  | 55.4 |  | 6.4 |  | ^17^ |
| 50 | Mn_x_O_y_-Na_2_WO_4_/Aerosil 380 | 750 | 4.2 |  | 62.6 |  | 6.6 |  | ^17^ |
| 51 | Mn_x_O_y_-Na_2_WO_4_/Aerosil 300 | 750 | 3.3 |  | 57.4 |  | 5.9 |  | ^17^ |
| 52 | Mn_x_O_y_-Na_2_WO_4_/Sipernat D10 | 750 | 3.5 |  | 80.3 |  | 4.4 |  | ^17^ |
| 53 | Mn_x_O_y_-Na_2_WO_4_/Sipernat 310 | 750 | 5.4 |  | 75.8 |  | 7.0 |  | ^17^ |
| 54 | Mn_x_O_y_-Na_2_WO_4_/SBA-15 | 750 | 10.4 |  | 73.4 |  | 14.1 |  | ^17^ |
| 55 | Na_2_WO_4_/Mn/SiO_2_ | 800 | 2.5 |  | 67.6 |  | 3.2 |  | ^18^ |
| 56 | Na_2_WO_4_-Mn/SiO_2_ | 800 | 19.6 |  | 66.4 |  | 29.5 |  | ^18^ |
| 57 | TiO_2_-Mn_2_O_3_-Na_2_WO_4_/SiO_2_ | 700 | 14.0 |  | 70.0 |  | 20.0 |  | ^19^ |
| 58 | TiO_2_-Mn_2_O_3_-Na_2_WO_4_/SiO_2_ | 720 | 19.7 |  | 76.0 |  | 26.0 |  | ^20^ |
| 59 | Mn_2_O_3_-TiO_2_-Na_2_WO_4_/SiO_2_ | 650 | 13.6 |  | 62.0 |  | 22.0 |  | ^20^ |
| 60 | Na_2_WO_4_/Mn/SiO_2_ (silica gel) | 775 | 16.9 |  | 50.8 |  | 33.4 |  | ^21^ |
| 61 | Na_2_WO_4_/Mn/SiO_2_ (silica gel) | 800 | 17.1 |  | 51.0 |  | 33.5 |  | ^21^ |
| 62 | Na_2_WO_4_/Mn/SiO_2_ (fumed silica) | 800 | 16.5 |  | 52.7 |  | 33.3 |  | ^21^ |
| 63 | Mn/Na_2_WO_4_/SiO_2_ | 725 | 16.0 |  | 79.8 |  | 20.2 |  | ^22^ |
| 64 | Mn-Na-W/SiO_2_ | 750 | 16.0 |  | 64.0 |  | 25.0 |  | ^23^ |
| 65 | Al-Na_2_WO_4_/SiO_2_ | 800 | 3.2 |  | 37.0 |  | 7.69 |  | ^24^ |
| 66 | Li-Na_2_WO_4_/SiO_2_ | 800 | 4.6 |  | 47.8 |  | 9.56 |  | ^24^ |
| 67 | La-Na_2_WO_4_/SiO_2_ | 800 | 6.1 |  | 49.5 |  | 11.9 |  | ^24^ |
| 68 | Cu-Na_2_WO_4_/SiO_2_ | 800 | 7.5 |  | 22.3 |  | 33.8 |  | ^24^ |
| 69 | Cr-Na_2_WO_4_/SiO_2_ | 800 | 9.8 |  | 29.9 |  | 27.7 |  | ^24^ |
| 70 | Na_2_-WO_4_/SiO_2_ | 800 | 10.9 |  | 24.0 |  | 33.5 |  | ^24^ |
| 71 | Mg-Na_2_WO_4_/SiO_2_ | 800 | 12.5 |  | 53.2 |  | 21.6 |  | ^24^ |
| 72 | Ni-Na_2_WO_4_/SiO_2_ | 800 | 13.7 |  | 36.1 |  | 35.4 |  | ^24^ |
| 73 | Ce-Na_2_WO_4_/SiO_2_ | 800 | 15.4 |  | 32.9 |  | 42.1 |  | ^24^ |
| 74 | Zn-Na_2_WO_4_/SiO_2_ | 800 | 17.8 |  | 65.9 |  | 25.5 |  | ^24^ |
| 75 | Co-Na_2_WO_4_/SiO_2_ | 800 | 18.4 |  | 10.2 |  | 41.8 |  | ^24^ |
| 76 | Mn-Na_2_WO_4_/SiO_2_ | 800 | 19.5 |  | 41.1 |  | 45.4 |  | ^24^ |

**Supplementary Table S17** A survey of catalysts reported in literature. (Continued)

| No. | Catalyst | Reaction temperature (˚C) | C_2+_ yield  (%) | | C_2+_ selectivity  (%) | | CH_4_ conversion  (%) | | Ref. |
| --- | --- | --- | --- | --- | --- | --- | --- | --- | --- |
| 77 | Mn-W/SiO_2_ | 800 | 11.0 |  | 48.0 |  | 24.0 |  | ^24^ |
| 78 | Ce-W/SiO_2_ | 800 | 7.0 |  | 35.0 |  | 20.0 |  | ^24^ |
| 79 | Mn-Na/SiO_2_ | 800 | 14.0 |  | 44.0 |  | 31.0 |  | ^24^ |
| 80 | Na_2_WO_4_-Mn/SiO_2_ | 800 | 20.0 |  | 67.0 |  | 30.0 |  | ^24^ |
| 81 | Na_2_WO_4_-Ce/SiO_2_ | 800 | 20.0 |  | 74.0 |  | 27.0 |  | ^24^ |
| 82 | TiO_2_-Mn_2_O_3_‐Na_2_WO_4_/SiO_2_ | 700 | 16.7 |  | 73.0 |  | 23.0 |  | ^25^ |
| 83 | Na_2_WO_4_-TiO_2_/SiO_2_ | 700 | 4.9 |  | 71.7 |  | 6.8 |  | ^26^ |
| 84 | MnO_x_-Na_2_WO_4_/SiO_2_ | 770 | 12.4 |  | 70.4 |  | 17.6 |  | ^27^ |
| 85 | Nb-MnO_x_-Na_2_WO_4_/SiO_2_ | 770 | 15.2 |  | 68.2 |  | 22.3 |  | ^27^ |
| 86 | Ti-MnO_x_-Na_2_WO_4_/SiO_2_ | 770 | 11.2 |  | 70.4 |  | 15.9 |  | ^27^ |
| 87 | Sn-MnO_x_-Na_2_WO_4_/SiO_2_ | 770 | 7.5 |  | 69.5 |  | 10.8 |  | ^27^ |
| 88 | Ce-MnO_x_-Na_2_WO_4_/SiO_2_ | 770 | 12.7 |  | 66.7 |  | 19.0 |  | ^27^ |
| 89 | Fe-MnO_x_-Na_2_WO_4_/SiO_2_ | 770 | 15.8 |  | 65.5 |  | 24.1 |  | ^27^ |
| 90 | Ge-MnO_x_-Na_2_WO_4_/SiO_2_ | 770 | 16.4 |  | 70.7 |  | 23.2 |  | ^27^ |
| 91 | Mn_2_O_3_-Na_2_WO_4_/SiC | 800 | 20.5 |  | 54.5 |  | 37.5 |  | ^28^ |
| 92 | Mn_2_O_3_-Na_2_WO_4_/SiO_2_ | 700 | 23.5 |  | 60.5 |  | 39.7 |  | ^29^ |
| 93 | Na_2_WO_4_-Ti-Mn/SiO_2_ | 700 | 22.1 |  | 62.3 |  | 35.4 |  | ^30^ |
| 94 | Na_2_WO_4_-TiO_2_-MnO_x_-Sr_0.25_/SiO_2_ | 750 | 22.9 |  | 62.5 |  | 36.6 |  | ^31^ |

**Supplementary Table S18** Comparisons of OCM performance of catalysts having different initial specific surface area. Testing conditions: 50 mg catalyst, gas feed of CH_4_:O_2_:N_2_ = 3:1:4, reactor temperature = 700 °C, atmospheric pressure, total feed gas flow rate = 50 mL min^-1^ (GHSV = 30,588 h^-1^).

| Catalyst | Specific surface area of fumed SiO_2_ (m² g^-1^) | BET surface area of catalyst (m² g^-1^) | C_2+_ selectivity (%) | CH_4_ conversion (%) | C_2+_ yield (%) |
| --- | --- | --- | --- | --- | --- |
| 5MnTiO_3_-NW/CS | 85–115 | 3.20 | 58.1 | 35.5 | 20.6 |
| 5MnTiO_3_-NW/CS* | 350–420 | 2.86 | 57.0 | 34.3 | 19.6 |

**Supplementary Table S19** Catalytic performance of 20Mn-Ti-NW/SG catalysts over 24 h. Testing conditions: 50 mg catalyst, gas feed of CH_4_:O_2_:N_2_ = 3:1:4, reactor temperature = 700 °C, atmospheric pressure, total feed gas flow rate = 50 mL min^-1^ (GHSV = 30,588 h^-1^).

| Time (h) | C_2+_ selectivity (%) | CH_4_ conversion (%) | C_2+_ yield (%) | r_C2+_ ^*^ | CH_4_ balance (%)^**^ |
| --- | --- | --- | --- | --- | --- |
| 1 | 62.2 | 33.7 | 21.0 | 0.24 | 5.9 |
| 2 | 61.7 | 35.6 | 22.0 | 0.25 | 1.7 |
| 3 | 58.9 | 38.1 | 22.4 | 0.26 | 6.7 |
| 4 | 60.8 | 36.6 | 22.3 | 0.25 | 4.0 |
| 5 | 63.1 | 34.8 | 22.0 | 0.25 | 0.2 |
| 6 | 62.2 | 35.2 | 21.9 | 0.25 | 1.2 |
| 7 | 62.8 | 34.8 | 21.9 | 0.25 | 1.7 |
| 8 | 62.5 | 35.1 | 22.0 | 0.25 | 1.2 |
| 9 | 62.5 | 34.8 | 21.8 | 0.25 | 1.5 |
| 10 | 62.5 | 34.9 | 21.8 | 0.25 | 1.2 |
| 11 | 63.0 | 34.7 | 21.9 | 0.25 | 1.1 |
| 12 | 62.6 | 35.1 | 22.0 | 0.25 | 0.4 |
| 13 | 62.0 | 35.2 | 21.8 | 0.25 | 0.4 |
| 14 | 62.0 | 34.8 | 21.6 | 0.25 | 0.6 |
| 15 | 62.0 | 34.7 | 21.5 | 0.25 | 0.5 |
| 16 | 62.1 | 34.4 | 21.4 | 0.24 | 0.4 |
| 17 | 62.0 | 33.9 | 21.0 | 0.24 | 0.1 |
| 18 | 62.0 | 33.6 | 20.9 | 0.24 | 0.0 |
| 19 | 61.9 | 33.7 | 20.8 | 0.24 | 0.6 |
| 20 | 61.8 | 32.7 | 20.2 | 0.23 | 0.8 |
| 21 | 61.7 | 33.1 | 20.4 | 0.23 | 0.9 |
| 22 | 61.4 | 32.6 | 20.0 | 0.23 | 0.0 |
| 23 | 61.5 | 32.6 | 20.0 | 0.23 | 0.6 |
| 24 | 61.3 | 32.7 | 20.1 | 0.23 | 1.4 |

**^*^** unit of r_C2+_ = moles of C_2+_/[(total moles of MnTiO_3_ or (Mn+Ti) and Na_2_WO_4_)×h]

**^**^** CH_4_ balance (%) = {$\text{n}_{\text{CH}_{\text{4}} in}$ –$[\text{2}\left( \text{n}_{\text{C}_{\text{2}}\text{H}_{\text{4}}}\text{+ }\text{n}_{\text{C}_{\text{2}}\text{H}_{\text{6}}} \right)\text{+3}\left( \text{n}_{\text{C}_{\text{3}}\text{H}_{\text{6}}}\text{+ }\text{n}_{\text{C}_{\text{3}}\text{H}_{\text{8}}} \right)\text{+4}\left( \text{n}_{\text{C}_{\text{4}}\text{H}_{\text{10}}} \right)\text{+ }\text{n}_{\text{CO}}\text{+ }\text{n}_{\text{CO}_{\text{2}}}+\text{n}_{\text{CH}_{\text{4}}out}]$}×100/$\text{n}_{\text{CH}_{\text{4}} in}$

**References**

1. Jiang, Z. C., Yu, C. J., Fang, X. P., Li, S. B. & Wang, H. L. Oxide/support interaction and surface reconstruction in the sodium tungstate (Na_2_WO_4_)/silica system. *J. Phys. Chem. A* **97**, 12870-12875 (1993).
2. Palermo, A., Vazquez, J. P. H., Tikhov, M. S. & Lambert, R. M. Critical influence of the amorphous silica-to-cristobalite phase transition on the performance of Mn/Na_2_WO_4_/SiO_2_ catalysts for the oxidative coupling of methane. *J. Catal.* **177**, 259-266 (1998).
3. Malekzadeh, A. et al. Correlation of electrical properties and performance of OCM MO_x_/Na_2_WO_4_/SiO_2_ catalysts. *Catal. Commun.* **2**, 241-247 (2001).
4. Ji, S. F. et al. The relationship between the structure and the performance of Na-W-Mn/SiO_2_ catalysts for the oxidative coupling of methane. *Appl. Catal. A Gen.* **225**, 271-284 (2002).
5. Ji, S. et al. Surface WO4 tetrahedron: The essence of the oxidative coupling of methane over M-W-Mn/SiO_2_ catalysts. *J. Catal.* **220**, 47-56 (2003).
6. Wang, J. et al. Comparative study on oxidation of methane to ethane and ethylene over Na_2_WO_4_-Mn/SiO_2_ catalysts prepared by different methods. *J. Mol. Catal. A Chem.* **245**, 272-277 (2006).
7. Malekzadeh, A., Khodadadi, A., Dalai, A. K. & Abedini, M. Oxidative coupling of methane over lithium doped (Mn+W)/SiO_2_ catalysts. *J. Nat. Gas Chem.* **16**, 121-129 (2007).
8. Wu, J., Zhang, H., Qin, S. & Hu, C. La-promoted Na_2_WO_4_/Mn/SiO_2_ catalysts for the oxidative conversion of methane simultaneously to ethylene and carbon monoxide. *Appl. Catal. A Gen.* **323**, 126-134 (2007).
9. Chua, Y. T., Mohamed, A. R. & Bhatia, S. Oxidative coupling of methane for the production of ethylene over sodium-tungsten-manganese-supported-silica catalyst (Na-W-Mn/SiO_2_). *Appl. Catal. A Gen.* **343**, 142-148 (2008).
10. Gholipour, Z., Malekzadeh, A., Hatami, R., Mortazavi, Y. & Khodadadi, A. Oxidative coupling of methane over (Na_2_WO_4_+Mn or Ce)/SiO_2_ catalysts: In situ measurement of electrical conductivity. *J. Nat. Gas Chem.* **19**, 35-42 (2010).
11. Mahmoodi, S., Ehsani, M. R. & Ghoreishi, S. M. Effect of promoter in the oxidative coupling of methane over synthesized Mn/SiO_2_ nanocatalysts via incipient wetness impregnation. *J. Ind. Eng. Chem.* **16**, 923-928 (2010).
12. Shahri, S. M. K. & Pour, A. N. Ce-promoted Mn/Na_2_WO_4_/SiO_2_ catalyst for oxidative coupling of methane at atmospheric pressure. *J. Nat. Gas Chem.* **19**, 47-53 (2010).
13. Lee, J. Y. et al. Scaled-up production of C_2_ hydrocarbons by the oxidative coupling of methane over pelletized Na_2_WO_4_/Mn/SiO_2_ catalysts: Observing hot spots for the selective process. *Fuel* **106**, 851-857 (2013).
14. Godini, H. R. et al. Sol–gel method for synthesis of Mn–Na_2_WO_4_/SiO_2_ catalyst for methane oxidative coupling. *Catal. Today* **236**, 12-22 (2014).
15. Elkins, T. W. & Hagelin-Weaver, H. E. Characterization of Mn-Na_2_WO_4_/SiO_2_ and Mn-Na_2_WO_4_/MgO catalysts for the oxidative coupling of methane. *Appl. Catal. A Gen.* **497**, 96-106 (2015).
16. Hiyoshi, N. & Ikeda, T. Oxidative coupling of methane over alkali chloride-Mn-Na_2_WO_4_/SiO_2_ catalysts: Promoting effect of molten alkali chloride. *Fuel Process. Technol.* **133**, 29-34 (2015).
17. Yildiz, M. et al. Silica material variation for the Mn_x_O_y_-Na_2_WO_4_/SiO_2_. *Appl. Catal. A Gen.* **525**, 168-179 (2016).
18. Fleischer, V., Steuer, R., Parishan, S. & Schomäcker, R. Investigation of the surface reaction network of the oxidative coupling of methane over Na_2_WO_4_/Mn/SiO_2_ catalyst by temperature programmed and dynamic experiments. *J. Catal.* **341**, 91-103 (2016).
19. Wang, P., Zhao, G., Liu, Y. & Lu, Y. TiO2-doped Mn_2_O_3_-Na_2_WO_4_/SiO_2_ catalyst for oxidative coupling of methane: Solution combustion synthesis and MnTiO_3_-dependent low-temperature activity improvement. *Appl. Catal. A Gen.* **544**, 77-83 (2017).
20. Wang, P., Zhao, G., Wang, Y. & Lu, Y. MnTiO_3_-driven low-temperature oxidative coupling of methane over TiO_2_-doped Mn_2_O_3_-Na_2_WO_4_/SiO_2_ catalyst. *Sci. Adv.* **3**, e1603180; doi:10.1126/sciadv.1603180 (2017).
21. Yunarti, R. T. et al. Oxidative coupling of methane using Mg/Ti-doped SiO_2_-supported Na_2_WO_4_/Mn catalysts. *ACS Sustain. Chem. Eng.* **5**, 3667-3674 (2017).
22. Uzunoglu, C., Leba, A. & Yildirim, R. Oxidative coupling of methane over Mn-Na_2_WO_4_ catalyst supported by monolithic SiO_2_. *Appl. Catal. A Gen.* **547**, 22-29 (2017).
23. Hayek, N. S., Lucas, N. S., Damouny, C. W. & Gazit, O. M. Critical surface parameters for the oxidative coupling of methane over the Mn-Na-W/SiO_2_ catalyst. *ACS Appl. Mater. Interfaces* **9**, 40404-40411 (2017).
24. Gu, S. et al. Effects of metal or metal oxide additives on oxidative coupling of methane using Na_2_WO_4_/SiO_2_ catalysts: Reducibility of metal additives to manipulate the catalytic activity. *Appl. Catal. A Gen.* **562**, 114-119 (2018).
25. Wang, P., Zhang, X., Zhao, G., Liu, Y. & Lu, Y. Oxidative coupling of methane: MO_x_-modified (M=Ti, Mg, Ga, Zr) Mn_2_O_3_-Na_2_WO_4_/SiO_2_ catalysts and effect of MO_x_ modification. *Chinese J. Catal.* **39**, 1395-1402 (2018).
26. Seubsai, A., Tiencharoenwong, P., Kidamorn, P. & Niamnuy, C. Synthesis of light hydrocarbons via oxidative coupling of methane over silica-supported Na_2_WO_4_-TiO_2_ catalyst. *Eng. J.* **23**, 169-182 (2019).
27. Hayek, N. S., Khlief, G. J., Horani, F. & Gazit, O. M. Effect of reaction conditions on the oxidative coupling of methane over doped MnO_x_-Na_2_WO_4_/SiO_2_ catalyst. *J. Catal.* **376**, 25-31 (2019).
28. Kim, J., Park, L.-H., Ha, J.-M. & Park, E. D. Oxidative coupling of methane over Mn_2_O_3_-Na_2_WO_4_/SiC catalysts. *Catalysts* **9**; doi:10.3390/catal9040363 (2019).
29. Chukeaw, T., Sringam, S., Chareonpanich, M. & Seubsai, A. Screening of single and binary catalysts for oxidative coupling of methane to value-added chemicals. *Mol. Catal.* **470**, 40-47 (2019).
30. Sringam, S., Kidamorn, P., Chukeaw, T., Chareonpanich, M. & Seubsai, A. Investigation of metal oxide additives onto Na_2_WO_4_-Ti/SiO_2_ catalysts for oxidative coupling of methane to value-added chemicals. *Catal. Today* **358**, 263-269 (2020).
31. Kidamorn, P. et al. Synthesis of value-added chemicals via oxidative coupling of methanes over Na_2_WO_4_-TiO_2_-MnO_x_/SiO_2_ catalysts with alkali or alkali earth oxide additives. *ACS Omega* **5**, 13612-13620 (2020).
